# Electronic nicotine delivery systems exhibit reduced bronchial epithelial cells toxicity compared to cigarette: the Replica Project

**DOI:** 10.1038/s41598-021-03310-y

**Published:** 2021-12-17

**Authors:** Massimo Caruso, Rosalia Emma, Alfio Distefano, Sonja Rust, Konstantinos Poulas, Fahad Zadjali, Antonio Giordano, Vladislav Volarevic, Konstantinos Mesiakaris, Mohammed Al Tobi, Silvia Boffo, Aleksandar Arsenijevic, Pietro Zuccarello, Cesarina Giallongo, Margherita Ferrante, Riccardo Polosa, Giovanni Li Volti, the Replica Project Group, Roberta Pulvirenti, Giuseppe Carota, Maria Rita Spampinato, Tancredi Caruso, Georgios Karanasios, Najwa Albalushi, Angelo Canciello, Aleksandar Ilic

**Affiliations:** 1 Department of Biomedical and Biotechnological Sciences, University of Catania, Via S. Sofia, 97, 95123 Catania, Italy; 2 Center of Excellence for the Acceleration of Harm Reduction (CoEHAR), University of Catania, Via S. Sofia, 97, 95123 Catania, Italy; 3 ECLAT Srl, spin-off of the University of Catania, Via S. Sofia 89, 95123 Catania, Italy; 4 Institute for Research and Innovation, IRIS, Patras Science Park, Patras, Greece; 5 Laboratory of Molecular Biology and Immunology, Department of Pharmacy, University of Patras, Patras, Greece; 6 College of Medicine and Health Sciences, Department of Clinical Biochemistry, Sultan Qaboos University, P.C 123, P.O. Box 35, Khodh, Oman; 7 Sbarro Institute for Cancer Research and Molecular Medicine, Department of Biology, College of Science and Technology, Temple University, Philadelphia, USA; 8 Center for Molecular Medicine and Stem Cell Research, Department of Microbiology and Immunology, Faculty of Medical Sciences, University of Kragujevac, 69 Svetozara Markovica Street, 34000 Kragujevac, Serbia; 9 Department of Medical, Surgical Sciences and Advanced Technologies “G.F. Ingrassia”, University of Catania, Via S. Sofia, 87, 95123 Catania, Italy; 10 Department of Clinical and Experimental Medicine, University of Catania, Via S. Sofia, 97, 95123 Catania, Italy

**Keywords:** Biochemistry, Biological techniques, Cell biology, Risk factors

## Abstract

Electronic nicotine delivery systems (ENDS) may reduce health risks associated with chronic exposure to smoke and their potential benefits have been the matter of intense scientific debate. We aimed to replicate three published studies on cytotoxic and inflammatory effects of cigarette smoke and ENDS aerosol in an independent multi-center ring study. We aimed to establish the reliability of results and the robustness of conclusions by replicating the authors’ experimental protocols and further validating them with different techniques. Human bronchial epithelial cells (NCI-H292) were exposed to cigarette whole smoke and vapor phase and to aerosol from ENDS. We also assessed the inflammatory cytokines interleukin-6 and interleukin-8 and the remodeling mediator matrix metalloproteinase-1. We replicated cell viability results and confirmed that almost 80% of cytotoxic effects are due to volatile compounds in the vapor phase of smoke. Our findings substantiated the reduced cytotoxic effects of ENDS aerosol. However, our data on inflammatory and remodeling activity triggered by smoke differed significantly from those in the original reports. Taken together, independent data from multiple laboratories clearly demonstrated the reduced toxicity of ENDS products compared to cigarettes.

## Introduction

Cigarette smoking is a major risk factor for many pathological conditions, including cardiovascular and respiratory diseases and lung cancer (National Center for Chronic Disease Prevention and Health Promotion (US) Office on Smoking and Health 2014). WHO estimates that more than 7 million people die each year from smoking combustible tobacco products, making smoking the leading cause of preventable deaths worldwide. Commercially available innovative non-combustible technologies, such as electronic cigarettes (e-cigarettes) and tobacco-heating products (THPs), often referred to as electronic nicotine delivery systems (ENDS), may reduce the burden of smoking-related morbidity and mortality by substantially reducing exposure to the harmful compounds in cigarette smoke. The potential benefits and risks of using ENDS have been the matter of intense scientific debate^1^. Toxicological evidence is, therefore, needed to confirm the effects and to ensure protection at the individual and public health levels^2^.

In 2007, the Committee on Toxicity Testing and Assessment of Environmental Agents^2^ proposed a strategy for toxicity testing. In particular, they established that the goals of such testing should be to identify pathways that, when perturbed, can lead to adverse health outcomes and to evaluate host susceptibility to such effects. Among the most important pre-clinical studies on the effect of ENDS with respect to tobacco cigarette smoke are the studies conducted by the tobacco companies due to their expertise and compliance with high quality standards by carrying out studies for regulatory purposes. Stand-out studies have evaluated the cytotoxicity, genotoxicity, and mutagenicity induced by smoke and compared with ENDS aerosol on cultured cell models of animal lung cells^3^, human bronchial epithelium^4–6^, endothelial cells^7,8^, and immune cells^9^ and assessed inflammation^10,11^ and oxidative stress responses^12–14^.

Herein we report a multi-site replication study (ring study) to verify the results of three key published studies performed by the tobacco industry regarding cytotoxicity induced by tobacco smoke and ENDS aerosols on a model of airway epithelial cells^4,10,15^. We aimed to reproduce the reported methodological approaches and assess the reliability of measurements and robustness of conclusions.

## Results

### Laboratory performances for 1R6F dose response

Large variability in NRU cell viability was observed within LAB-D and LAB-E in performing ISO WS exposure compared to the other laboratories (see Figure S1A in supplementary information). Substantial interlaboratory (SR and SB) variability (defined as > 20 deviations) was observed for all ISO WS exposure conditions (see Figure S1B in supplementary information). Linear regression analyses of ISO WS dose–response curves showed good reproducibility between LAB-A and LAB-B (r = 0.871; *p* = 0.002), LAB-A and LAB-C (r = 0.774; *p* = 0.009), and LAB-C and LAB-B (r = 0.67; *p* = 0.013). No significant correlations were seen between LAB-D or LAB-E and the other laboratories.

LAB-D showed more variability in performing ISO VP exposure than other laboratories (see Figure S2A in supplementary information). Interlaboratory variability (SR and SB) was notable, with ISO VP exposure under the two, five, 10, 12, and 25 puffs conditions and within-laboratory variability (Sr) was observed for two and five puffs (see Figure S2B in supplementary information). Linear regression analyses of ISO VP dose–response curves showed good reproducibility between LAB-A and LAB-B (r = 0.917; *p* < 0.001), LAB-A and LAB-C (r = 0.946; *p* < 0.0001), LAB-A and LAB-E (r = 0.715; *p* = 0.008), LAB-B and LAB-E (r = 0.587; *p* = 0.027), LAB-C and LAB-B (r = 0.889; *p* < 0.001), and LAB-C and LAB-E (r = 0.69; *p* = 0.011). LAB-D showed no significant correlation with any other laboratory.

Variability in the results for HCI WS exposure was observed only for two and four puffs in LAB-D (see Figure S3A in supplementary information). However, interlaboratory (SR and SB) variability was observed for two, four, five, six, and eight puffs under this regimen (see Figure S3B in supplementary information). Linear regression analyses of HCI WS dose–response curve results showed good reproducibility between LAB-A and LAB-B (r = 0.77; *p* = 0.004), LAB-A and LAB-C (r = 0.586; *p* = 0.027), LAB-A and LAB-D (r = 0.729; *p* = 0.007), LAB-A and LAB-E (r = 0.763; *p* = 0.005), LAB-B and LAB-E (r = 0.677; *p* = 0.012), LAB-B and LAB-D (r = 0.745; *p* = 0.006), LAB-C and LAB-E (r = 0.624; *p* = 0.02), and LAB-D and LAB-E (r = 0.636; *p* = 0.018).

For cell viability after exposure to 1R6F VP under the HCI regimen, variability was greatest for LAB-C compared to other laboratories (see Figure S4A in supplementary information) and interlaboratory variability (SR and SB) was seen for eight, 10, and 15 puffs (see Figure S4B in supplementary information). Linear regression analyses of HCI VP dose response curve results showed good reproducibility between LAB-A and LAB-B (r = 0.861; *p* = 0.003), LAB-A and LAB-D (r = 0.8557; *p* = 0.003) LAB-A and LAB-E (r = 0.671; *p* = 0.024), LAB-B and LAB-E (r = 0.663; *p* = 0.014), LAB-B and LAB-D (r = 0.94; *p* < 0.0001), LAB-B and LAB-E (r = 0.714; *p* = 0.008), and LAB-D and LAB-E (r = 0.702; *p* = 0.009).

All the regression and Bland‒Altman plots are provided in supplementary information (see Figs. S5–S44).

### Experiment 1: effect of WS and VP on NCI-H292 cell viability

Based on laboratory performance results, LAB-D and LAB-E NRU data of ISO WS exposure, LAB-B data of ISO VP exposure, and LAB-C data of HCI VP exposure were excluded from IC50 determination.

**Figure 1:**
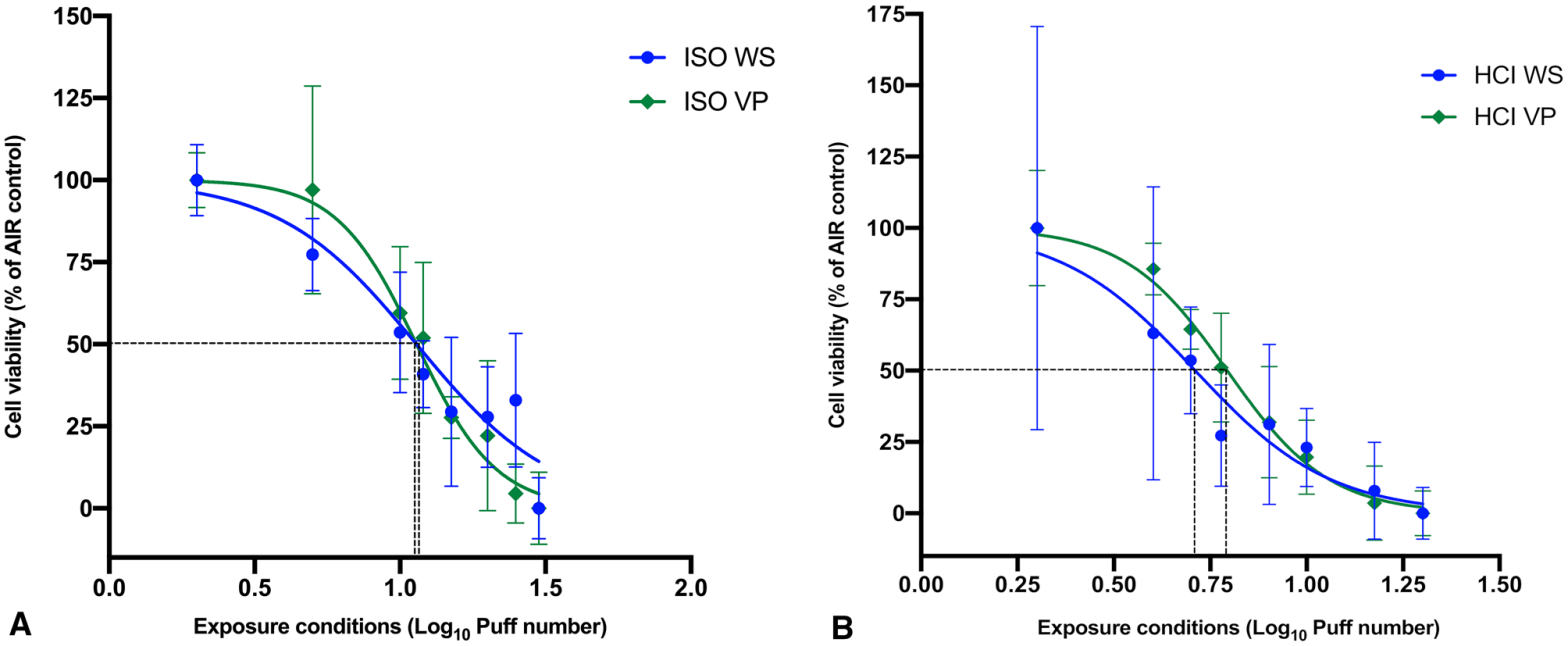
Cytotoxicity of NCI-H292 cells after WS and VP exposure. (**A**) Exposure under the ISO regimen. Data points are mean ± SD from 10 replicates of WS and VP. There was no significant (*p* = 0.098) increase in IC50 for VP exposure (11.76 puffs; Log10 = 1.07) versus WS exposure (10.47 puffs; Log10 = 1.02). (**B**) Exposure under the HCI regimen. Data points are mean ± SD from 16 replicates for WS and 10 replicates for VP. There was a significant (*p* = 0.046) increase in IC50 for VP exposure (6.22 puffs; Log10 = 0.79) compared to WS (5.14 puffs; Log10 = 0.71) exposure. Abbreviations: HCI, Health Canada intense; ISO, International Organization for Standardization; VP, vapor phase; WS, whole smoke. Graphs were generated with GraphPad Prism 8 software and edited by using GIMP image manipulation program (version 2.10.14).

Under the ISO regimen, 1R6F WS led to decreased cell viability from two to 30 puffs, giving an IC50 value of 10.47 puffs. VP exposure also decreased cell viability from two puffs to 30 puffs, but with an IC50 value of 11.76 puffs. Thus, the IC50 for WS exposure was reduced by about 12% compared with that following VP exposure (Fig. 1A), but the difference was not significant (*p* = 0.098).

Under the HCI regimen, 1R6F WS decreased cell viability from two to 20 puffs and the IC50 value was 5.14 puffs. For VP exposure, cell viability also decreased from two puffs to 20 puffs but the IC50 was higher at 6.22 puffs. The IC50 with WS exposure was, therefore, significantly reduced by about 21% compared with VP exposure (*p* = 0.046; Fig. 1B).

### Experiment 1: effect of WS and VP on inflammatory and remodeling mediators

For the analysis of inflammatory and remodeling mediators, based on NRU laboratory performance, LAB-D and LAB-E data of ISO WS exposure, LAB-D data of ISO VP exposure, and LAB-C data of HCI VP exposure were excluded. LAB-E provided inconsistent inflammatory mediator results, and these were also excluded from analyses.

**Figure 2:**
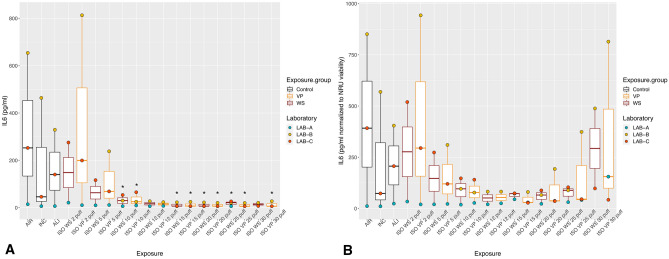
IL-6 inflammatory mediator secretion following ISO WS and VP exposures, by exposure condition. Data are median, IQR, and range, and the laboratory color coded points represent the mean of each laboratory results. (**A**) IL6 concentrations. (**B**) IL6 concentration normalized to NRU viability. Abbreviations: AIR, air control; ALI, air–liquid interface control; INC, incubator control; ISO, International Organization for Standardization; IL-6, interleukin 6; VP, vapor phase; WS, whole smoke. *p < 0.05 compared to air control. Graphs were generated with R version 3.4.3 (2017-11-30) and edited by using GIMP image manipulation program (version 2.10.14).

**Figure 3:**
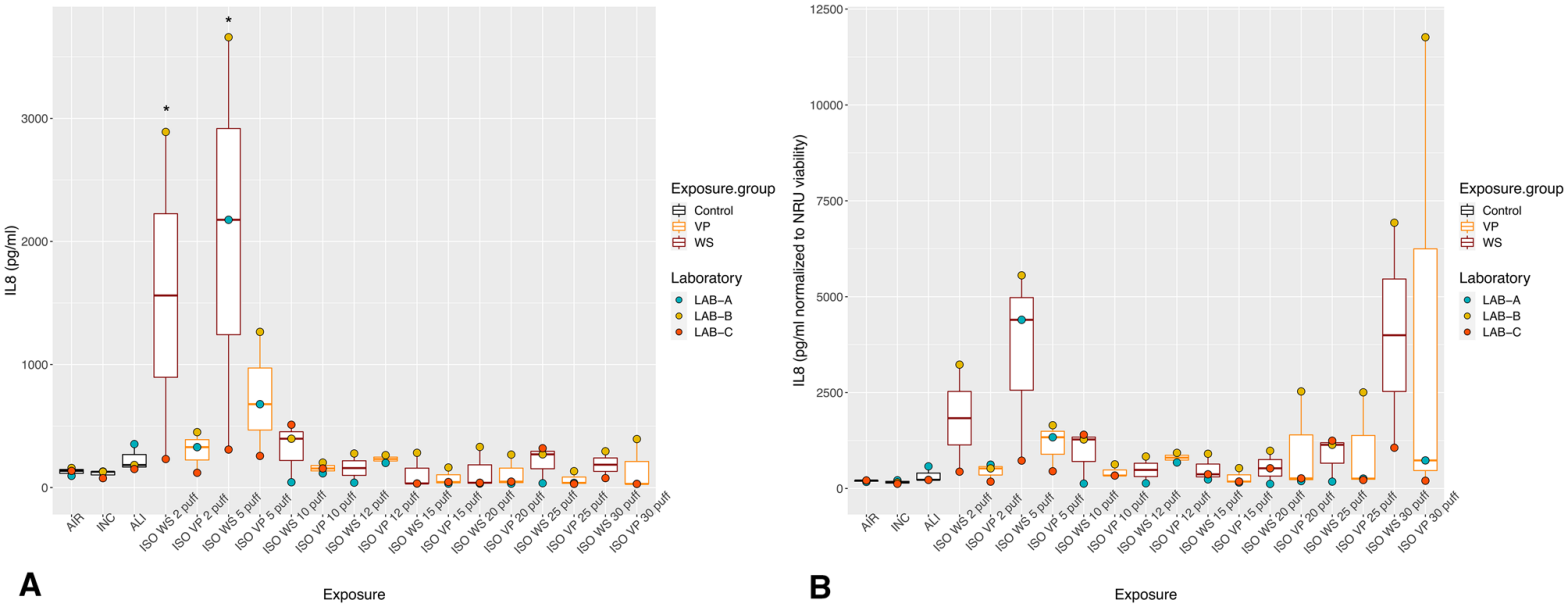
IL-8 inflammatory mediator secretion following ISO WS and VP exposures, by exposure condition. Data are median, IQR, and range, and the laboratory color coded points represent the mean of each laboratory results. (**A**) IL8 concentration. (**B**) IL8 concentration normalized to NRU viability. Abbreviations: AIR, air control; ALI, air–liquid interface control; INC, incubator control; ISO, International Organization for Standardization; IL-8, interleukin 8; VP, vapor phase; WS, whole smoke. *p < 0.05 compared to air control. *p < 0.05 compared to air control. Graphs were generated with R version 3.4.3 (2017-11-30) and edited by using GIMP image manipulation program (version 2.10.14).

**Figure 4:**
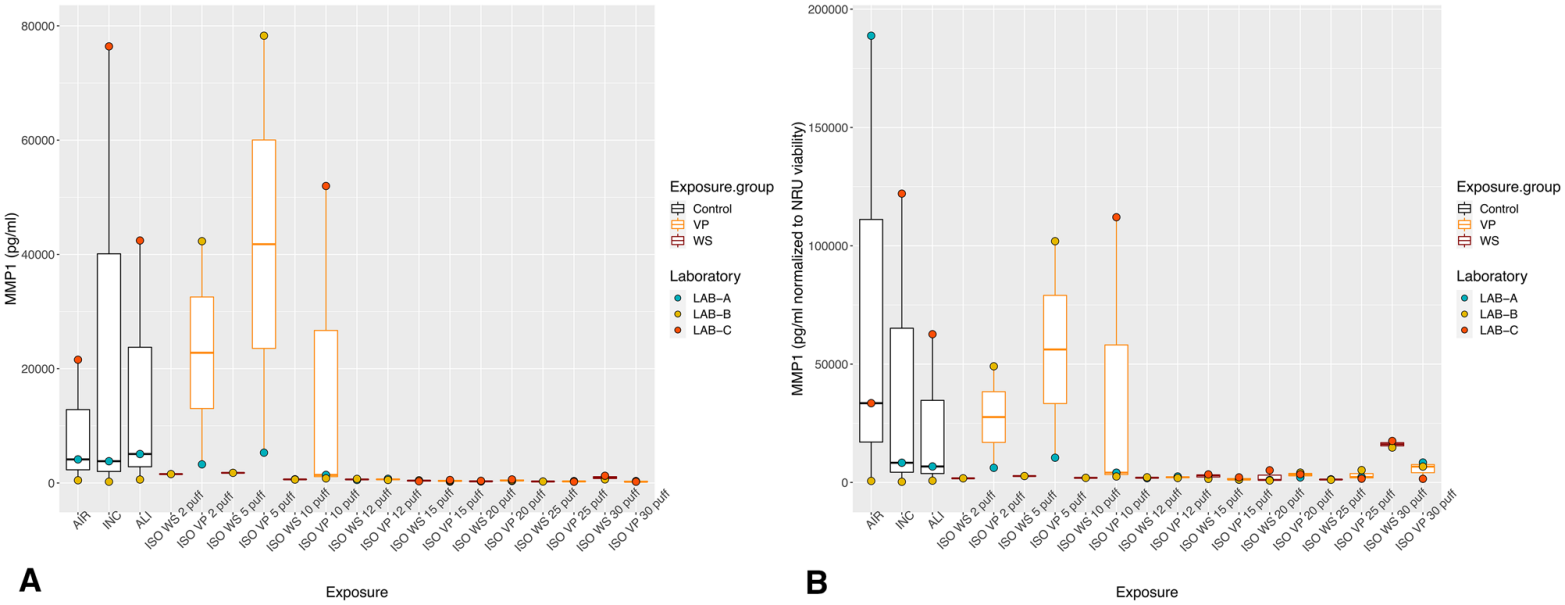
MMP-1 tissue remodeling mediator secretion following ISO WS and VP exposures, by exposure condition. Data are median, IQR, and range, and the laboratory color coded points represent the mean of each laboratory results. (**A**) MMP-1 concentrations. (**B**) MMP-1 concentrations normalized to NRU viability. Abbreviations: AIR, air control; ALI, air–liquid interface control; INC, incubator control; ISO, International Organization for Standardization; MMP-1, matrix metalloproteinase 1; VP, vapor phase; WS, whole smoke. Graphs were generated with R version 3.4.3 (2017-11-30) and edited by using GIMP image manipulation program (version 2.10.14).

With exposure to WS and VP under the ISO regimen, all the inflammatory mediators showed the highest values with the lower puff numbers, which decreased as the puff number increased (Figs. 2A, 3A, 4A). Concentrations did not differ significantly between WS and VP for any inflammatory mediator. IL-6 concentrations at the highest puff numbers (> 10 puffs) of WS and VP were significantly lower than those seen in the air control (*p* < 0.005; Fig. 2A). For IL-8, only concentrations released at two and five puffs of WS were significantly increased (both *p* < 0.001) when compared to the air control (Fig. 3A). MMP-1 concentrations did not differ from the air control after any number of puffs (Fig. 4A).

When IL-6 and IL-8 concentrations were normalized to NRU cell viability, concentrations increased at higher puff numbers (Figs. 2B, 3B). Moreover, IL6 and IL8 concentrations following ISO WS exposure seemed to be higher than after VP exposure, but not significantly so. No significant variations were observed when NRU cell viability normalization was applied to MMP-1 results (Fig. 4B).

**Figure 5:**
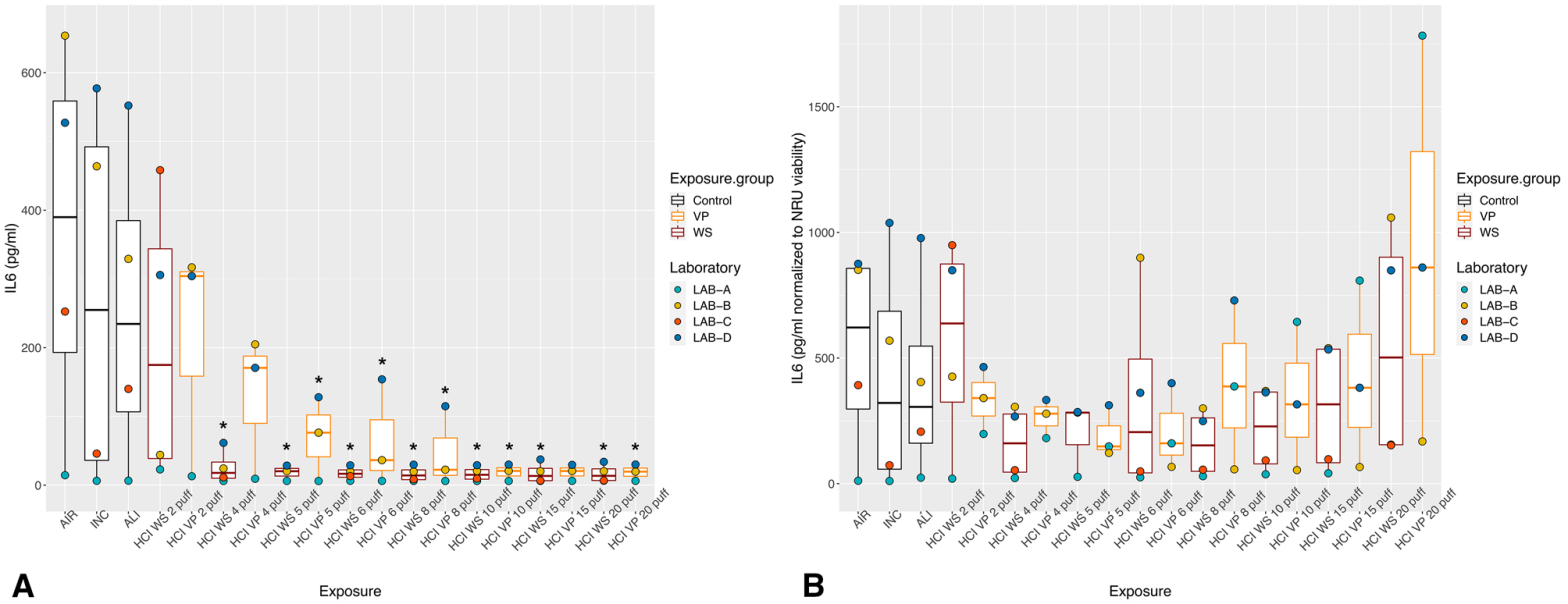
IL-6 inflammatory mediator secretion following HCI WS and VP exposures, by exposure condition. Data are median, IQR, and range, and the laboratory color coded points represent the mean of each laboratory results. (**A**) IL-6 concentrations. (**B**) IL-6 concentration-normalized to NRU viability. Abbreviations: AIR, air control; ALI, air–liquid interface control; HCI, Health Canada intense regimen; IL-6, interleukin 6. INC, incubator control with apical media; NRU, neutral red uptake; VP, vapor phase; WS, whole smoke. *p < 0.05 compared to air control. Graphs were generated with R version 3.4.3 (2017-11-30) and edited by using GIMP image manipulation program (version 2.10.14).

**Figure 6:**
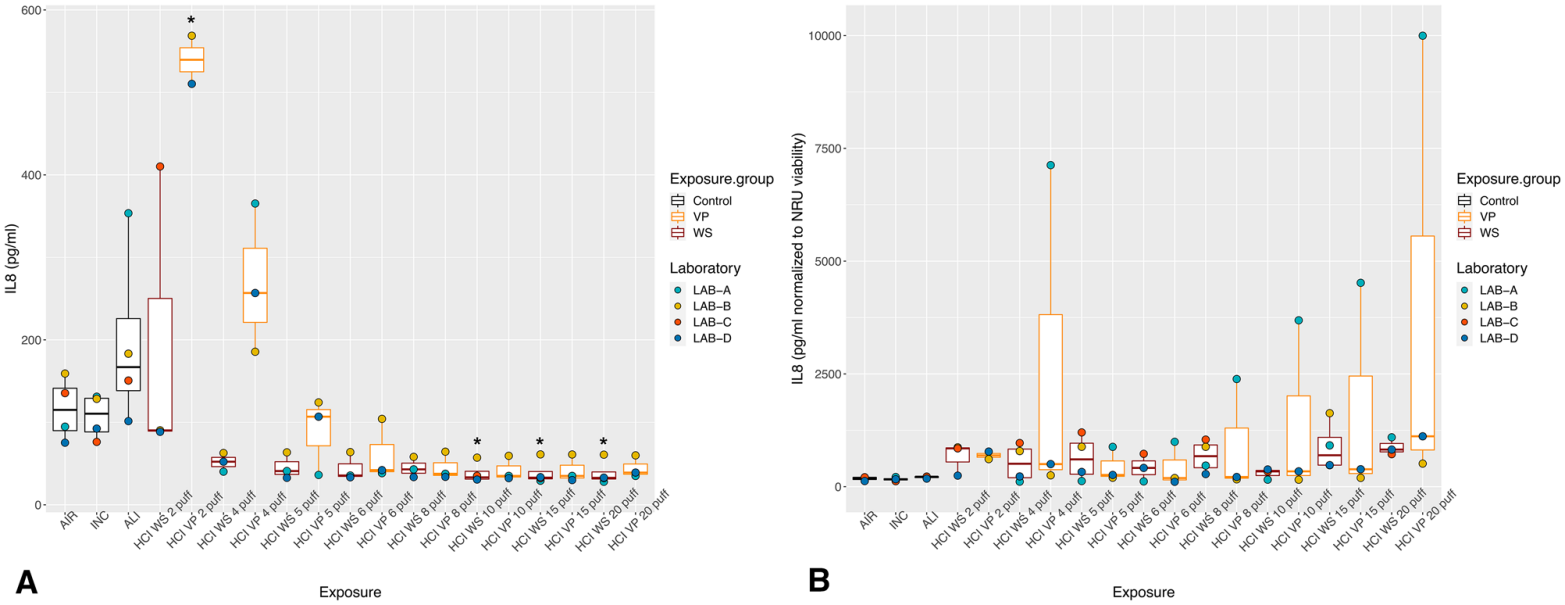
IL-8 inflammatory mediator secretion following HCI WS and VP exposures, by exposure condition. Data are median, IQR, and range, and the laboratory color coded points represent the mean of each laboratory results. (**A**) IL8 concentrations. (**B**) IL-8 concentration-normalized to NRU viability. The laboratory color coded points represent the mean of each laboratory result. Abbreviations: AIR, Air control; ALI, air–liquid interface control; HCI, Health Canada intense regimen; IL-8, interleukin 8. INC, incubator control with apical media; NRU, neutral red uptake; VP, vapor phase; WS, whole smoke. *p < 0.05 compared to air control. Graphs were generated with R version 3.4.3 (2017-11-30) and edited by using GIMP image manipulation program (version 2.10.14).

**Figure 7:**
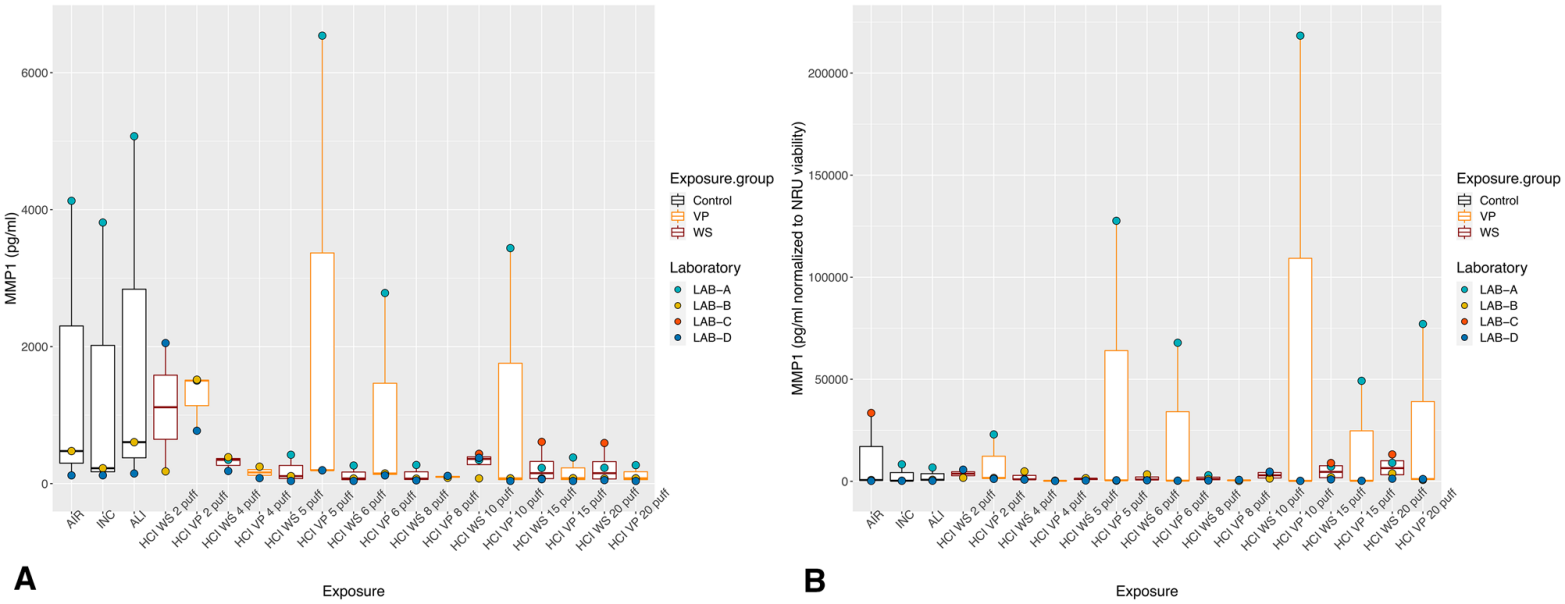
MMP-1 tissue remodeling mediator secretion following HCI WS and VP exposures, by exposure condition. Data are median, IQR, and range, and the laboratory color coded points represent the mean of each laboratory results. (**A**) MMP-1 concentrations. (**B**) MMP-1 concentration-normalized to NRU viability. The laboratory color coded points represent the mean of each laboratory result. Abbreviations: AIR: Air control, ALI, air–liquid interface control; HCI, Health Canada intense regimen; INC: Incubator control with apical media, NRU, neutral red uptake; VP, vapor phase; WS: Whole smoke. Graphs were generated with R version 3.4.3 (2017-11-30) and edited by using GIMP image manipulation program (version 2.10.14).

Under the HCI regimen, IL6, IL8, and MMP-1 concentrations were also highest with lower puff numbers and decreased as puff numbers increases (Figs. 5A, 6A, 7A). Although release seemed slightly higher with VP exposures than WS exposures for all the inflammatory mediators, they did not differ significantly. IL-6 concentrations at nearly all WS and VP puff numbers were significantly lower than those seen in the air control (*p* < 0.05; Fig. 5A). IL-8 release at two puffs of VP was significantly increased compared to that with the air control (*p* < 0.001; Fig. 6A). When IL-6 and IL-8 concentrations were normalized to NRU cell viability, concentrations changed to being greater with higher puff numbers (Figs. 5B, 6B). For MMP-1, only slight variations were observed when NRU cell viability normalization was applied (Fig. 7B).

### Experiment 1: nicotine dosimetry

**Figure 8:**
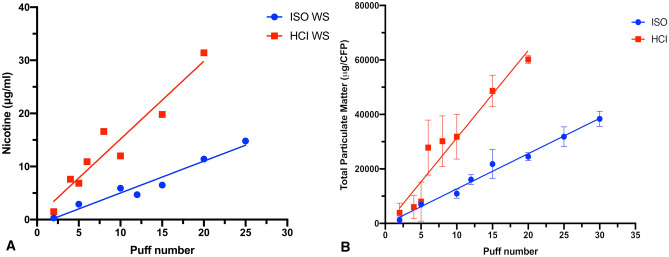
Nicotine and total particulate matter (TPM) release from WS by machine smoking regimen. (**A**) Nicotine (μg/ml) release by number of puffs. Concentrations differed significantly between the ISO and HCI regimens (*p* = 0.0005). (**B**) Total particulate matter (μg/CFP) release by number of puffs. There was a statistical difference in TPM release between ISO and HCI exposure (*p* < 0.0001). Abbreviations: HCI, Health Canada intense regimen; ISO, International Organization for Standardization regimen; WS, whole smoke. Graphs were generated with GraphPad Prism 8 software and edited by using GIMP image manipulation program (version 2.10.14).

Only WS exposure led to a detectable quantity of nicotine in the culture media. We observed an increase in nicotine concentration with increasing puff number, and more with the HCI regimen than with the ISO regimen (*p* = 0.0005; Fig. 8A). A similar trend was observed when the TPM was presented against puff number exposure. The TPM release under HCI regimen was also significantly increased compared to ISO regimen (*p* < 0.0001; Fig. 8B).

**Figure 9:**
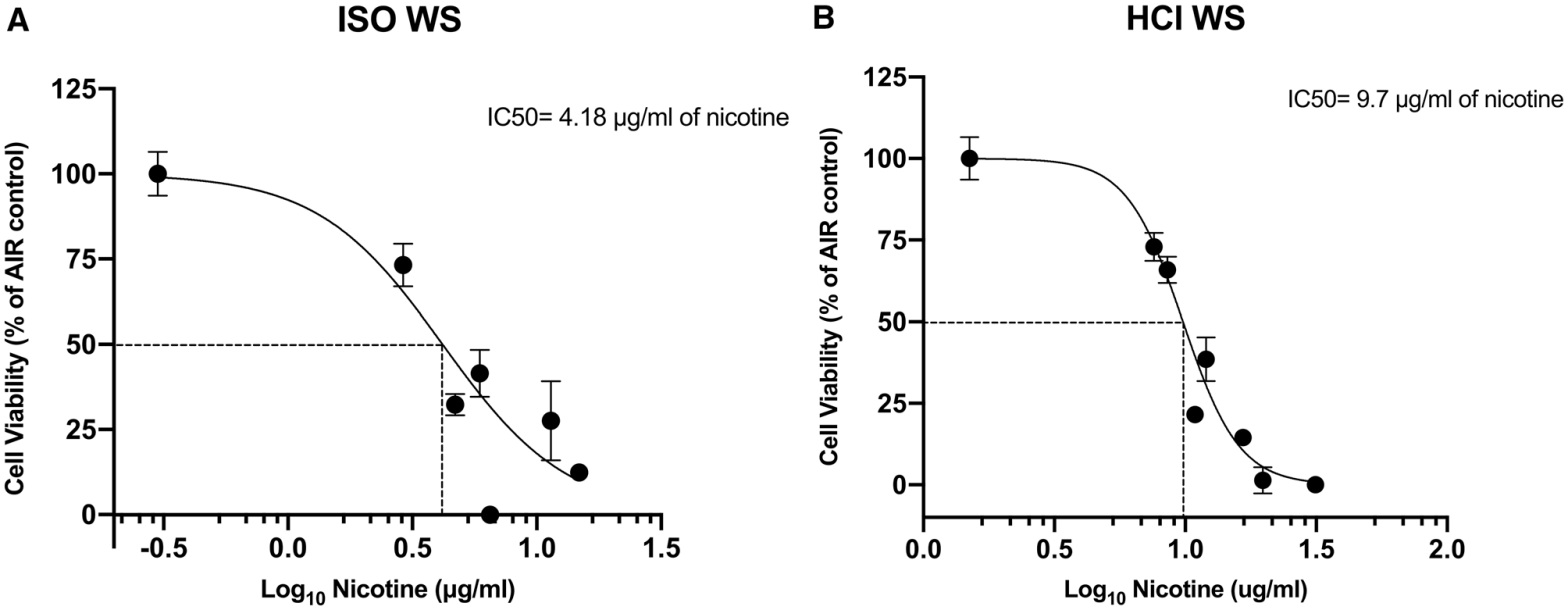
Calculation of IC50 for nicotine released by WS. (**A**) ISO regimen, showing IC_50_ of 4.18 μg/mL nicotine (Log10 = 0.62). (**B**) HCI regimen, showing IC_50_ of 9.7 μg/ml of nicotine (Log10 = 0.99). Data points are mean ± SD values from four replicates (LAB-A). Biological response data are presented as a function of nicotine concentration in the exposed media. Abbreviations: HCI, Health Canada intense; ISO, International Organization for Standardization; WS, whole smoke. Graphs were generated with GraphPad Prism 8 software and edited by using GIMP image manipulation program (version 2.10.14).

Using nicotine dosimetry data from WS exposures, cell viability of the biological responses was presented against exposed nicotine in the cell media for each puff number. Therefore, the IC50 for nicotine from WS under the ISO regimen was 4.18 μg/mL and was roughly the amount of nicotine released by 10 puffs (Fig. 9A). Under the HCI regimen, the IC50 was 9.7 μg/mL and was reached after about five puffs (Fig. 9B).

Based on calculated nicotine IC50 dose by WS under the HCI regimen (9.7 μg/ml), we chosen to conduct the experiments 2 and 3 of this study using the number of puffs able to produce the amount of nicotine closest to this value across all products (1R6F, IQOS, glo, ePen, and eStick). Thus, the number of puffs for 1R6F WS was set at five (mean nicotine concentration 8.55 ± 0.78 μg/mL), at 25 puffs for Vype eStick (8.8 ± 2.4 μg/mL), at 10 puffs for Vype ePen 3 (8.47 ± 1.54 μg/mL), at eight puffs for glo PRO (8.7 ± 1.3 μg/mL) and at seven puffs for IQOS 3 DUO (9.03 ± 1.31 μg/mL).

### Experiments 2 and 3: laboratory performances for exposure to 1R6F, THPs, and e-Cigs

**Figure 10:**
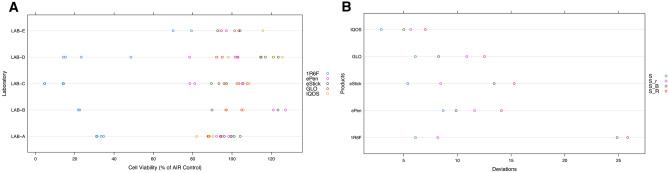
Laboratory performances for exposure to all study products. (**A**) Measurements of NRU cell viability, and (**B**) measures of variability for each test product. Values were measured at five puffs for 1R6F, 25 puffs for Vype eStick, 10 puffs for Vype ePen 3, eight puffs for glo PRO, and seven puffs for IQOS 3 DUO. Abbreviations: S, global deviation of all laboratories; SB, interlaboratory deviation between means; Sr, intra-laboratory deviation from repeatability; SR, interlaboratory deviation from reproducibility. Graphs were generated with R version 3.4.3 (2017-11-30) and edited by using GIMP image manipulation program (version 2.10.14).

We evaluated laboratory performance for each exposure condition and product variability. Intra-laboratory variability is low across all laboratories and products, except for 1R6F exposure performed by LAB-D (Fig. 10A). 1R6F exposure showed the greatest interlaboratory variability. Indeed, interlaboratory deviations (SB and SR) were greatest for 1R6F compared to other products (Fig. 10B).

### Experiment 2 and 3: effect of THPs and e-Cig exposure on H292 cell viability compared to 1R6F exposure

**Figure 11:**
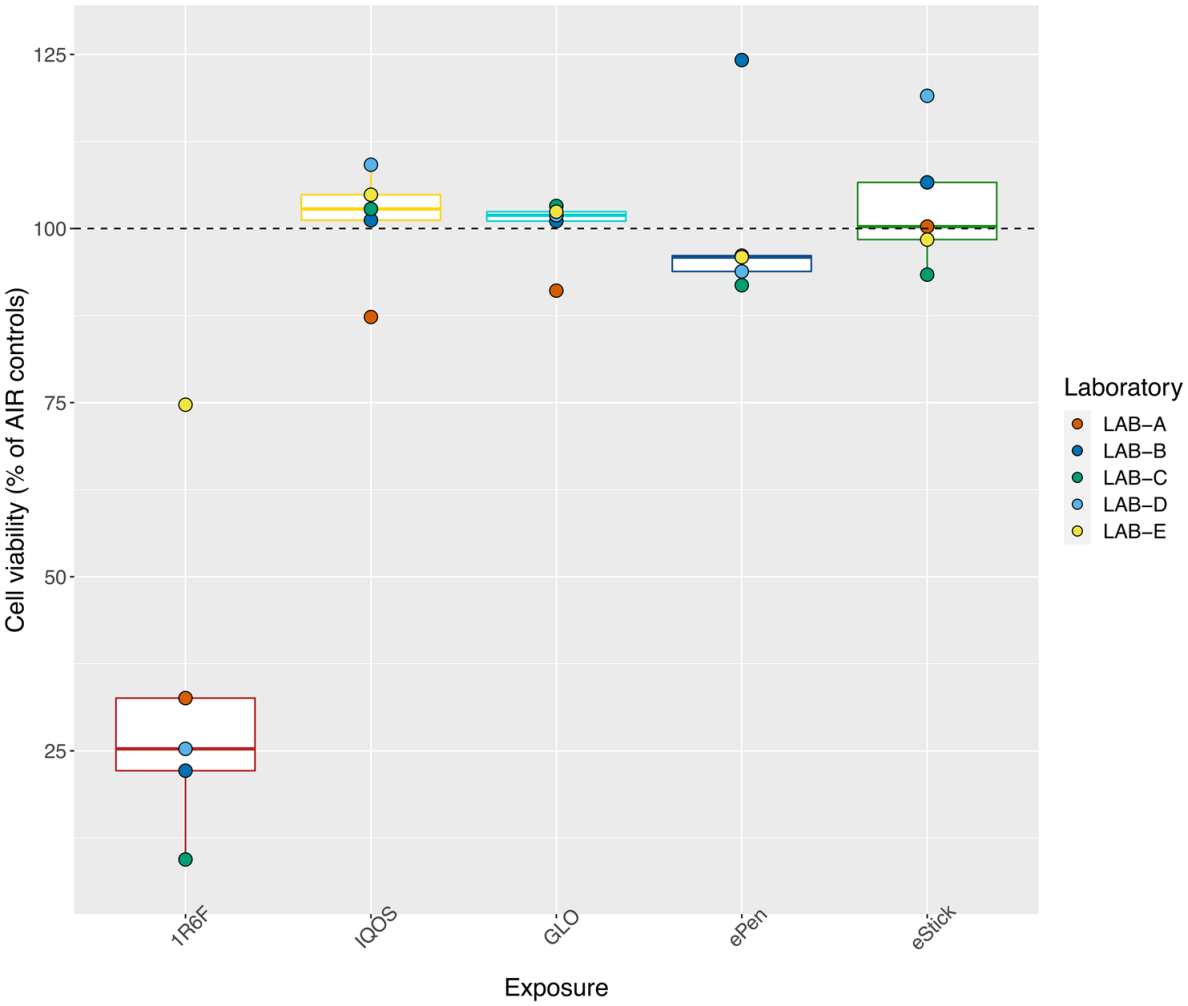
Comparison of NRU cell viability after exposure to 1R6F WS or ENDS aerosol. Data are median (IQR) percentages of the air control, and the laboratory means are shown by color coded points. 1R6F 26.45% (IQR 14.5‒33.1, *p* = 0.009); IQOS 93.34% (88.2‒103.1); glo PRO 95.04% (89.6‒103.3); Vype ePen 3 97.57% (92.2‒102.3); and Vype eStick 101.09% (96.1‒114.5). Abbreviations: ENDS, electronic nicotine delivery devices; NRU, neutral red uptake assay; WS, whole smoke. Graphs were generated with R version 3.4.3 (2017-11-30) and edited by using GIMP image manipulation program (version 2.10.14).

There was an overall significant difference in NRU cell viability among all product compared with the air control (*p* < 0.0001), driven mainly by the effect of 1R6F cigarettes (Fig. 11). No reduction in cell viability was observed after exposure to THPs or e-cigarettes. Thus, in comparisons between products only 1R6F differed significantly from other products (*p* < 0.0001).

## Discussion

In the present multi-center ring study, we aimed to verify the robustness and reliability of the results obtained from three relevant tobacco industry studies on in vitro cytotoxicity of tobacco cigarettes and ENDS in NCI-H292 cells. The experiments could be replicated in multiple laboratories, albeit with some differences in devices and equipment due to the rapid evolution of investigational products, commercial products, and laboratory methods. Despite some variability within and between laboratories, all conditions could be tested in multiple laboratories. Experiment 1, which assessed cytotoxicity and inflammation of WS and VP at the ALI in a cell culture model, was chosen because its simple protocol and could be used to fine-tune and harmonize the collaboration among participating laboratories improving accuracy and reliability. The results obtained in our laboratories confirmed those obtained by Azzopardi et al.^10^, showing comparable differences in the cytotoxicity induced by cigarette WS and VP. These findings confirmed that the acute cytotoxicity is due mainly to effects of the volatile components of cigarette smoke on the cells of the lung epithelium rather than by TPM or nicotine. In our comparison of the cytotoxicity induced by conventional cigarette smoke and the aerosol of e-cigarettes in experiment 2, we ensured that the cell culture was exposed to similar amounts of nicotine from all products. We found no toxicity to lung cells from e-cigarette aerosol, supporting the findings of Azzopardi et al.^7^. Finally, experiment 3, in which cytotoxicity induced by THPs was compared with that induced by cigarette smoke, also indicated substantially reduced toxicity. We added a direct comparison of e-cigarettes to THPs not included in the original studies and observed no cytotoxicity on bronchial epithelial cells with any ENDS product.

Tobacco smoke is well known to induce an imbalance between oxidants and antioxidants in the airways, leading to oxidative stress^16^, increased mucosal inflammation, and increased expression of inflammatory cytokines (e.g., IL-6 and IL-8)^17^. Azzopardi and colleagues^10^ found that air exposure increased production of IL-6 and IL-8 by three to four times compared to the ALI and INC controls. By contrast, we found that air exposure increased the production of the inflammatory cytokines only twofold. Additionally, Azzopardi and colleagues reported that exposure of NCI-H292 cells to WS increased the quantity of IL-6 and IL-8 released from cells whereas exposure to the VP did not. They concluded, therefore, that the proinflammatory stimulus was due mainly to the particulate components of the smoke. In our study we observed a significant dose-dependent decrease of IL-6 release from bronchial epithelial cells exposed to WS compared to air exposure, irrespective of smoking regimen. We also noted greater production of IL-6 by the cells exposed to VP than by those exposed to WS with both smoking regimens, but the values were always lower than with exposure to air. Thus, unexpectedly, our results on IL-6 seemed to indicate an anti-inflammatory effect of smoke. Normalizing the quantity of IL-6 released with the NRU assay cell viability values, we found that IL-6 production increased with the HCI regimen but only overtook the concentrations in the air exposure control after 20 puffs. This finding clarified that NCI-H292 cells need more consistent or prolonged stimulus to activate IL-6 release (Fig. 5). Probably, direct exposure of cells to undiluted smoke has an exacerbating effect on bronchial cells, generating strong cytotoxicity, initially deactivating the cellular machinery, and delaying the response to the insult presented by smoking. In more prolonged and extreme conditions of exposure (HCI, 20 puffs), the proinflammatory response also seems to appear, but it seems to be greatly dependent on the presence of volatile components.

Azzopardi and colleagues^10^ reported an increased production of IL-8 in air exposed NCI-H292 cells compared to INC and ALI controls, and a greater production of IL-8 under the HCI regimen than under the ISO regimen. We observed that the concentrations of IL-8 did not differ significantly between the three controls (ALI, INC, and air), but we observed a significant increase in cytokine production by NCI-H292 cells exposed to two and five puffs of WS under the ISO regimen and to two puffs of VP under the HCI regimen that decreased substantially thereafter. In accordance, the delivery of WS was more effective under the ISO regimen, whereas VP was more effective under the HCI regimens. As for IL-6, normalization of the data to cell viability altered the pattern of release to increasing with rising number of puffs. Finally, in Azzopardi et al.^10^ and our study, no significant differences were seen in MMP-1 between the three controls and only a slight increase in response to VP after two and five under the ISO regimen. Cigarette smoke induces MMP-1 mRNA and protein expression in human airway cells^18^, which induces excessive matrix remodeling in smokers and leads to emphysema. However, our findings support the theory that direct exposure to undiluted smoke in vitro is too extreme to assess the cell responses. Therefore, models of exposure involving smoke diluted with air, as done by Azzopardi and colleagues^10^, could be a better approach.

Azzopardi et al.^10^ reported that cytotoxicity measured by EC_50_ was 1:54 (smoke:air, vol:vol) with WS and 1:46 for VP (both *p* < 0.005) using the ISO regimen. The average difference in cell viability between the two dose–response curves was 11%, indicating that VP smoke constituted 89% of the total toxicity. In our study the IC_50_ for WS exposure was roughly 12% lower than that for VP under the ISO regimen and about 21% lower under HCI regimen, meaning that VP constituted 88% and 79%, respectively, of total toxicity. Despite the difference in the reference cigarettes used (3R4F *vs.* 1R6F) and method of exposure (air-diluted smoke *vs* undiluted smoke), our findings confirm that most smoke-induced cytotoxic damage to bronchial epithelial cells is due to the substances contained in the VP.

Our findings differed from those of Azzopardi et al.^10^ regarding TPM release, nicotine release, and cytotoxicity under the ISO and HCI regimens. In particular, while Azzopardi et al. reported no differences between regimens for TPM and nicotine production, we observed significantly greater production with the HCI regimen. For cytotoxicity associated with WS, they reported that the ISO regimen was significantly more toxic whereas we found the HCI regimen to be twice as toxic, reaching IC_50_ after 10 puffs under ISO regimen versus five puffs under HCI regimen. As the IC_50_ nicotine concentration with the ISO regimen was 4.18 μg/mL after 10 puffs compared with 9.7 μg/mL after five puffs with the HCI regimen, we conclude that nicotine is not directly responsible for the cytotoxic effect.

In Azzopardi and colleagues’^7^ comparison of cytotoxic effects of cigarette smoke versus aerosol from e-cigarettes, they observed that the latter was 97% less cytotoxic than cigarette smoke when matched for EC_50_ values at different dilutions and deposited 70% less nicotine. In accordance, in our study the cytotoxicity of Vype ePen 3 aerosol was > 71% lower than with undiluted 1R6F smoke and that of Vype eStick was more than > 74% lower. This evidence confirms the results obtained by Azzopardi et al. (2016) and adds the effects of undiluted smoke and aerosol to the data.

Jaunky and colleagues^15^ reported a significant difference in cytotoxic effects on NCI-H292 cells between cigarette smoke and THP aerosol, with the latter being associated with > 87% viability relative to 3R4F smoke at a common aerosol dilution (1:40, aerosol:air). In our experiment 3, exposure of cells to undiluted smoke or THP aerosol, adjusted to reach the same amount of nicotine in media, were associated with 26.45% viability with smoke exposure versus 93.34% with IQOS and 95.04% with glo PRO compared to the air control. Thus, cell viability was > 67% with THPs.

The main results obtained by Azzopardi et al.^4,10^ and Jaunky et al.^15^ proved to be reproducible, confirming the industry conclusions on cytotoxicity of their products. By contrast, results on inflammatory and remodeling markers were not reproduced. This does not automatically mean that conclusions derived from the result of original papers are false, but it does mean that conclusions and/or methods should be reconsidered and the subject of further investigations. In our opinion, normalization of the raw data to cell viability would be appropriate for interpretation of data on inflammatory mediators because they are actively produced and released by cells as a response to an exogenous stimulus. Nevertheless, normalization of data to better reflect cell functionality rather than viability would provide more correct data and should be considered for future evaluations in this direction. Additionally, we observed marked changes in morphology (Table S2, Figure S45) and increased expression of two key cells inflammatory markers (HLA-DR and CD206) (Table S3, Figure S46) on bronchial epithelial cells exposed to 1R6F smoke, differently from the cells exposed to ENDS aerosol.

Although our study was performed with a different smoking machine and vaping machine setup, exposing cells to undiluted smoke/aerosol rather than air-diluted smoke/aerosol, the results obtained on cytotoxicity in our study are very similar to those seen in the original studies. A limitation of our study was there was some variation in results between the different laboratories. However, conversely, it is a strength of the study that the repetition of the experiments in five different laboratories allowed us to obtain robust data.

In our opinion, this replication study can allay doubts about the results and proper execution of these three relevant studies by Azzopardi et al. (2015, 2016) and Jaunky et al. (2018). Our results support the reduced potential of e-cigs relative to conventional cigarettes in an in vitro model of bronchial epithelial cells, even under more extreme exposure conditions (undiluted aerosol) than those of replicated papers. For future studies, our findings suggest that aerosol dilution and normalization of data to better reflect cell functionality rather than viability will be important for achieving the most accurate results. Furthermore, future studies are now warranted for further demostrating also in vitro the chronic effects and reduced risk of ENDS. Indeed, Ghosh et al.^19^ showed chronic vaping exerts marked biological effects on the lung and that these effects may in part be mediated by the PG/VG base. However, it should be noted that this study, as also stated by the authors, and many others have one major limitation regarding the fact that the vapers included in the studies were mostly ex-smokers and therefore results should be interpreted in the context of this background. Similarly, concerning the recent outbreak of electronic-cigarette, or vaping, product use–associated lung injury (EVALI) it should be noted that this is still a matter of debate and in most of the cases it was related to the use of non-commercial additive or improper use of the devices.

Also, an important milestone was also the establishment of collaboration between the scientists that will expand teamwork and progress knowledge in the field of reduced risk non-combustible tobacco and nicotine products. Overall, this study confirmed that most of the harm to bronchial epithelial cells arose from volatile compounds in cigarette smoke rather than TPM or nicotine and demonstrated that ENDS are significantly less toxic compared to cigarettes.

## Materials and methods

### Recruitment of laboratories

International Laboratories involved in cytotoxicity studies were invited to participate in the inter-laboratory Replica study based on predefined criteria. An online questionnaire was administered to the international laboratories participating to the study that listed skills and knowledge pertaining to the core activities of in vitro research to allow us to assess levels of proficiency in general and in relation to specific areas of our research, including experience in biological assessments of cytotoxicity and ELISA determination. A section of the questionnaire outlined required equipment and laboratory compliance with the *Routine Analytical Cigarette-Smoking Machine—Definitions and Standard Conditions* ISO3308:2012 (International Organization for Standardization 2018), European Good Laboratory Practice, and US Environmental Protection Agency Good Laboratory Practice Standards guidelines.

The selected laboratories were provided with workshops, hands-on training, and on-site assessments of laboratory capacity and personnel expertise, with follow-up by virtual sessions if necessary. Although scientists had varying levels of experience in in vitro testing, most had not received previous formal training in smoke and aerosol exposure procedures. This was provided along with the standard operating procedures (SOPs) for use of smoking/vaping machines and cell-exposure systems. Four selected laboratories in academic establishments and one from the private sector joined this study: one from each of Italy (LAB-A; leading center), Greece (LAB-B), Oman (LAB-C), USA (LAB-D), and Serbia (LAB-E).

### Harmonization process

Laboratory protocols were harmonized across study sites with SOPs defined for each experimental step and use of the same cell lines, cell-exposure equipment, and methods to assess endpoints, as suggested by the Center for Open Science transparency and openness promotion guidelines (https://www.cos.io/initiatives/top-guidelines). A 4-day kick-off meeting was held by LAB-A to introduce the SOPs and provide personnel training. The SOPs for cell exposure to cigarette smoke and ENDS aerosol, cell culture, cytotoxicity assessment using the neutral red uptake (NRU) assay, and ELISA cytokine determination were adopted from the original studies^4,10,15^ and manufacturers’ instructions and adapted by the five principal investigators according to laboratory capacity, equipment, and test products, ensuring they met the ISO3308:2012 guidelines (International Organization for Standardization 2018).

Datasheets for detailed recording of technical data related to critical protocol steps and deviation communication forms were prepared by LAB-A and shared with the laboratory partners. Microsoft Excel (version 16.43, 2011, Microsoft, Redmond, WA, USA) template spreadsheets were prepared and distributed to the laboratories for data analysis and reporting. The data manager (RE) created a folder for collection of documents during the study, to which only she and respective laboratory had access.

After training and completion of equipment set-up at each center, the study principal investigator and co-principal investigator from LAB-A had planned to visit each participating laboratory to verify correct installation and functioning and compliance to standards and to conduct additional on-site training if required. Unfortunately, travel restrictions imposed due to the SARS-CoV-2 pandemic meant that it was not possible to carry out on-site training in LAB-D in the USA, and this was replaced by video conferences.

To maximize assay standardization of cell growth, cytotoxicity assessment, and cytokine determination, a list of key consumables was shared with all laboratories and these were obtained from the same lot when possible. We purchased American Type Culture Collection (Manassas, VA, USA) Human NCI-H292 [H292] (ATCC® CRL-1848™) bronchial epithelial cells (NCI-H292). A SOP was distributed for thawing, freezing, and subculturing of the cell line, including testing for mycoplasma contamination with the Plasmotest™ kit (InvivoGen, San Diego, CA, USA) before freezing the cells, to allow the laboratory partners to generate their own working cell bank.

### Original studies

Three studies were replicated. First, we assessed the methods of Azzopardi et al. to test inflammatory and cytotoxic responses to smoke in an air‒liquid interface cell culture model (experiment 1)^10^. We measured the inhibitory effects of cigarette smoke (whole smoke [WS] and vapor phase [VP]) on NCI-H292 cells under two different smoking regimens. Study two was another analysis by Azzopardi et al. of cytotoxicity on NCI-H292 cells after exposure to e-cigarette aerosol compared with cigarette smoke (experiment 2)^4^. Third, we replicated a study by Jaunky et al. in which in vitro cytotoxic assessment was performed after exposure of NCI-H292 cells to aerosol from two THPs compared with cigarette smoke (experiment 3)^15^. Results from experiments 2 and 3 were compared with those for cigarette WS under HCI smoking regimen based on the half-maximal inhibitory concentration (IC_50_) result from experiment 1. Concentration of the secreted inflammatory cytokines interleukin-6 and interleukin-8 (IL-6 and IL-8) and the tissue remodeling mediator matrix metalloproteinase 1 (MMP-1) were measured in experiment 1.

### Chemicals and reagents

Chemicals and reagents were obtained from the following sources: Dulbecco’s Modified Eagle Medium high glucose (DMEM-hg), RPMI-1640 medium (without glutamine), phosphate buffered saline (PBS), penicillin–streptomycin solution 10,000 U/mL, L-glutamine 200 mM, Transwell culture inserts (12 mm diameter, 0.4 μM pore size), trypsin–EDTA, IL-6 and IL-8 human Instant ELISA kits (catalog number BMS204-3INST), and MMP-1 human ELISA kit (catalog number EHMMP1) from ThermoFisher Scientific in various locations; glacial acetic acid, neutral red solution, formaldehyde solution, and absolute ethanol (≥ 99.8%) from Sigma–Aldrich in various locations; fetal bovine serum ‒ South America Origin from Corning (New York, NY, USA; LOT#35,079,016); and UltraCULTURE from Lonza (Basel, Switzerland).

### Cell culture

The cell culture methods have been described in the three original studies (Azzopardi et al., 2015, 2016; Jaunky et al., 2018). Briefly, NCI-H292 cells were cultured in RPMI 1640 medium (10% fetal bovine serum, 2 mM L-glutamine, 50 U/mL penicillin, and 50 mg/mL streptomycin) at 37 °C, in 5% CO_2_ and a humidified atmosphere. Cells were seeded 48 h before exposure in 12 mm Transwell inserts at a density of 3 × 10^5^ cells per mL, sustained by 1 mL RPMI medium in the basal compartment of each well and 0.5 mL in the apical compartment of each insert until they reach 80% confluency. Cells were starved for 24 h prior to exposure by replacing the basal and apical medium with 1 mL and 0.5 mL, respectively, of UltraCULTURE containing 2 mM glutamine, 50 U/mL penicillin, and 50 μg/mL streptomycin.

### Test products

In the original studies University of Kentucky 3R4F reference cigarettes were used to generate cigarette smoke. However, at the time of this study these cigarettes were no longer produced and were replaced with University of Kentucky 1R6F reference cigarettes, which have been validated as an appropriate replacement (Jaccard et al. 2019). As in the original experiments, 1R6F cigarettes were conditioned for a minimum of 48 h before use in 60% (± 3%) relative humidity and at 22 °C (± 1 °C) and smoked in a test atmosphere of 60% (± 5%) relative humidity at a temperature of 22 °C (± 2 °C), in accordance with ISO 3402:1999 (International Organization for Standardization 1999).

Two commercially available e-cigarettes, the Vype eStick and a Vype ePen 3 (Nicoventures, Blackburn, UK) were used in this study. Vype eStick is a puff-activated cigarette-like product that includes a battery unit with a capacity of 280 mAh and a replaceable pre-filled cartridge containing e-liquid and a cartomizer. The e-liquid contained the “Toasted Tobacco” flavor and 18 mg/mL nicotine. In the original study by Azzopardi et al. (2016), the first version of the Vype ePen was used but was no longer available. Instead, the Vype ePen 3 was used. This is a button-activated closed e-cigarette system consisting of two separate modules—a rechargeable battery section 650 mAh power with 6 W resistance (output 5.0 V) and a replaceable section containing a closed e-liquid cartridge and a cartomizer equipped with a cotton wick and coil heating system. We used the “Master Blend” flavor with 18 mg/mL nicotine.

The two THPs used by Jaunky et al. were the commercially available glo (referred to as THP1.0 by Jaunky et al.; British American Tobacco, Southampton, UK) and IQOS (named as THS in the paper by Jaunky et al.; Phillip Morris International, Neuchâtel, Switzerland). As the models used are no longer available at the time of this study, we used the glo PRO and IQOS 3 DUO. The tobacco consumables were, respectively, the Neostick “Ultramarine” and the Heets “Sienna selection” (Red). All devices and consumables for test products were sourced from authorized dealers in Italy, except for the Vype eStick, which was obtained from Greece.

### Smoke and aerosol generation and exposure parameters

**Figure 12:**
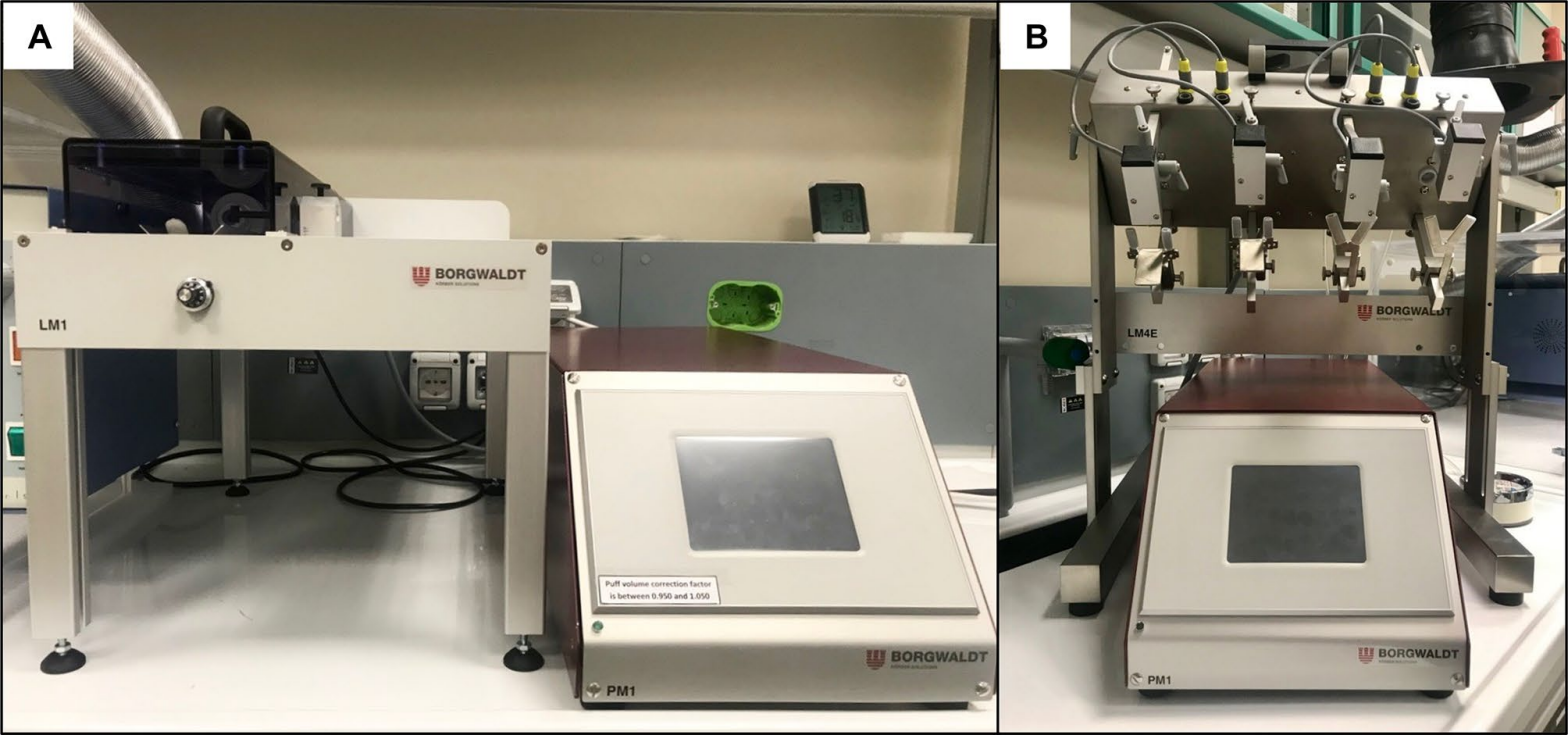
Borgwaldt LM1 smoking machine (**A**) and LM4E vaping machine (**B**). Photos edited by using GIMP image manipulation program (version 2.10.14).

**Figure 13:**
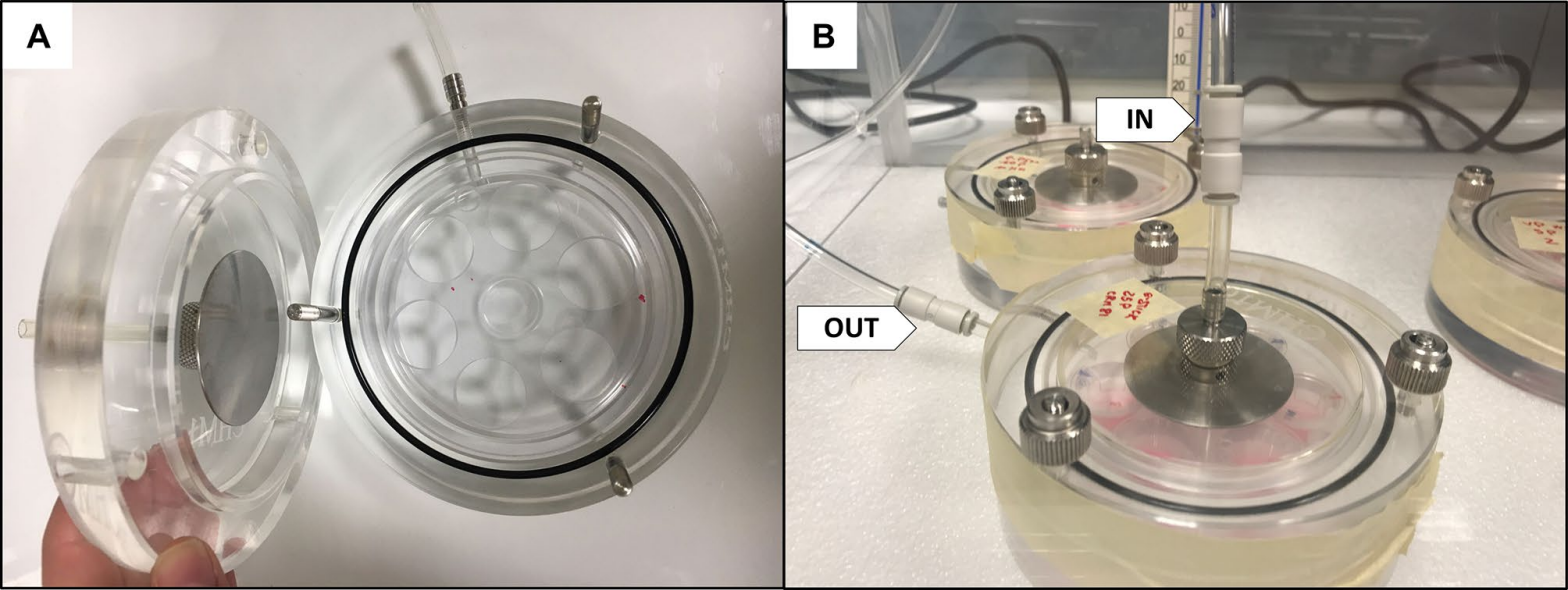
Perspex aerosol exposure chamber. Photos edited by using GIMP image manipulation program (version 2.10.14).

Azzopardi et al. (2015, 2016) used a Borgwaldt RM20S smoking machine (Hamburg, Germany) to generate cigarette smoke and adapted it to produce e-cigarette aerosol. This model allowed smoke and aerosol dilution ranging from 1:500 to 1:2.5 (smoke/aerosol:air, vol:vol). By the time of this study, separate machines had been developed for smoking and vaping. Therefore, we used a Borgwaldt LM1 smoking machine and a Borgwaldt LM4E Vaping Machine to generate respectively 1R6F cigarette smoke and ENDS aerosol (Fig. 12). Neither enables smoke/aerosol dilution and, therefore, all experiments in this study were performed with undiluted cigarette smoke and ENDS aerosol. The cell-exposure chambers used in this study were the same as those previously described by Azzopardi et al.^4,10^ and Jaunky et al.^15^ (Fig. 13).

In experiment 1, we exposed cells to 1R6F smoke (WS and VP) using the two regimens: the ISO 3308:2000 smoking regimen (puff volume, duration, and frequency of 35 mL, 2 s and 60 s, respectively, with a bell profile) or the Health Canada Intense regimen ([HCI] puff volume, duration, and frequency of 55 mL, 2 s and 30 s, respectively, with a bell profile). VP exposure was achieved by positioning a Cambridge filter pad in line, immediately after the cigarette.

In experiment 2, e-cigarettes were fully charged and loaded with fresh cartomizers for each exposure. Vype ePen 3 was vaped using a modified HCI regimen (puff volume, duration, and frequency of 55 mL, 2 s, 30 s, respectively, with a rectangular profile) with 1 s button pre-activation for each puff and held at a 45° angle (mouthpiece up) to reflect consumer use. Vype eStick was vaped using the CORESTA reference method no. 81 regimen (puff volume, duration, and frequency of 55 mL, 3 s, 30 s, respectively, with a rectangular profile) (CORESTA 2015). The regimens have been chosen as per Azzopardi et al. (2016).

In experiment 3, THPs were manually button-activated to initiate device heating prior to syringe activation. They were fully charged, cleaned, and loaded with fresh tobacco consumables for each exposure. The glo PRO was activated 40 s prior to puffing. The IQOS 3 DUO was activated 30 s before puffing. Heat cycles used were those mandated by manufacturer’s product design specification for each THP. Both THPs were puffed following the HCI regimen but with filter vents unblocked to avoid device overheating.

### Exposure of cells

Prepared cells in the inserts were transitioned to the air‒liquid interface (ALI) by removal of the apical culture medium. Two inserts per test product were transferred to the Perspex aerosol exposure chamber (Fig. 13), with 25 mL DMEM-hg, 50 U/mL penicillin, and 50 mg/mL streptomycin in the basal compartment of the chamber to perform the ALI exposure.

Two negative controls cell insert were created, consisting of one seeded insert with apical media (incubator control—INC) and one without apical media (ALI control). A positive control was also created with 1 mL apical and 2 mL basal DMEM-hg and 350 μM sodium dodecyl sulphate. These three controls were maintained at 37 °C and in 5% CO_2_ in the incubator throughout the duration of the experiments. For each experiment two negative controls exposed to laboratory filtered air by ALI (AIR control) were created using a dedicated port of the LM4E.

For experiment 1, NCI-H292 cells were exposed to two, five, 10, 12, 15, 20, 25, or 30 puffs of cigarette WS or cigarette VP under the ISO regimen and two, four, five, six, eight, 10, 15, or 20 puffs under the HCI regimen using the LM1 smoking machine. The nicotine concentration released at the IC_50_ dose of WS generated under the HCI regimen was determined in the media from the basal compartment of the exposure chamber and was used to set the puff numbers for the e-cigarettes and THPs for experiments 2 and 3 to ensure matched nicotine delivery to the exposure chamber media.

For experiment 2, NCI-H292 cells were exposed to 10 puffs from Vype ePen 3 (by modified HCI regimen) and 25 puffs from Vype eStick (by the CORESTA reference method no. 81 regimen).

For experiment 3, NCI-H292 cells were exposed to 8 puffs from Neostick (glo PRO) and to 7 puffs from Heets (IQOS 3 DUO) by the HCI regimen.

During exposure, cells were maintained at 37 °C in a thermostatic incubator.

Following exposure in all experiments, the culture inserts were transferred to fresh 12-well culture plates containing 1 mL supplemented UltraCULTURE pre-warmed to 37 °C. A further 0.5 mL supplemented UltraCULTURE was added to the apical surface of each culture insert and the cells were incubated for a recovery period of 24 h at 37 °C, in 5% CO_2_ and a humidified atmosphere.

### Cytotoxicity testing

As per the replicated studies, the NRU cytotoxicity assay was used as a benchmark assay to evaluate cytotoxic effects in all experiments. After 24 h recovery period, UltraCULTURE from the apical and basal compartments of each culture insert was individually pooled and stored at ‒80 °C until the secreted inflammatory and tissue remodeling mediators were measured. The cells were washed twice with PBS then incubated with neutral red solution (0.05 g/L in UltraCULTURE) for 3 h at 37 °C in 5% CO_2_ and a humidified atmosphere. After incubation, cells were washed with PBS to remove unincorporated dye. The incorporated solution was eluted from the cells by adding 500 μL de-staining solution (50% ethanol, 49% distilled water, 1% glacial acetic acid, v:v:v) to each insert, followed by incubation for 10 min at 300 rpm on a plate shaker. Extracts were transferred to a 96-well plate in duplicate (100 μL per well) and optical density of NR extracts was read with a microplate spectrophotometer at 540 nm using a reference filter of 630 nm. Blank inserts (without cells) were used to assess how much neutral red solution stained the Transwell membranes and these background values were subtracted from each measurement.

For comparison purposes, cytotoxicity curves were expressed either as a function of number of puffs or of nicotine released in the basal media of the exposure chambers. Each laboratory performed one exposure of two different Transwell inserts at the same time (ten independent replicates) for 1R6F and ENDS.

### Inflammatory and tissue remodeling mediator secretion

ELISA kits were used to measure concentrations of IL-6 and IL-8 and MMP-1 24 h after exposure to cigarette WS and VP under the ISO and HCI regimens from experiment 1. The assays were performed according to the manufacturer protocols. Absorbance was measured at a wavelength of 450 nm and biomarker concentrations were calculated from a standard curve generated with purified proteins. The detection limits, as specified by the manufacturer, were 0.92 pg/mL for IL-6, 1.3 pg/mL for IL-8, and 8.0 pg/mL for MMP-1. Each measurement was performed in duplicate.

### Nicotine and total particulate matter dosimetry

As LAB-A was the only laboratory that could perform ultraperformance liquid chromatography-mass spectrometry, nicotine concentrations were quantified in the cell culture media after each exposure there and provided as reference values for the other laboratories. Nicotine dosimetry was performed on 0.1 mL aliquots of media retrieved from the exposure chambers. Calibration standards of 1, 2, 5, 10, 20, or 50 µg/mL were added to each sample, with 100 µg/mL nicotine-(methyl-d3) solution used as an internal standard. Samples were vortexed and centrifuged at 2500*g* for 4 min before 0.1 mL sulfuric acid 0.1 M and 0.3 mL acetonitrile were added to each. Supernatants were transferred to vials with 250 μL conical inserts. Nicotine concentration was determined by ultraperformance liquid chromatography–tandem mass spectrometry and triple quadrupole mass spectrometry (Waters ACQUITY, Waters, Milford, MA, USA), operating in multiple reaction monitoring and positive ion mode (see Table S1 in supplementary information). A Waters ACQUITY UPLC HSS T3 1.8 μm, 2.1 × 100 mm column was used. Isocratic elution (80% water and 20% acetonitrile, both added at 0.1% with formic acid) was performed. The mass spectrometry settings were as follows: capillary energy 3.0 kV, source temperature 150 °C, column temperature 40 °C, desolvation temperature 500 °C, desolvation gas 1000 L/h, and cone gas 100 L/h.

Total particulate matter (TPM) was quantified gravimetrically by LAB-A, LAB-C, and LAB-D (LAB-B and LAB-E did not collect TPM weight). To determine TPM dose, Cambridge filter pads were weighed before and after exposure.

### Statistical analysis

All raw data were tabulated and processed in Microsoft Excel. The Interlaboratory Studies (ILS) package for R was used to assess consistency of results across the partner laboratories in terms of deviations from repeatability within laboratories (Sr), means values (SB) and reproducibility (SR) between laboratories.

Linear regression analysis was performed to assess the relationship of NRU cell viability data (expressed as percentages of air control) provided from each center. Bland‒Altman plots were calculated to describe the level of agreement between centers. GraphPad Prism 8 software was used to determine the IC_50_ values for each exposure (WS or VP under the ISO or HCI regimen) by fitting a sigmoidal dose–response curve with a variable slope to determine the best-fit values in an eight-parameter non-linear regression model, and to make comparisons between slopes. Moreover, linear regression analyses were performed to evaluate the best-fit slope between puff numbers and nicotine concentration or TPM weight followed by comparisons between ISO and HCI slopes. Data distribution was assessed with the Shapiro–Wilk test.

Inflammatory mediator data were checked by excluding the values beyond the minimum and maximum values from ELISA standard curves. Outlier values were detected by the Grubbs test. The data were compared by ANOVA with post-hoc Tukey adjustment.

Comparison of NRU cell viability after exposure to 1R6F WS, IQOS, glo, ePen and eStick aerosol was performed by using Kruskal–Wallis test followed by Wilcoxon multiple-comparison analysis with Holm’s correction. All analyses with *p* values < 0.05 were considered to be significant. R version 3.4.3 (2017-11-30) was used for data analysis and generation of graphs unless otherwise stated.

### Ethics approval

All experiments were performed in accordance with relevant guidelines and regulations. No animals or human tissue samples were used for the experiments. Cells from American Type Culture Collection (Manassas, VA, USA) were used for all the experiments in the manuscript: Human NCI-H292 [H292] (ATCC® CRL-1848™).

## Supplementary Information

Supplementary Information.

## Data Availability

The datasets generated during and/or analysed during the current study are available from the corresponding author on reasonable request.
