# Supplementary material for: Electronic nicotine delivery systems exhibit reduced bronchial epithelial cells toxicity compared to cigarette: the Replica Project
Source: Sci Rep. 2021 Dec 17;11:24182. doi: 10.1038/s41598-021-03310-y (PMC8683499; doi:10.1038/s41598-021-03310-y)
Supplement: Supplementary file 1 — Supplementary Information. [file 41598_2021_3310_MOESM1_ESM.pdf]

Electronic nicotine delivery systems exhibit reduced bronchial epithelial cells toxicity compared to cigarette: The Replica Project

Massimo Caruso[+1, 2], Rosalia Emma[+1,2], Alfio Distefano[1], Sonja Rust[2], Konstantinos Poulas[3,4], Fahad Zadjali[5], Antonio Giordano[6], Vladislav Volarevic[7], Konstantinos Mesiakaris[3,4], Mohammed Al Tobi[5], Silvia Boffo[6], Aleksandar Arsenijevic[7], Pietro Zuccarello[8], Cesarina Giallongo[8], Margherita Ferrante[8], Riccardo Polosa[2,9], Giovanni Li Volti*[1,2] and the *Replica Project Group*[#].

[1] Department of Biomedical and Biotechnological Sciences, University of Catania, Via S. Sofia, 97, 95123 Catania (Italy); mascaru@unict.it; rosalia.emma@unict.it; distalfio@gmail.com; livolti@unict.it
[2] Center of Excellence for the Acceleration of Harm Reduction (CoEHAR), University of Catania, Via S. Sofia, 97, 95123, Catania (Italy); sonja.rust@eclatrbc.it;
[3] Institute for Research and Innovation, IRIS, Patras Science Park, Patras (Greece); pha2823@upnet.gr
[4] Laboratory of Molecular Biology and Immunology, Department of Pharmacy, University of Patras, Patras (Greece); kpoulas@upatras.gr
[5] College of Medicine and Health Sciences, Department of Clinical Biochemistry, Sultan Qaboos University. P.O. Box 35, P.C 123, Khodh, Oman. fahadz@squ.edu.om; toubim@squ.edu.om
[6] Sbarro Institute for Cancer Research and Molecular Medicine, Department of Biology, College of Science and Technology, Temple University, Philadelphia (USA); giordano@temple.edu; silvia.boffo@temple.edu
[7] Center for Molecular Medicine and Stem Cell Research, Department of Microbiology and Immunology, Faculty of Medical Sciences, University of Kragujevac, Serbia, 69 Svetozara Markovica Street, 34000 Kragujevac (Serbia); vladislav.volarevic@medf.kg.ac.rs; aleksandar@medf.kg.ac.rs
[8] Department of Medical, Surgical Sciences and Advanced Technologies "G.F. Ingrassia", University of Catania, Via S. Sofia, 87, 95123 Catania (Italy); pietro.zuccarello@unict.it; cesarina.giallongo@unict.it; marfer@unict.it
[9] Department of Clinical and Experimental Medicine, University of Catania, Via S. Sofia, 97, 95123, Catania (Italy); polosa@unict.it
[+]these authors contributed equally to this work

*Corresponding author
Giovanni Li Volti
Department of Biomedical and Biotechnological Sciences
University of Catania
Via S. Sofia, 97, 95123 Catania, Italy
livolti@unict.it
Tel.: +39 095 4781239

Supplementary Material

Nicotine dosimetry

Table S1. MRM transitions monitored (m/z) with cone and collision voltages

Analyte	MRM (*m*/*z*)	Cone (volts)	Collision energy (eV)
Nicotine	163 → 117	40	25
	163 → 132	40	15
Nicotine-(methyl-d3)	165.8 → 116.8	40	20
	165.8 → 129.7	40	20

Abbreviation: MRM, multiple reaction monitoring.

Intra- and Inter-laboratory variability evaluation of 1R6F dose response curves, by exposure condition (Experiment 1)

ISO Whole Smoke (WS) exposure

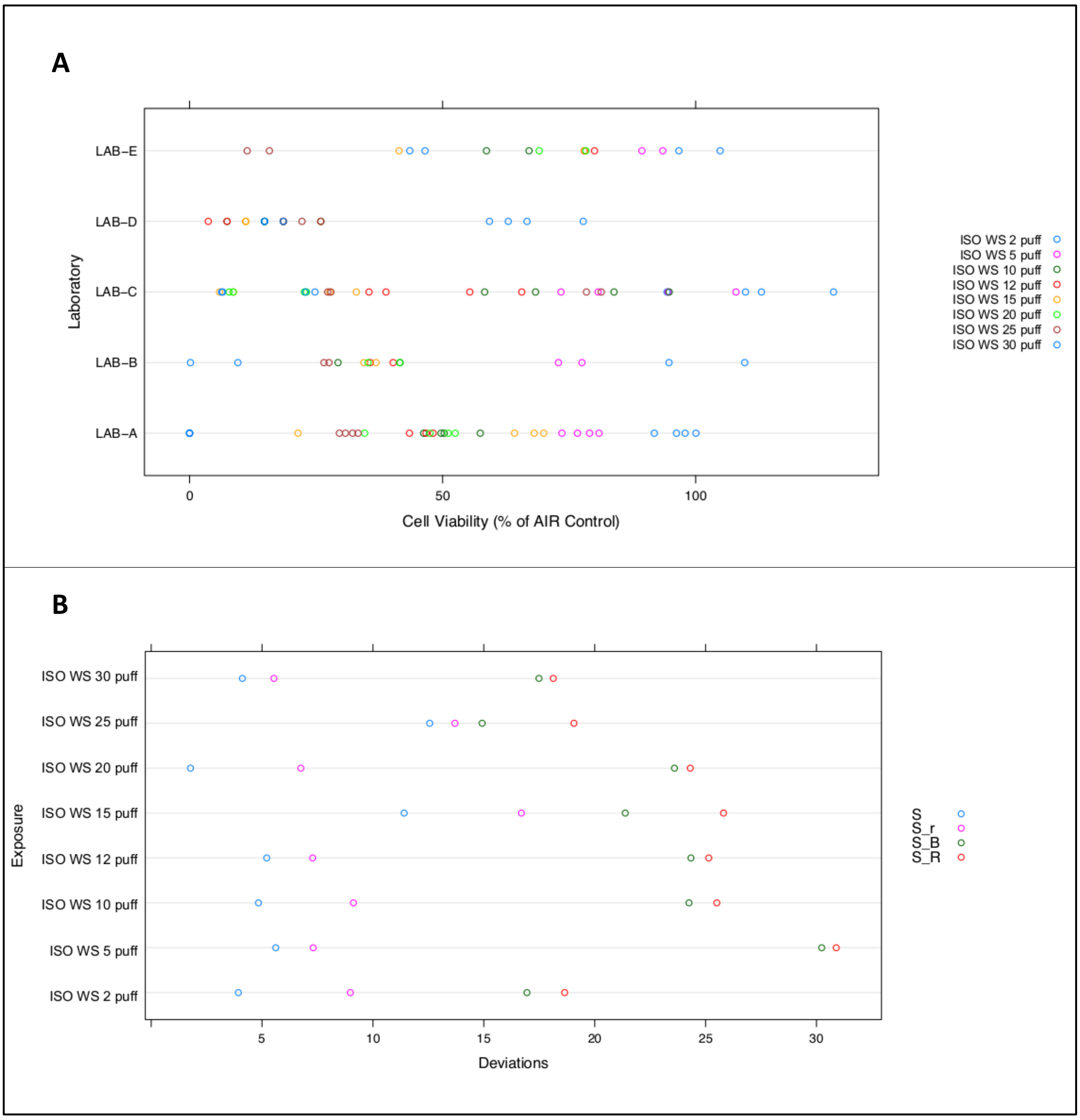

Figure S1. Laboratory performance calculated from 1R6F dose-response curves under ISO whole smoke exposure. (**A**) NRU cell viability and (**B**) variability by number of puffs after exposure to ISO 1R6F whole smoke. Abbreviations: ISO, International Organization for Standardization regimen; S, global deviation of all laboratories; SB, interlaboratory deviation between the means; Sr, intra-laboratory deviation from repeatability; SR, interlaboratory deviation from reproducibility; WS, whole smoke. Graphs were generated with R version 3.4.3 (2017-11-30) and edited by using GIMP image manipulation program (version 2.10.14).

ISO Vapor Phase (VP) exposure

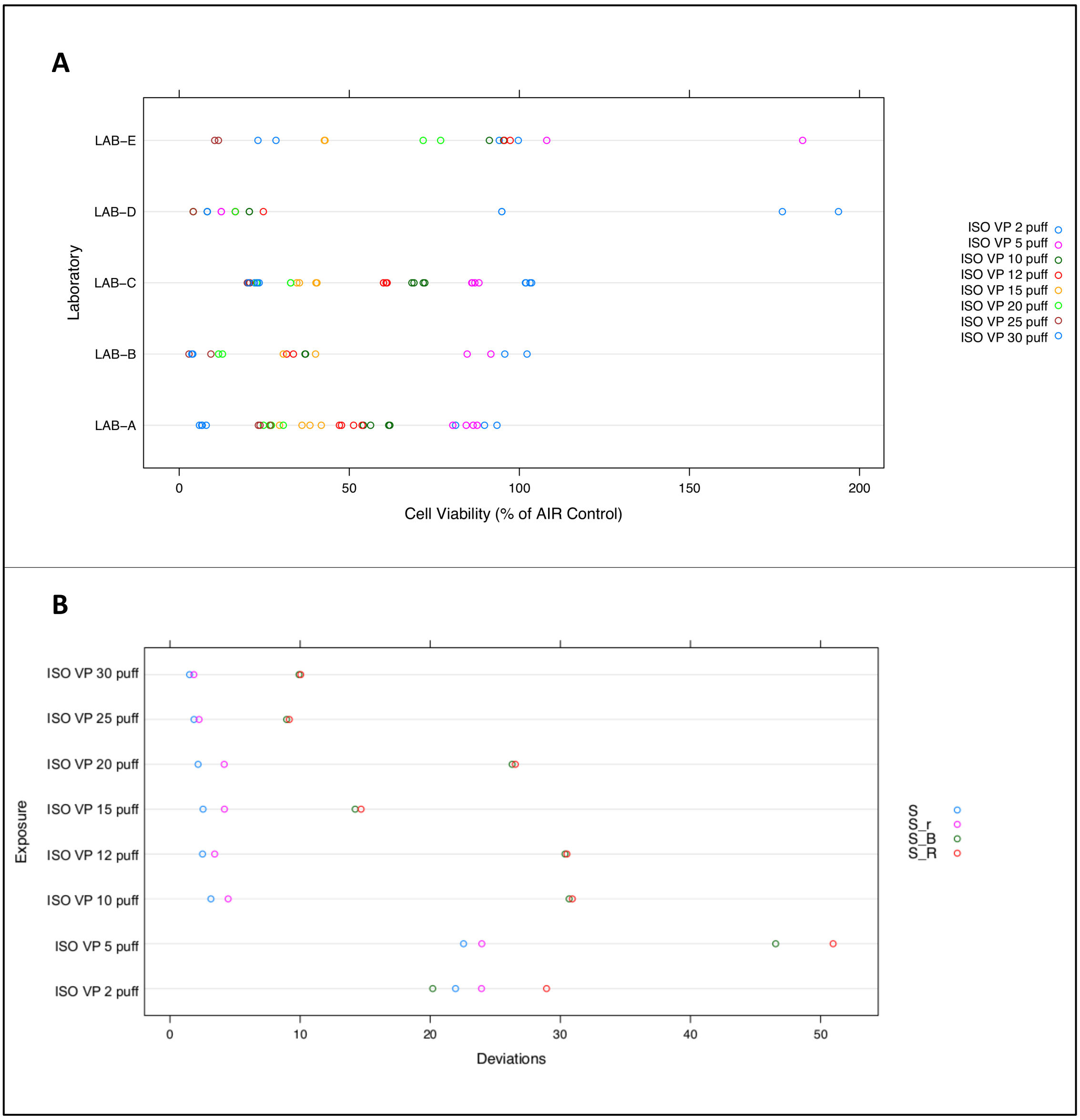

Figure S2. Laboratory performance calculated from 1R6F dose-response curves under ISO vapor phase exposure. (**A**) NRU cell viability and (**B**) variability by number of puffs after exposure to ISO 1R6F vapor phase. Abbreviations: ISO, International Organization for Standardization regimen; S, global deviation of all laboratories; SB, interlaboratory deviation between the means; Sr, intra-laboratory deviation from repeatability; SR, interlaboratory deviation from reproducibility; VP, vapor phase. Graphs were generated with R version 3.4.3 (2017-11-30) and edited by using GIMP image manipulation program (version 2.10.14).

HCI Whole Smoke (WS) exposure

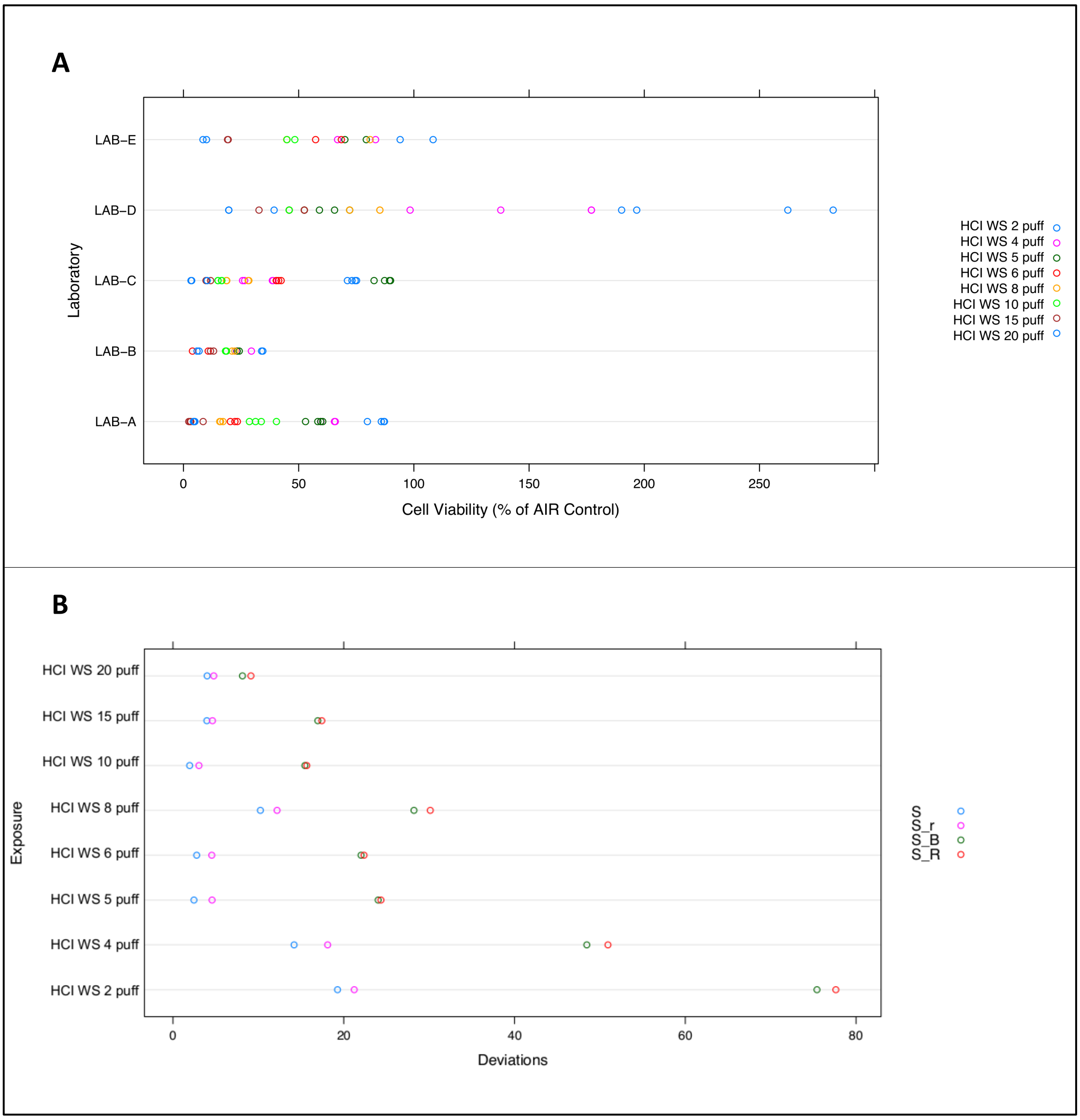

Figure S3. Laboratory performance calculated from 1R6F dose-response curves under HCI whole smoke exposure. (**A**) NRU cell viability and (**B**) variability by number of puffs after exposure to HCI 1R6F whole smoke. Abbreviations: HCI, Health Canada intense regimen; S, global deviation of all laboratories; SB, interlaboratory deviation between the means; Sr, intra-laboratory deviation from repeatability; SR, interlaboratory deviation from reproducibility; WS, whole smoke. Graphs were generated with R version 3.4.3 (2017-11-30) and edited by using GIMP image manipulation program (version 2.10.14).

HCI Vapor Phase (VP) exposure

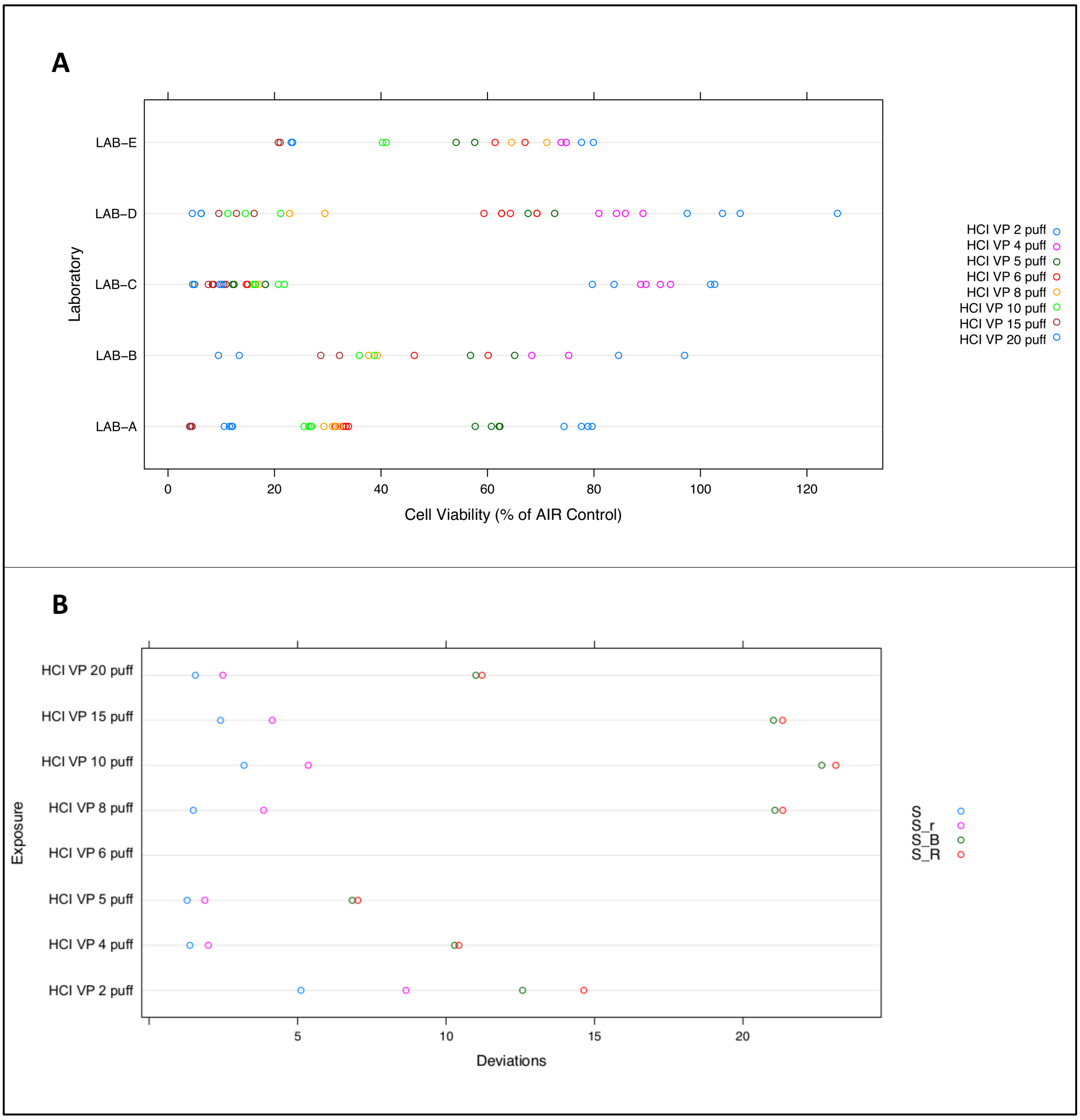

Figure S4. Laboratory performance calculated from 1R6F dose-response curves under HCI vapor phase exposure. (**A**) NRU cell viability and (**B**) variability by number of puffs after exposure to ISO 1R6F vapor phase. Abbreviations: HCI, Health Canada intense regimen; S, global deviation of all laboratories; SB, interlaboratory deviation between the means; Sr, intra-laboratory deviation from repeatability; SR, interlaboratory deviation from reproducibility; VP, vapor phase. Graphs were generated with R version 3.4.3 (2017-11-30) and edited by using GIMP image manipulation program (version 2.10.14).

Linear regression analyses and Bland-Altman plots for the evaluation of laboratory performances for 1R6F dose-response curves (Experiment 1).

ISO Whole Smoke (WS) exposure

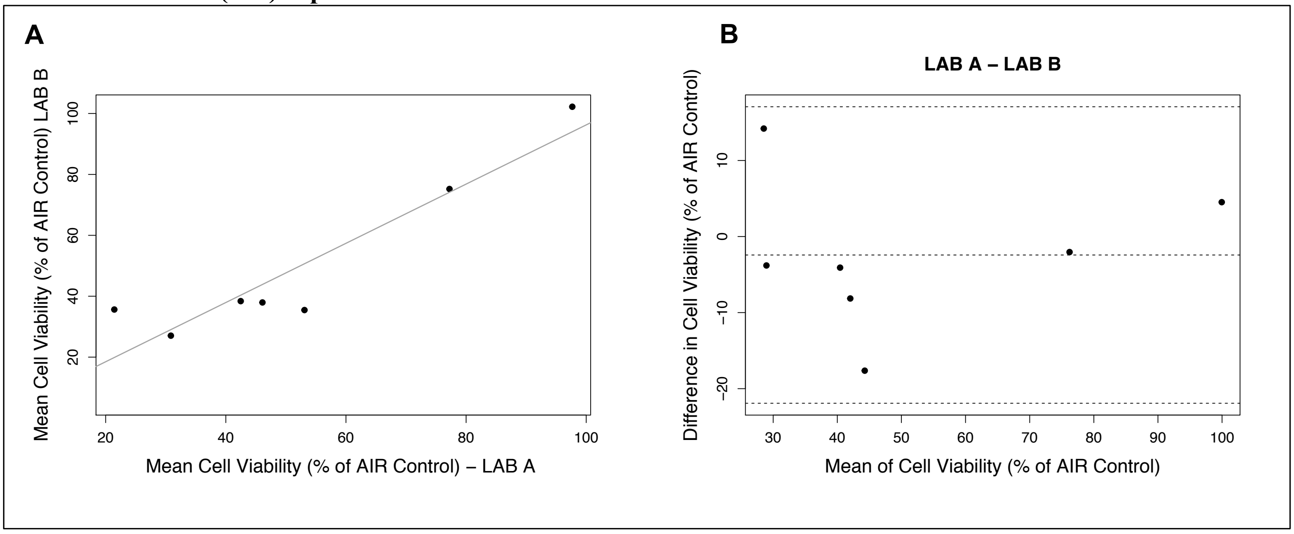

Figure S5. Assessment of reproducibility for NRU cell viability results after exposure to 1R6F ISO WS with different puff numbers. The left panel (**A**) shows the scatter plot of regression analysis of the Cell Viability Mean (% of AIR Control) between LAB-A and LAB-B (R= 0.871; p= 0.002). The right panel (**B**) shows the difference between the measurements obtained by LAB-A and LAB-B with respect to the mean in ISO WS each exposure condition (i.e. puff number) in the Bland Altman plot. No results had the Cell Viability (% of AIR Control) difference between LAB-A and LAB-B outside the 95% confidence interval. Graphs were generated with R version 3.4.3 (2017-11-30) and edited by using GIMP image manipulation program (version 2.10.14).

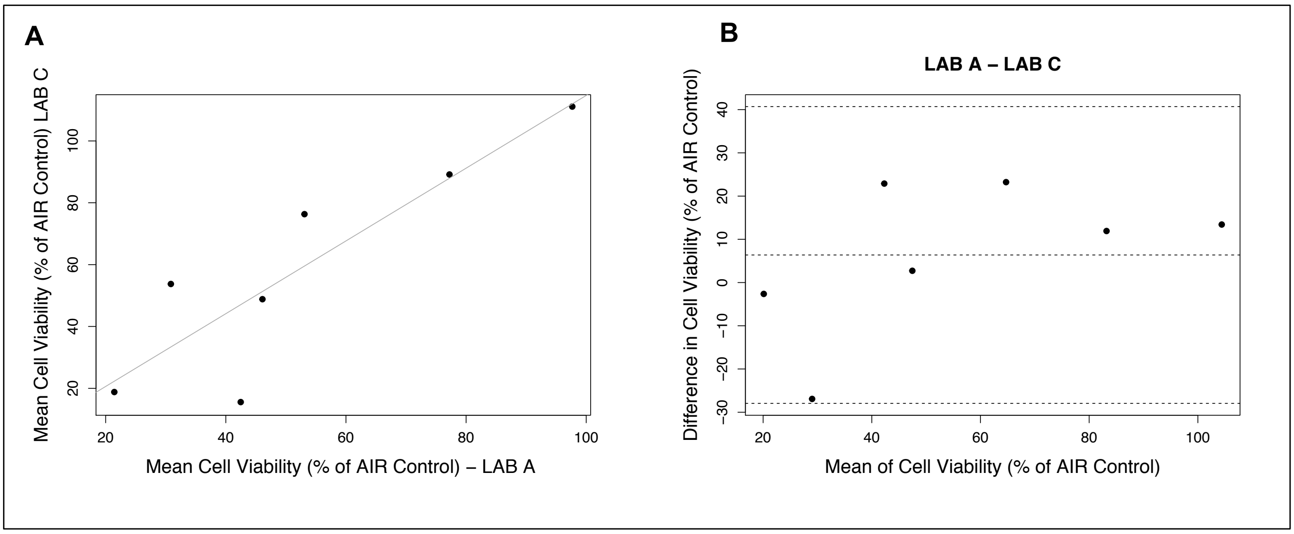

Figure S6. Assessment of reproducibility for NRU cell viability results after exposure to 1R6F ISO WS with different puff numbers. The left panel (**A**) shows the scatter plot of regression analysis of the Cell Viability Mean (% of AIR Control) between LAB-A and LAB-C (R= 0.774; p= 0.009). The right panel (**B**) shows the difference between the measurements obtained by LAB-A and LAB-C with respect to the mean in each ISO WS exposure condition (i.e. puff number) in the Bland Altman plot. No results had the Cell Viability (% of AIR Control) difference between LAB-A and LAB-C outside the 95% confidence interval. Graphs were generated with R version 3.4.3 (2017-11-30) and edited by using GIMP image manipulation program (version 2.10.14).

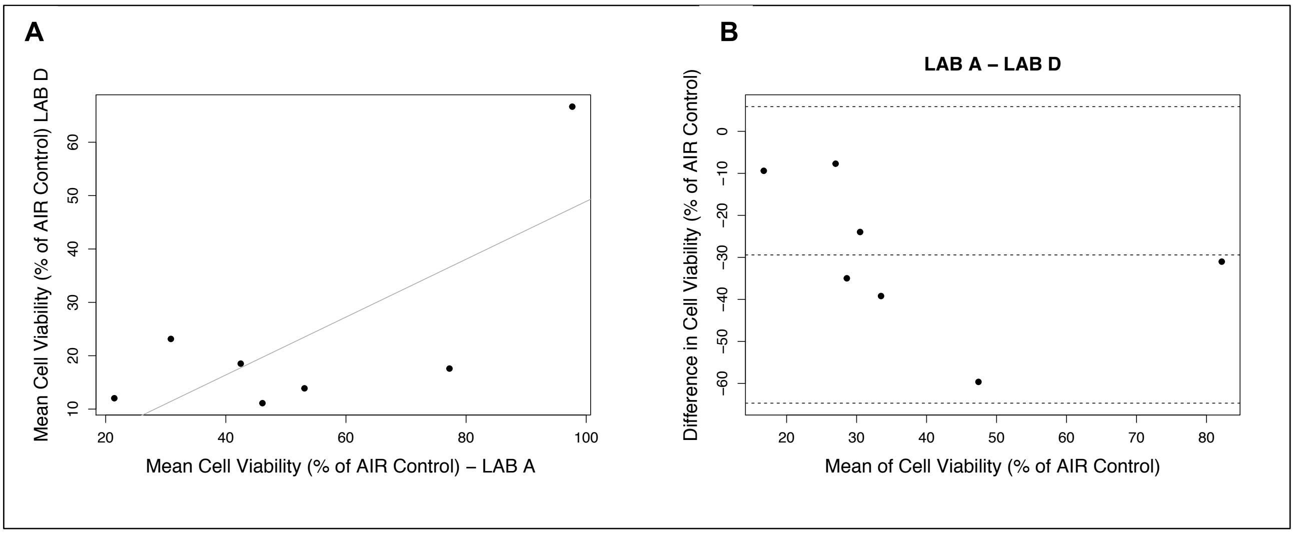

Figure S7. Assessment of reproducibility for NRU cell viability results after exposure to 1R6F ISO WS with different puff numbers. The left panel (**A**) shows the scatter plot of regression analysis of the Cell Viability Mean (% of AIR Control) between LAB-A and LAB-D (R= 0.54; p= 0.059). The right panel (**B**) shows the difference between the measurements obtained by LAB-A and LAB-D with respect to the mean in each ISO WS exposure condition (i.e. puff number) in the Bland Altman plot. No results had the Cell Viability (% of AIR Control) difference between LAB-A and LAB-D outside the 95% confidence interval. Graphs were generated with R version 3.4.3 (2017-11-30) and edited by using GIMP image manipulation program (version 2.10.14).

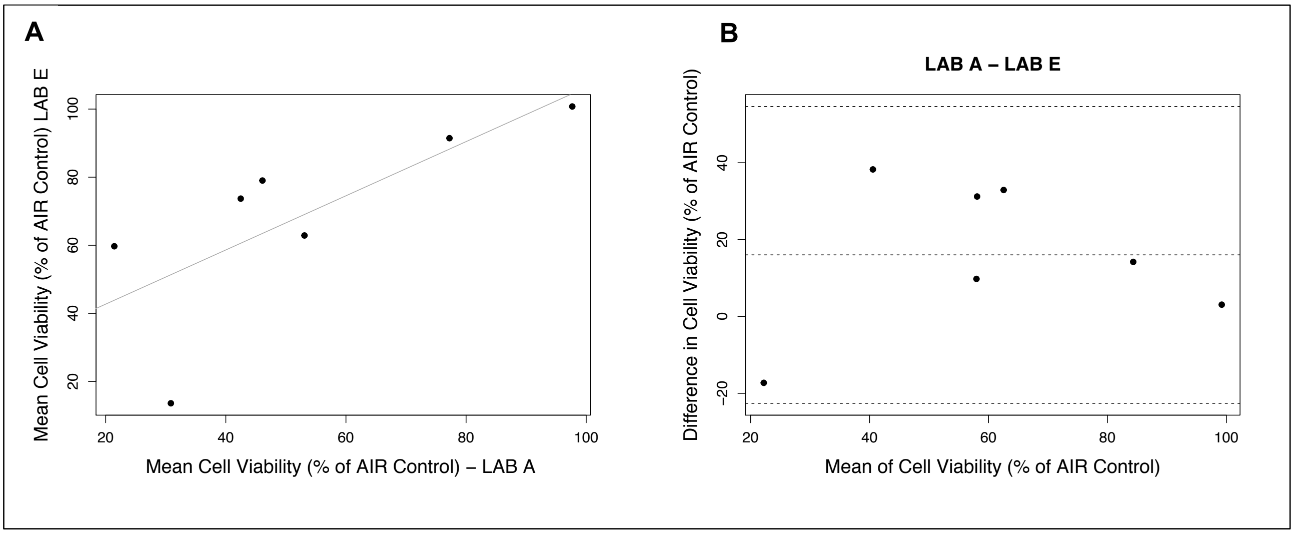

Figure S8. Assessment of reproducibility for NRU cell viability results after exposure to 1R6F ISO WS with different puff numbers. The left panel (**A**) shows the scatter plot of regression analysis of the Cell Viability Mean (% of AIR Control) between LAB-A and LAB-E (R= 0.554; p= 0.055). The right panel (**B**) shows the difference between the measurements obtained by LAB-A and LAB-E with respect to the mean in each ISO WS exposure condition (i.e. puff number) in the Bland Altman plot. No results had the Cell Viability (% of AIR Control) difference between LAB-A and LAB-E outside the 95% confidence interval. Graphs were generated with R version 3.4.3 (2017-11-30) and edited by using GIMP image manipulation program (version 2.10.14).

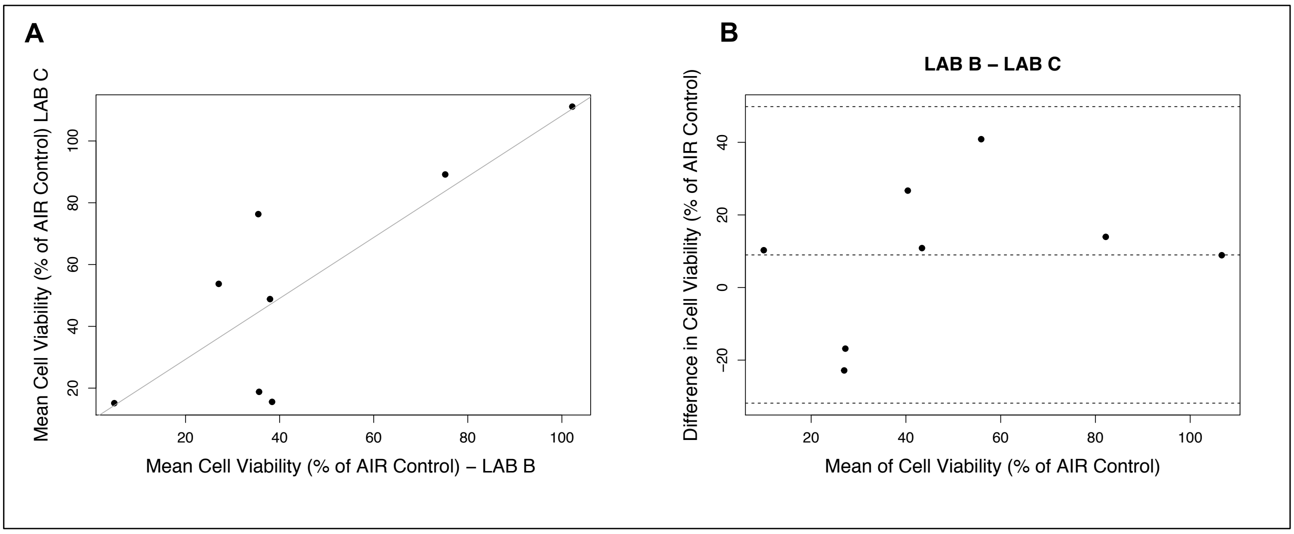

Figure S9. Assessment of reproducibility for NRU cell viability results after exposure to 1R6F ISO WS with different puff numbers. The left panel (**A**) shows the scatter plot of regression analysis of the Cell Viability Mean (% of AIR Control) between LAB-B and LAB-C (R= 0.67; p= 0.013). The right panel (**B**) shows the difference between the measurements obtained by LAB-B and LAB-C with respect to the mean in each ISO WS exposure condition (i.e. puff number) in the Bland Altman plot. No results had the Cell Viability (% of AIR Control) difference between LAB-B and LAB-C outside the 95% confidence interval. Graphs were generated with R version 3.4.3 (2017-11-30) and edited by using GIMP image manipulation program (version 2.10.14).

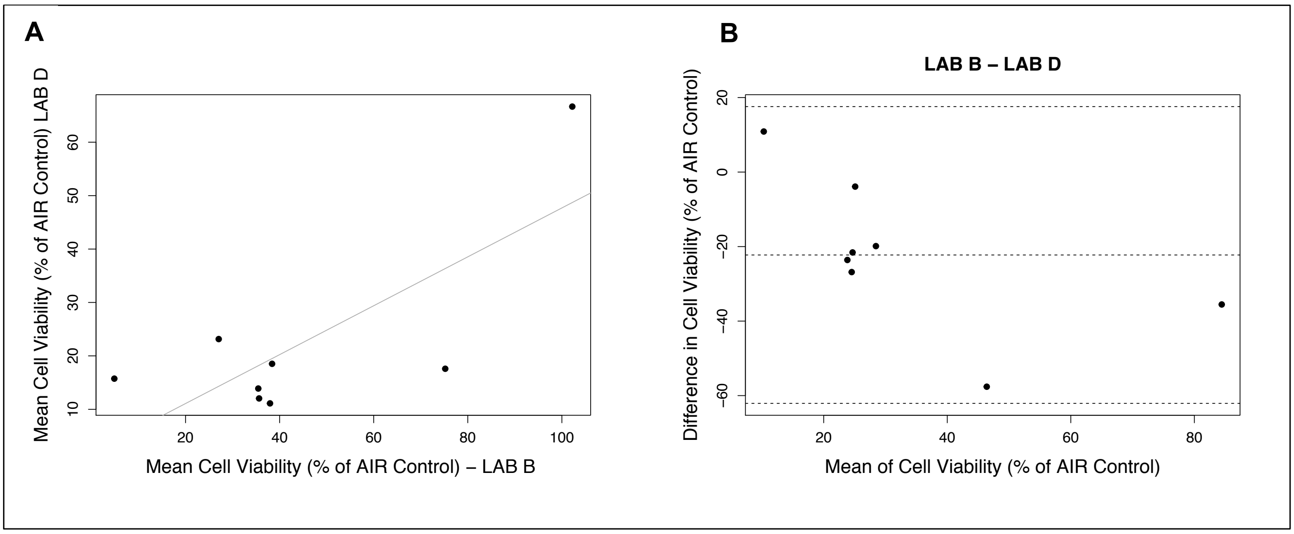

Figure S10. Assessment of reproducibility for NRU cell viability results after exposure to 1R6F ISO WS with different puff numbers. The left panel (**A**) shows the scatter plot of regression analysis of the Cell Viability Mean (% of AIR Control) between LAB-B and LAB-D (R= 0.57; p= 0.03). The right panel (**B**) shows the difference between the measurements obtained by LAB-B and LAB-D with respect to the mean in each ISO WS exposure condition (i.e. puff number) in the Bland Altman plot. No results had the Cell Viability (% of AIR Control) difference between LAB-B and LAB-D outside the 95% confidence interval. Graphs were generated with R version 3.4.3 (2017-11-30) and edited by using GIMP image manipulation program (version 2.10.14).

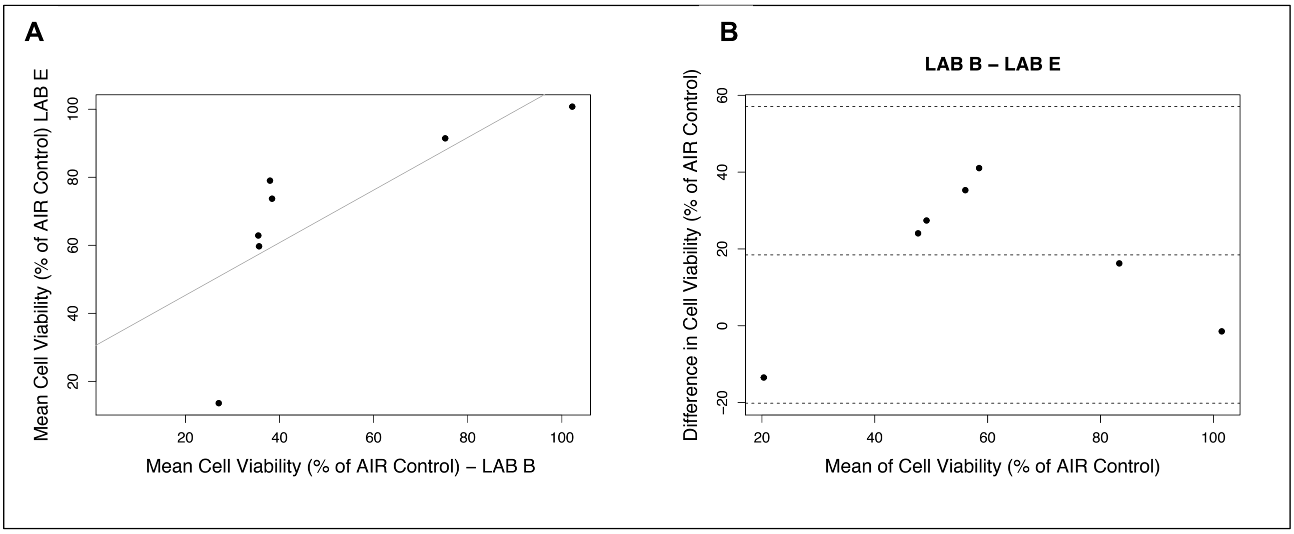

Figure S11. Assessment of reproducibility for NRU cell viability results after exposure to 1R6F ISO WS with different puff numbers. The left panel (**A**) shows the scatter plot of regression analysis of the Cell Viability Mean (% of AIR Control) between LAB-B and LAB-E (R= 0.57; p= 0.05). The right panel (**B**) shows the difference between the measurements obtained by LAB-B and LAB-E with respect to the mean in each ISO WS exposure condition (i.e. puff number) in the Bland Altman plot. No results had the Cell Viability (% of AIR Control) difference between LAB-E and LAB-D outside the 95% confidence interval. Graphs were generated with R version 3.4.3 (2017-11-30) and edited by using GIMP image manipulation program (version 2.10.14).

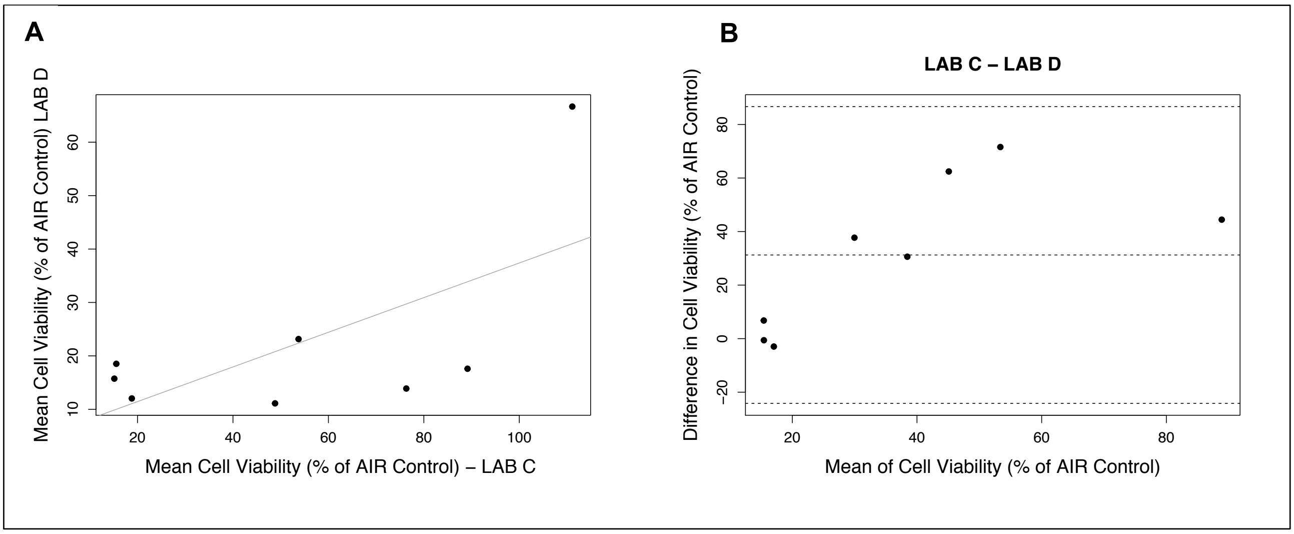

Figure S12. Assessment of reproducibility for NRU cell viability results after exposure to 1R6F ISO WS with different puff numbers. The left panel (**A**) shows the scatter plot of regression analysis of the Cell Viability Mean (% of AIR Control) between LAB-C and LAB-D (R= 0.41; p= 0.085). The right panel (**B**) shows the difference between the measurements obtained by LAB-C and LAB-D with respect to the mean in each ISO WS exposure condition (i.e. puff number) in the Bland Altman plot. No results had the Cell Viability (% of AIR Control) difference between LAB-C and LAB-D outside the 95% confidence interval. Graphs were generated with R version 3.4.3 (2017-11-30) and edited by using GIMP image manipulation program (version 2.10.14).

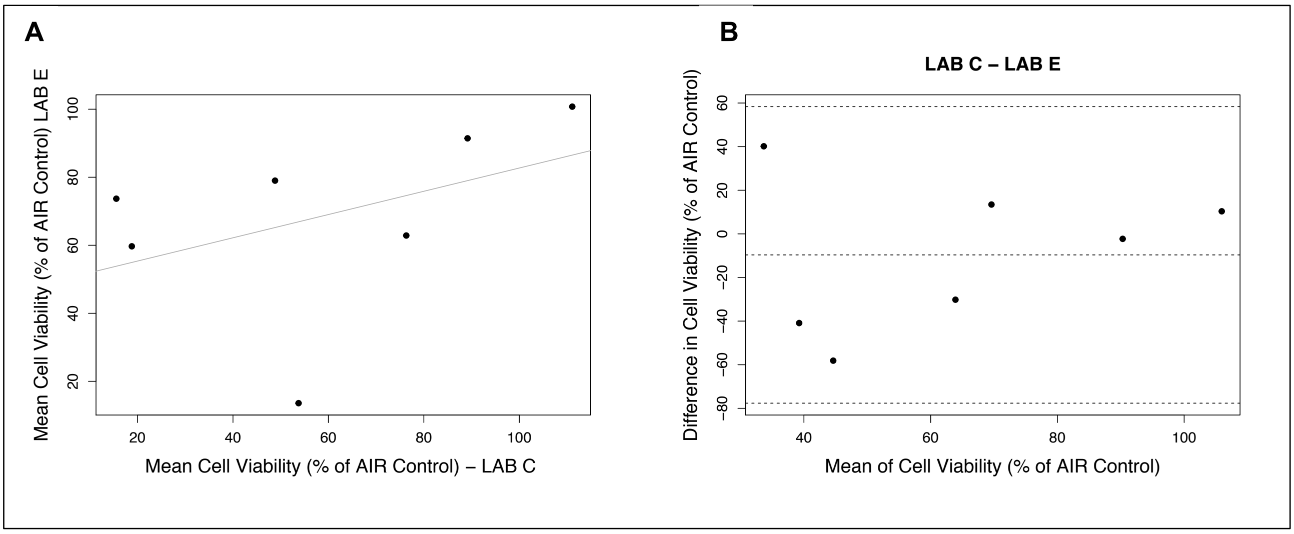

Figure S13. Assessment of reproducibility for NRU cell viability results after exposure to 1R6F ISO WS with different puff numbers. The left panel (**A**) shows the scatter plot of regression analysis of the Cell Viability Mean (% of AIR Control) between LAB-C and LAB-E (R= 0.18; p= 0.338). The right panel (**B**) shows the difference between the measurements obtained by LAB-C and LAB-E with respect to the mean in each ISO WS exposure condition (i.e. puff number) in the Bland Altman plot. No results had the Cell Viability (% of AIR Control) difference between LAB-C and LAB-E outside the 95% confidence interval. Graphs were generated with R version 3.4.3 (2017-11-30) and edited by using GIMP image manipulation program (version 2.10.14).

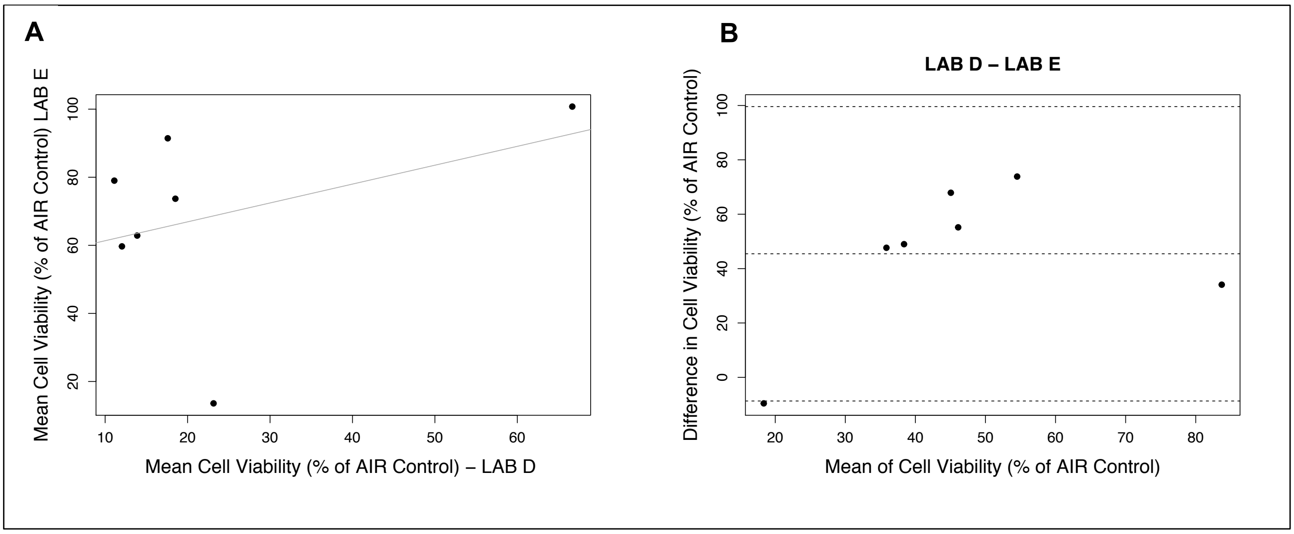

Figure S14. Assessment of reproducibility for NRU cell viability results after exposure to 1R6F ISO WS with different puff numbers. The left panel (**A**) shows the scatter plot of regression analysis of the Cell Viability Mean (% of AIR Control) between LAB-D and LAB-E (R= 0.18; p= 0.338). The right panel (**B**) shows the difference between the measurements obtained by LAB-D and LAB-E with respect to the mean in each ISO WS exposure condition (i.e. puff number) in the Bland Altman plot. One result had the Cell Viability (% of AIR Control) difference between LAB-D and LAB-E outside the 95% confidence interval. Graphs were generated with R version 3.4.3 (2017-11-30) and edited by using GIMP image manipulation program (version 2.10.14).

ISO Vapor Phase (VP) exposure

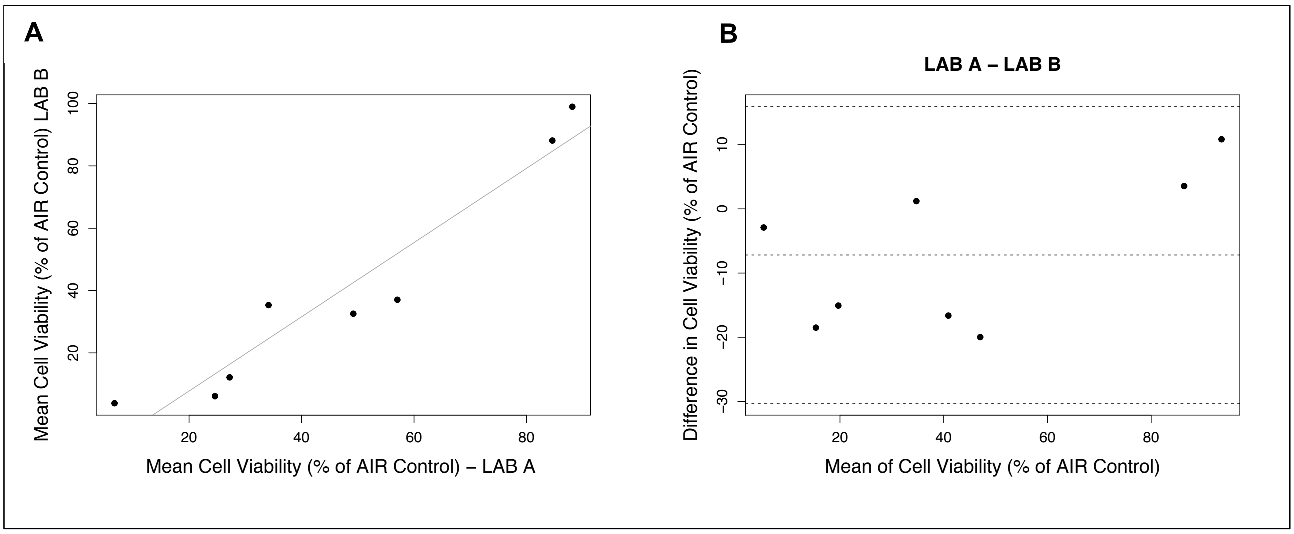

Figure S15. Assessment of reproducibility for NRU cell viability results after exposure to 1R6F ISO VP with different puff numbers. The left panel (**A**) shows the scatter plot of regression analysis of the Cell Viability Mean (% of AIR Control) between LAB-A and LAB-B (R= 0.917; p< 0.001). The right panel (**B**) shows the difference between the measurements obtained by LAB-A and LAB-B with respect to the mean in each ISO VP exposure condition (i.e. puff number) in the Bland Altman plot. No results had the Cell Viability (% of AIR Control) difference between LAB-A and LAB-B outside the 95% confidence interval. Graphs were generated with R version 3.4.3 (2017-11-30) and edited by using GIMP image manipulation program (version 2.10.14).

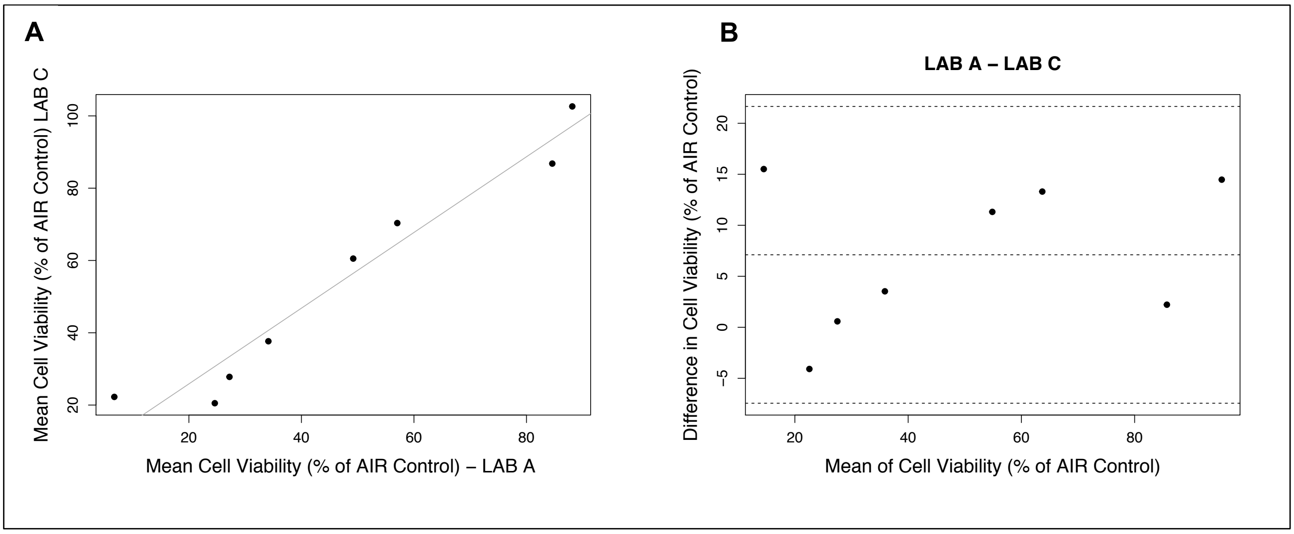

Figure S16. Assessment of reproducibility for NRU cell viability results after exposure to 1R6F ISO VP with different puff numbers. The left panel (**A**) shows the scatter plot of regression analysis of the Cell Viability Mean (% of AIR Control) between LAB-A and LAB-C (R= 0.946; p< 0.0001). The right panel (**B**) shows the difference between the measurements obtained by LAB-A and LAB-C with respect to the mean in each ISO VP exposure condition (i.e. puff number) in the Bland Altman plot. No results had the Cell Viability (% of AIR Control) difference between LAB-A and LAB-C outside the 95% confidence interval. Graphs were generated with R version 3.4.3 (2017-11-30) and edited by using GIMP image manipulation program (version 2.10.14).

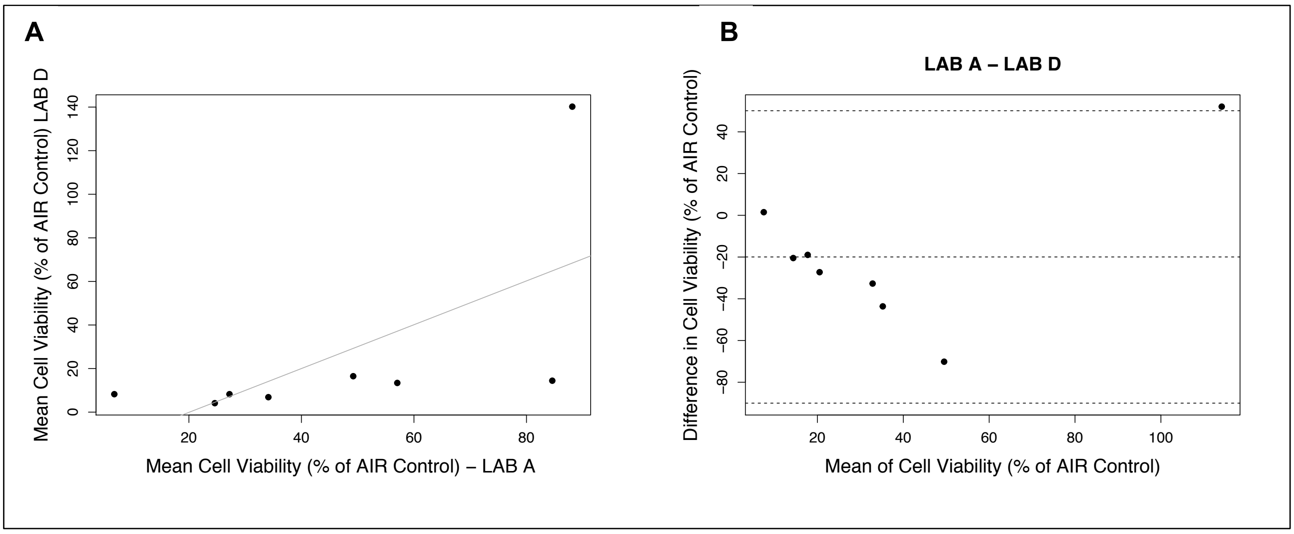

Figure S17. Assessment of reproducibility for NRU cell viability results after exposure to 1R6F ISO VP with different puff numbers. The left panel (**A**) shows the scatter plot of regression analysis of the Cell Viability Mean (% of AIR Control) between LAB-A and LAB-D (R= 0.4; p= 0.093). The right panel (**B**) shows the difference between the measurements obtained by LAB-A and LAB-D with respect to the mean in each ISO VP exposure condition (i.e. puff number) in the Bland Altman plot. One result had the Cell Viability (% of AIR Control) difference between LAB-A and LAB-D outside the 95% confidence interval. Graphs were generated with R version 3.4.3 (2017-11-30) and edited by using GIMP image manipulation program (version 2.10.14).

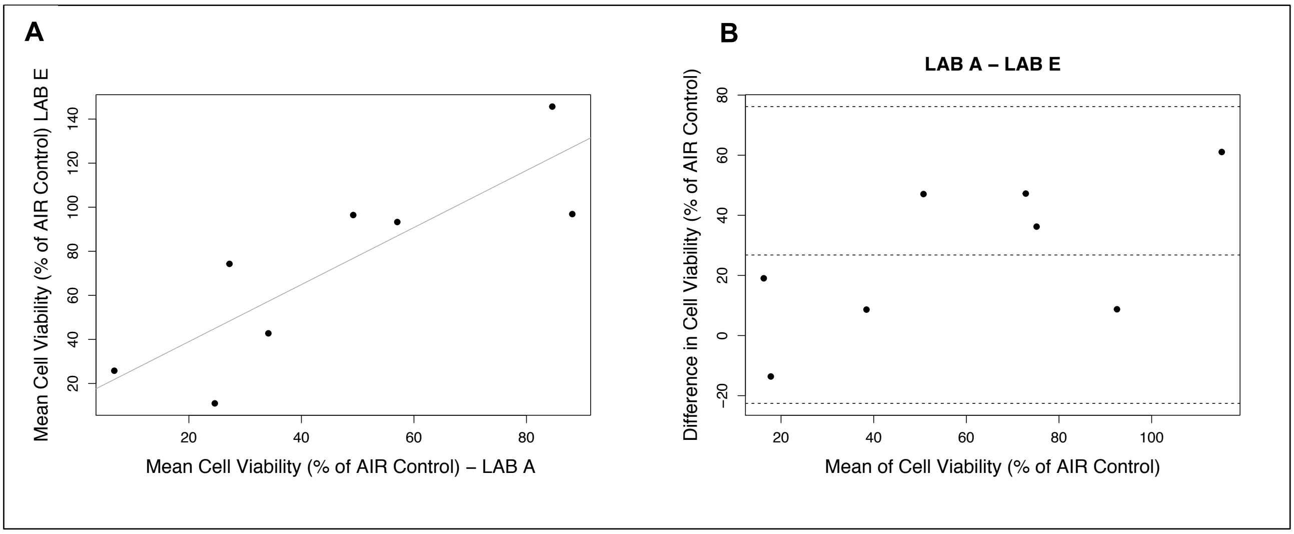

Figure S18. Assessment of reproducibility for NRU cell viability results after exposure to 1R6F ISO VP with different puff numbers. The left panel (**A**) shows the scatter plot of regression analysis of the Cell Viability Mean (% of AIR Control) between LAB-A and LAB-E (R= 0.715; p= 0.008). The right panel (**B**) shows the difference between the measurements obtained by LAB-A and LAB-E with respect to the mean in each ISO VP exposure condition (i.e. puff number) in the Bland Altman plot. No results had the Cell Viability (% of AIR Control) difference between LAB-A and LAB-E outside the 95% confidence interval. Graphs were generated with R version 3.4.3 (2017-11-30) and edited by using GIMP image manipulation program (version 2.10.14).

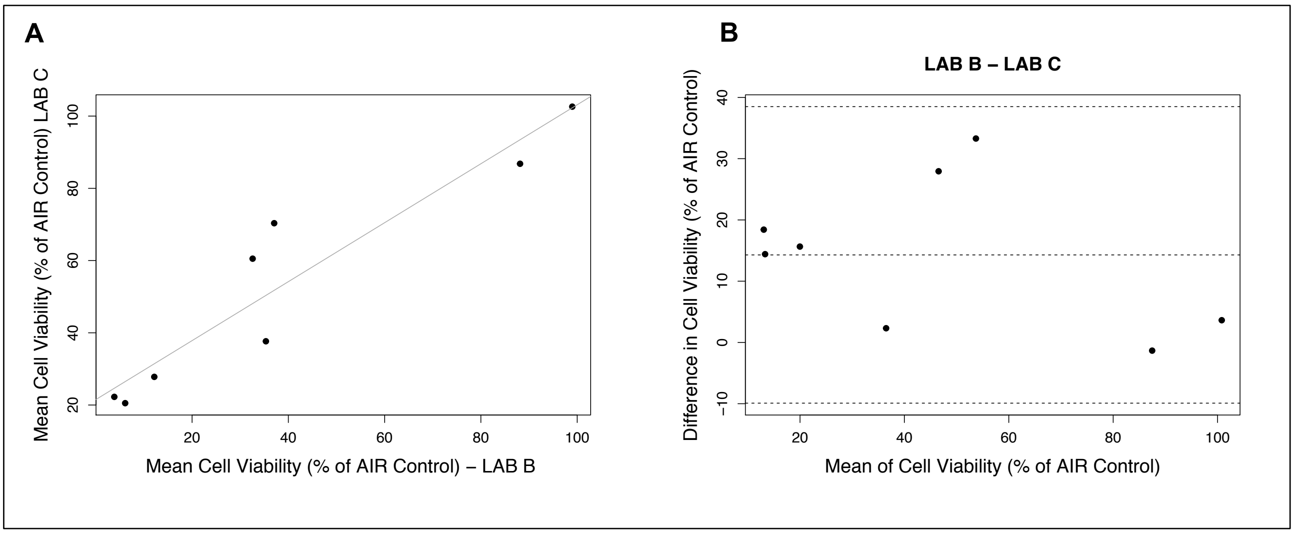

Figure S19. Assessment of reproducibility for NRU cell viability results after exposure to 1R6F ISO VP with different puff numbers. The left panel (**A**) shows the scatter plot of regression analysis of the Cell Viability Mean (% of AIR Control) between LAB-B and LAB-C (R= 0.889; p< 0.001). The right panel (**B**) shows the difference between the measurements obtained by LAB-B and LAB-C with respect to the mean in each ISO VP exposure condition (i.e. puff number) in the Bland Altman plot. No results had the Cell Viability (% of AIR Control) difference between LAB-B and LAB-C outside the 95% confidence interval. Graphs were generated with R version 3.4.3 (2017-11-30) and edited by using GIMP image manipulation program (version 2.10.14).

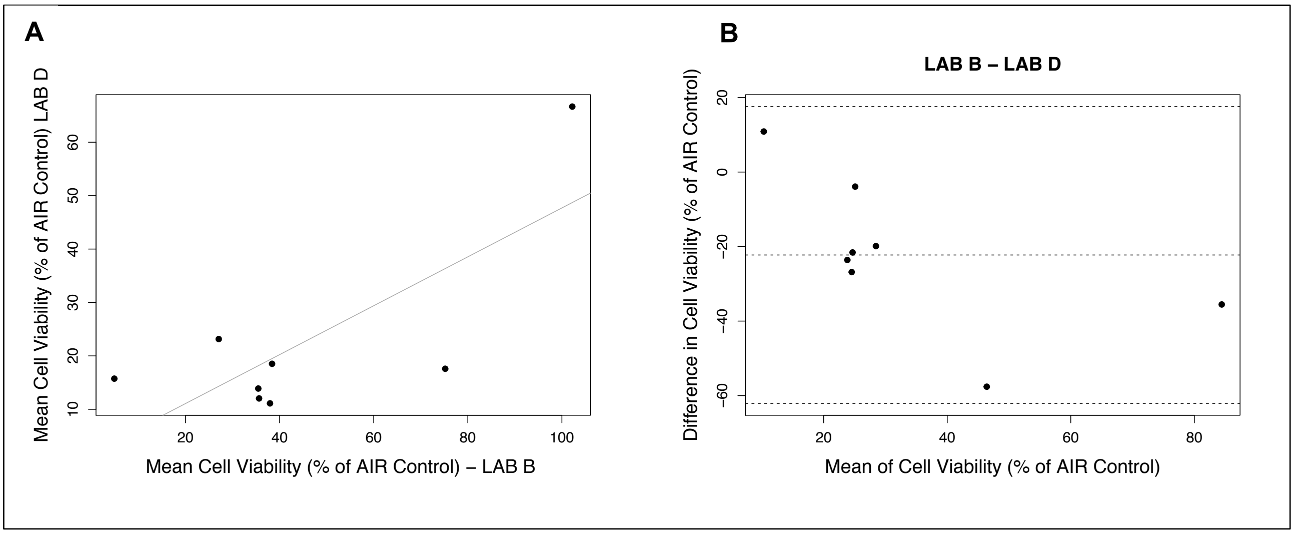

Figure S20. Assessment of reproducibility for NRU cell viability results after exposure to 1R6F ISO VP with different puff numbers. The left panel (**A**) shows the scatter plot of regression analysis of the Cell Viability Mean (% of AIR Control) between LAB-B and LAB-D (R= 0.502; p= 0.05). The right panel (**B**) shows the difference between the measurements obtained by LAB-B and LAB-D with respect to the mean in each ISO VP exposure condition (i.e. puff number) in the Bland Altman plot. No results had the Cell Viability (% of AIR Control) difference between LAB-B and LAB-D outside the 95% confidence interval. Graphs were generated with R version 3.4.3 (2017-11-30) and edited by using GIMP image manipulation program (version 2.10.14).

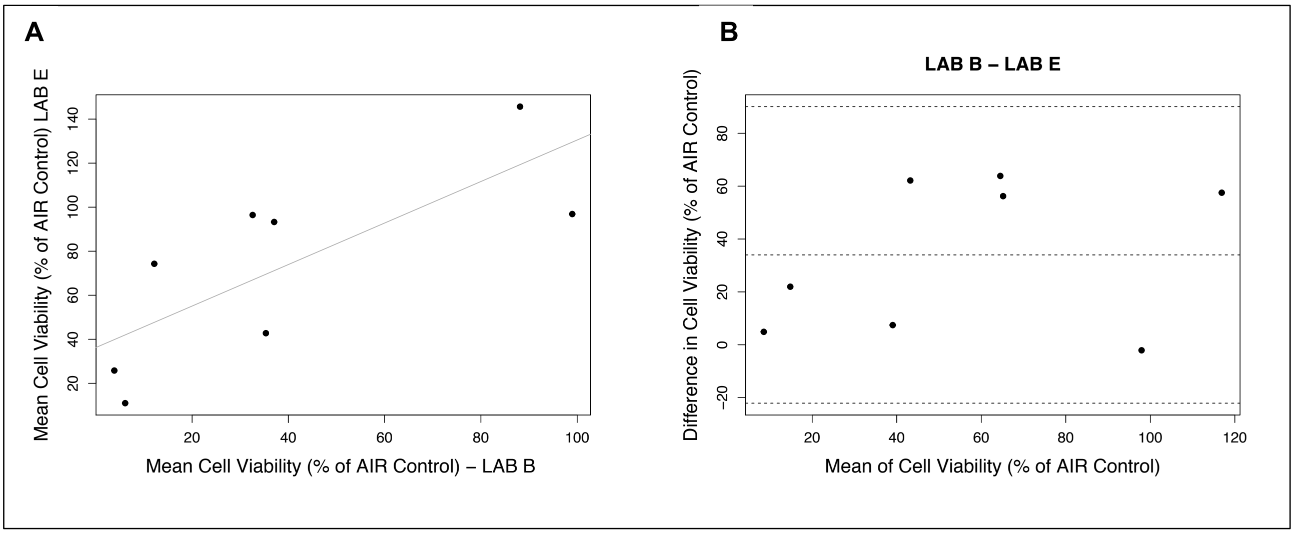

Figure S21. Assessment of reproducibility for NRU cell viability results after exposure to 1R6F ISO VP with different puff numbers. The left panel (**A**) shows the scatter plot of regression analysis of the Cell Viability Mean (% of AIR Control) between LAB-B and LAB-E (R= 0.587; p= 0.027). The right panel (**B**) shows the difference between the measurements obtained by LAB-B and LAB-E with respect to the mean in each ISO VP exposure condition (i.e. puff number) in the Bland Altman plot. No results had the Cell Viability (% of AIR Control) difference between LAB-E and LAB-D outside the 95% confidence interval. Graphs were generated with R version 3.4.3 (2017-11-30) and edited by using GIMP image manipulation program (version 2.10.14).

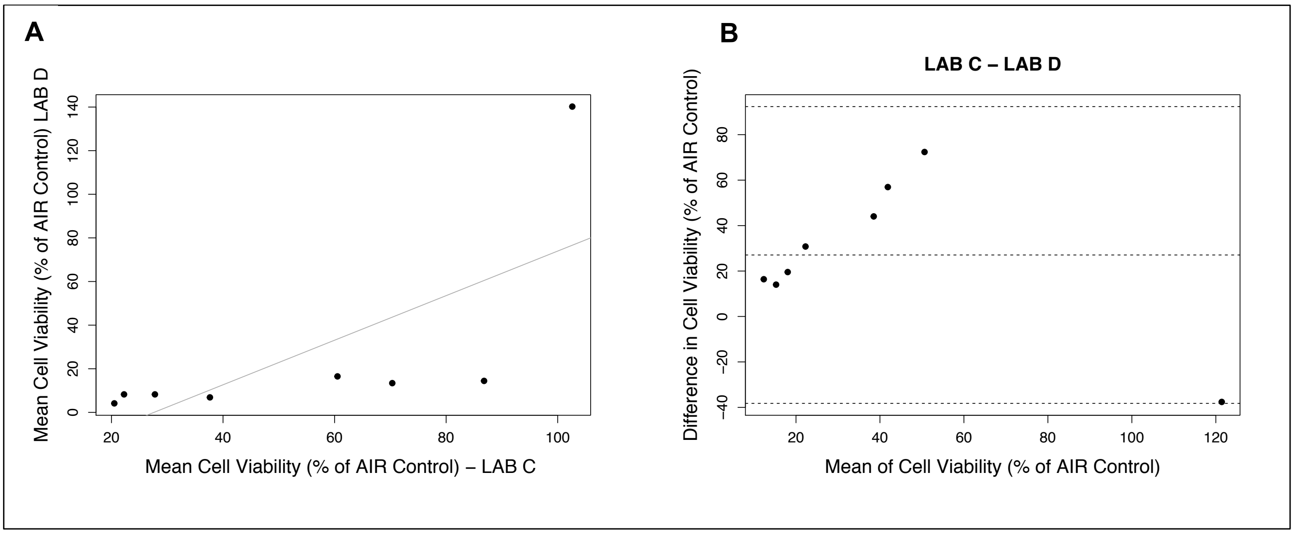

Figure S22. Assessment of reproducibility for NRU cell viability results after exposure to 1R6F ISO VP with different puff numbers. The left panel (**A**) shows the scatter plot of regression analysis of the Cell Viability Mean (% of AIR Control) between LAB-C and LAB-D (R= 0.479; p= 0.057). The right panel (**B**) shows the difference between the measurements obtained by LAB-C and LAB-D with respect to the mean in each ISO VP exposure condition (i.e. puff number) in the Bland Altman plot. No results had the Cell Viability (% of AIR Control) difference between LAB-C and LAB-D outside the 95% confidence interval. Graphs were generated with R version 3.4.3 (2017-11-30) and edited by using GIMP image manipulation program (version 2.10.14).

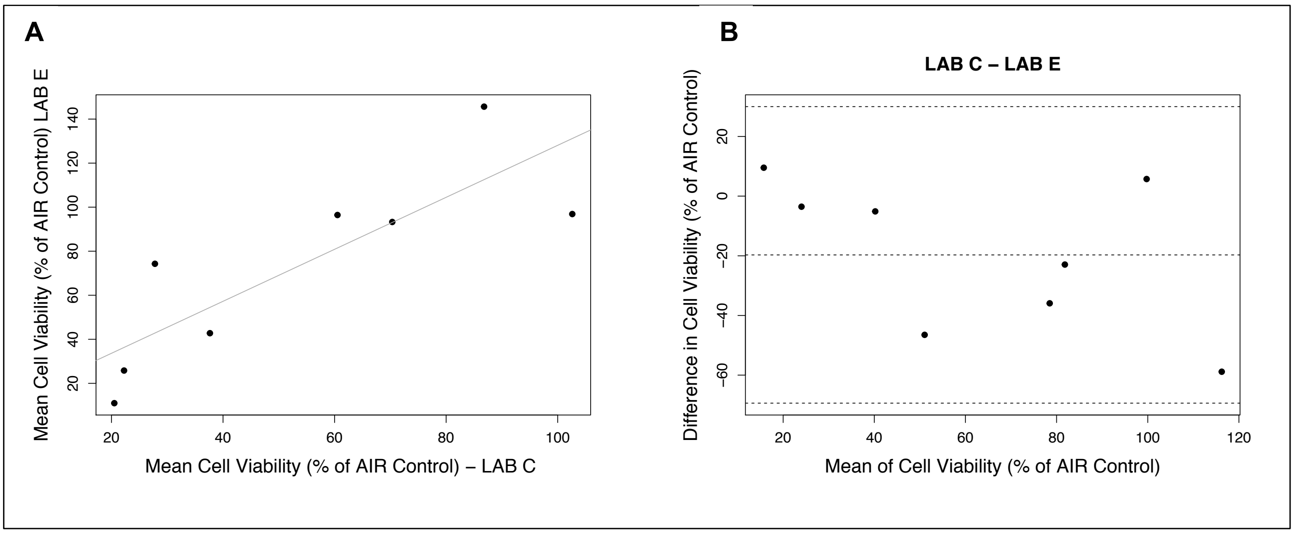

Figure S23. Assessment of reproducibility for NRU cell viability results after exposure to 1R6F ISO VP with different puff numbers. The left panel (**A**) shows the scatter plot of regression analysis of the Cell Viability Mean (% of AIR Control) between LAB-C and LAB-E (R= 0.69; p= 0.011). The right panel (**B**) shows the difference between the measurements obtained by LAB-C and LAB-E with respect to the mean in each ISO VP exposure condition (i.e. puff number) in the Bland Altman plot. No results had the Cell Viability (% of AIR Control) difference between LAB-C and LAB-E outside the 95% confidence interval. Graphs were generated with R version 3.4.3 (2017-11-30) and edited by using GIMP image manipulation program (version 2.10.14).

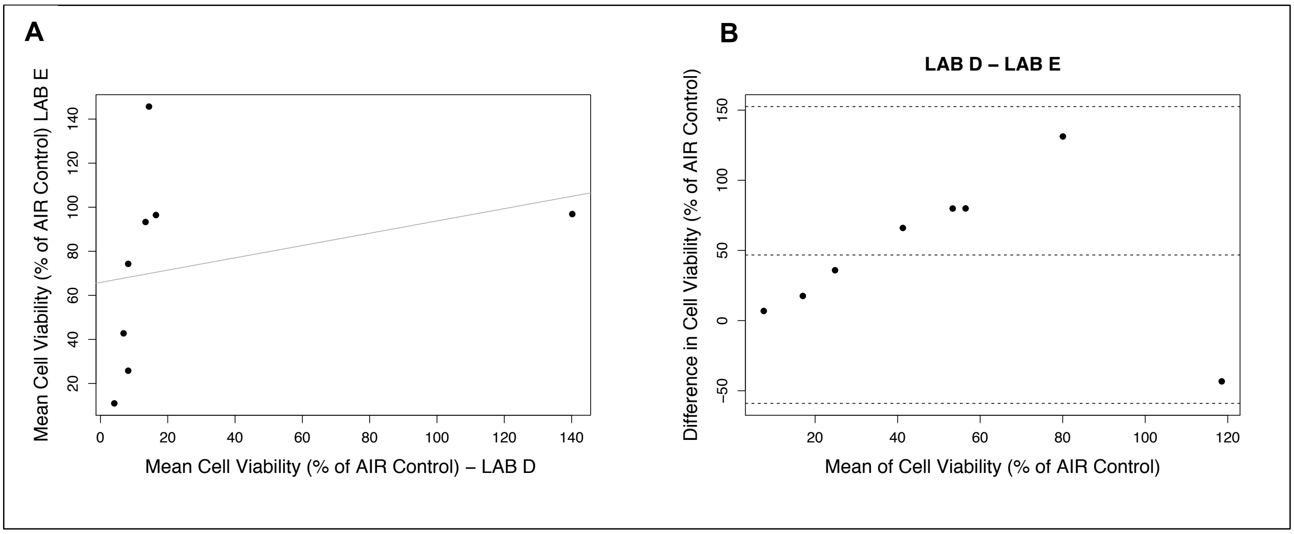

Figure S24. Assessment of reproducibility for NRU cell viability results after exposure to 1R6F ISO VP with different puff numbers. The left panel (**A**) shows the scatter plot of regression analysis of the Cell Viability Mean (% of AIR Control) between LAB-D and LAB-E (R= 0.084; p= 0.486). The right panel (**B**) shows the difference between the measurements obtained by LAB-D and LAB-E with respect to the mean in each ISO VP exposure condition (i.e. puff number) in the Bland Altman plot. No results had the Cell Viability (% of AIR Control) difference between LAB-D and LAB-E outside the 95% confidence interval. Graphs were generated with R version 3.4.3 (2017-11-30) and edited by using GIMP image manipulation program (version 2.10.14).

HCI Whole Smoke (WS) exposure

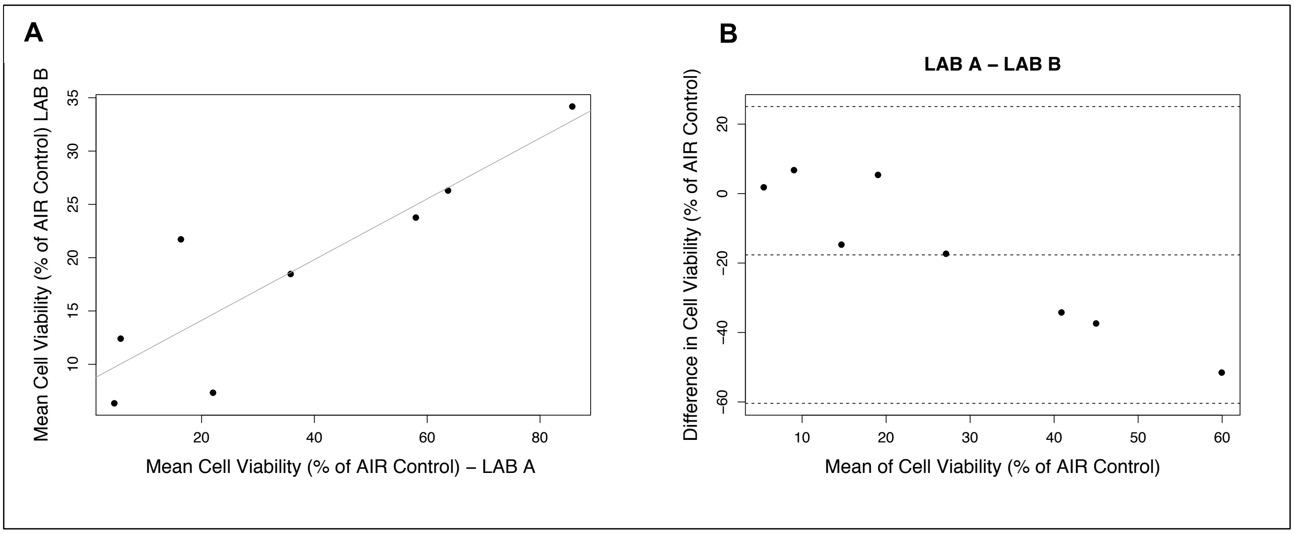

Figure S25. Assessment of reproducibility for NRU cell viability results after exposure to 1R6F HCI WS with different puff numbers. The left panel (**A**) shows the scatter plot of regression analysis of the Cell Viability Mean (% of AIR Control) between LAB-A and LAB-B (R= 0.77; p= 0.004). The right panel (**B**) shows the difference between the measurements obtained by LAB-A and LAB-B with respect to the mean in each HCI WS exposure condition (i.e. puff number) in the Bland Altman plot. No results had the Cell Viability (% of AIR Control) difference between LAB-A and LAB-B outside the 95% confidence interval. Graphs were generated with R version 3.4.3 (2017-11-30) and edited by using GIMP image manipulation program (version 2.10.14).

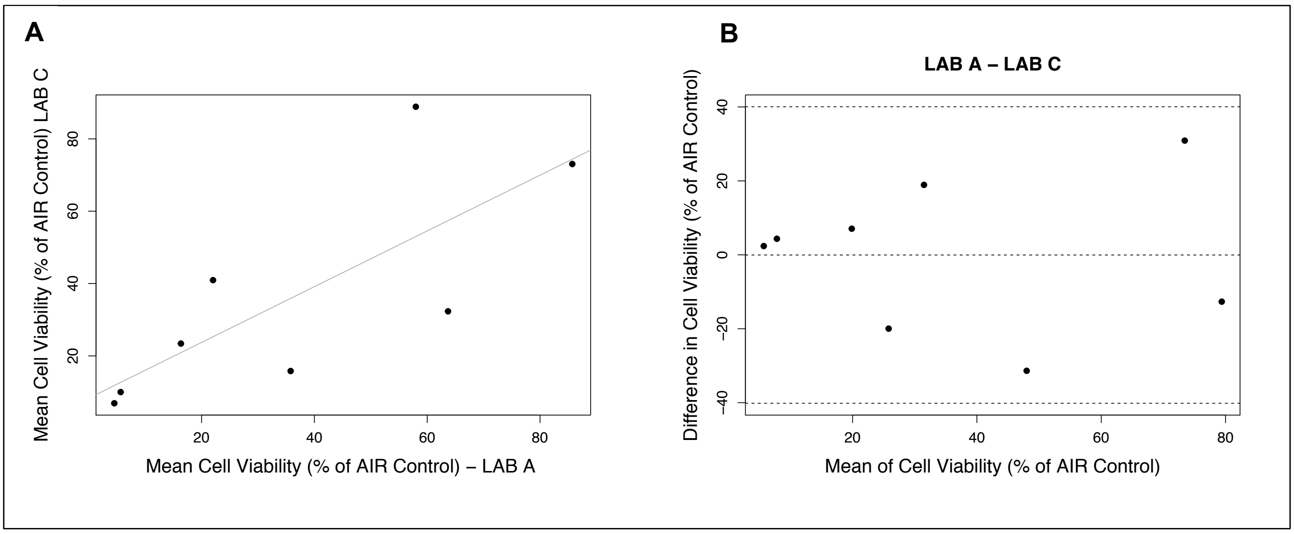

Figure S26. Assessment of reproducibility for NRU cell viability results after exposure to 1R6F HCI WS with different puff numbers. The left panel (**A**) shows the scatter plot of regression analysis of the Cell Viability Mean (% of AIR Control) between LAB-A and LAB-C (R= 0.586; p< 0.027). The right panel (**B**) shows the difference between the measurements obtained by LAB-A and LAB-C with respect to the mean in each HCI WS exposure condition (i.e. puff number) in the Bland Altman plot. No results had the Cell Viability (% of AIR Control) difference between LAB-A and LAB-C outside the 95% confidence interval. Graphs were generated with R version 3.4.3 (2017-11-30) and edited by using GIMP image manipulation program (version 2.10.14).

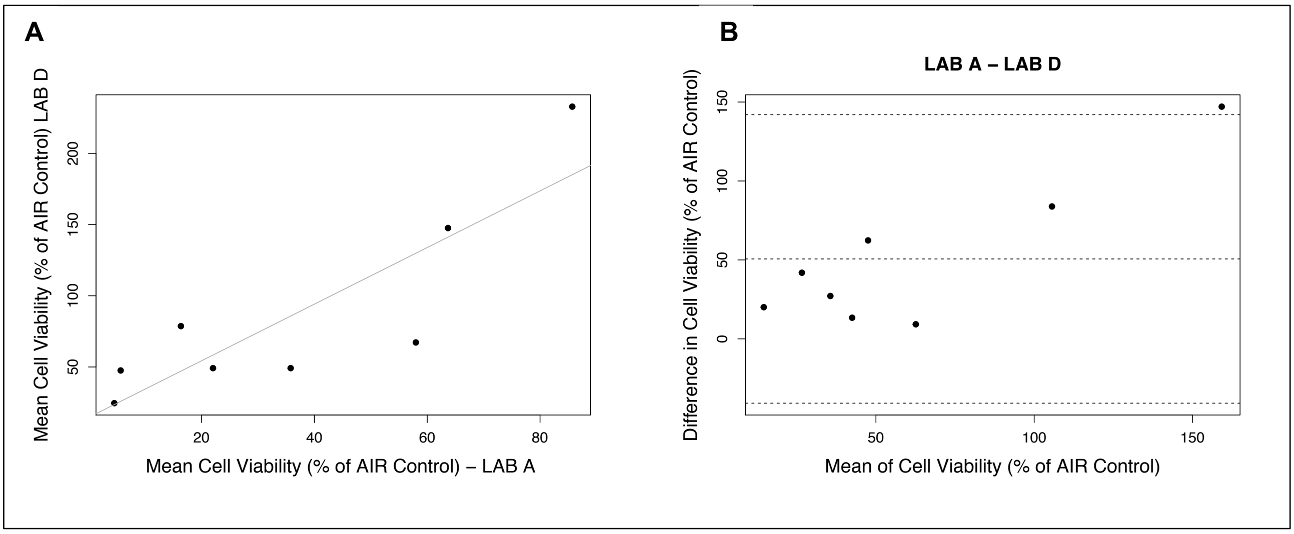

Figure S27. Assessment of reproducibility for NRU cell viability results after exposure to 1R6F HCI WS with different puff numbers. The left panel (**A**) shows the scatter plot of regression analysis of the Cell Viability Mean (% of AIR Control) between LAB-A and LAB-D (R= 0.729; p= 0.007). The right panel (**B**) shows the difference between the measurements obtained by LAB-A and LAB-D with respect to the mean in each HCI WS exposure condition (i.e. puff number) in the Bland Altman plot. One result had the Cell Viability (% of AIR Control) difference between LAB-A and LAB-D outside the 95% confidence interval. Graphs were generated with R version 3.4.3 (2017-11-30) and edited by using GIMP image manipulation program (version 2.10.14).

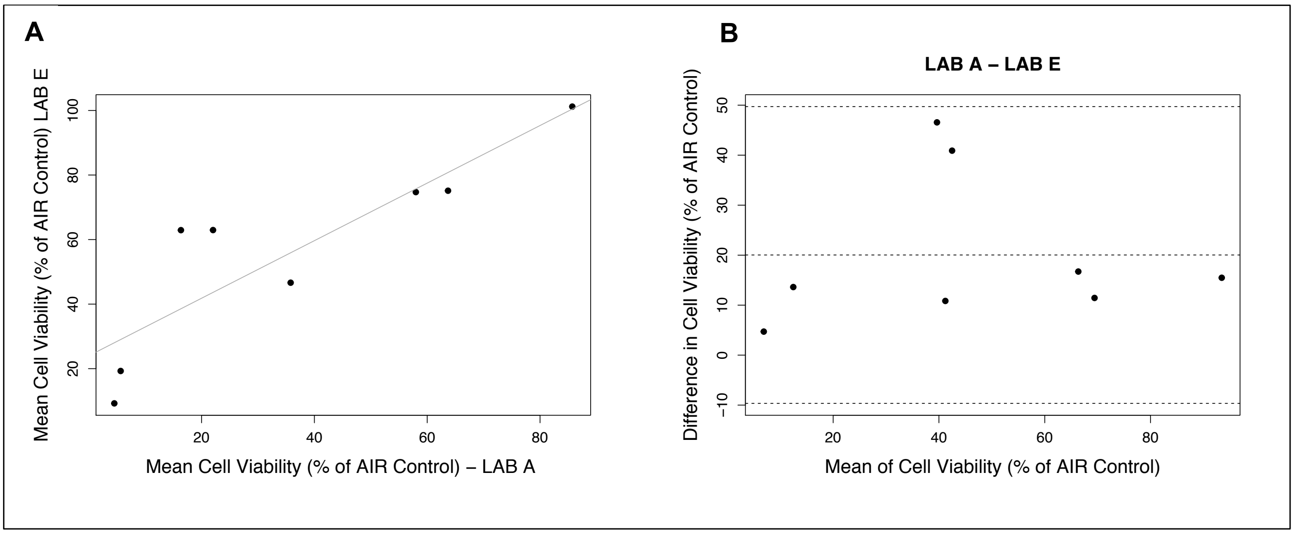

Figure S28. Assessment of reproducibility for NRU cell viability results after exposure to 1R6F HCI WS with different puff numbers. The left panel (**A**) shows the scatter plot of regression analysis of the Cell Viability Mean (% of AIR Control) between LAB-A and LAB-E (R= 0.763; p= 0.005). The right panel (**B**) shows the difference between the measurements obtained by LAB-A and LAB-E with respect to the mean in each HCI WS exposure condition (i.e. puff number) in the Bland Altman plot. No results had the Cell Viability (% of AIR Control) difference between LAB-A and LAB-E outside the 95% confidence interval. Graphs were generated with R version 3.4.3 (2017-11-30) and edited by using GIMP image manipulation program (version 2.10.14).

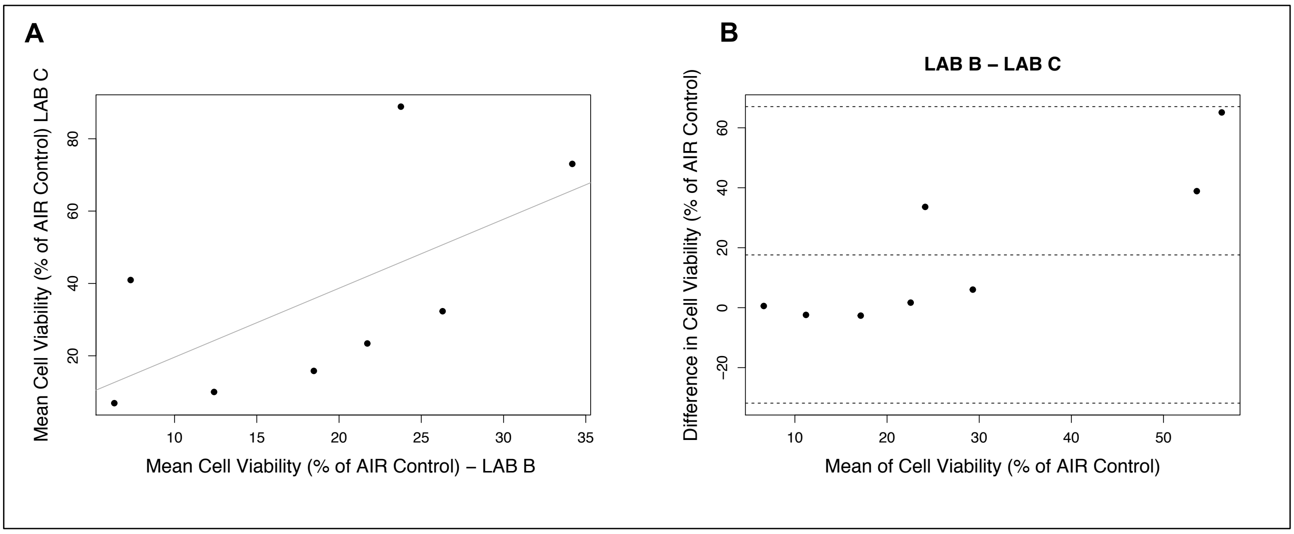

Figure S29. Assessment of reproducibility for NRU cell viability results after exposure to 1R6F HCI WS with different puff numbers. The left panel (**A**) shows the scatter plot of regression analysis of the Cell Viability Mean (% of AIR Control) between LAB-B and LAB-C (R= 0.378; p= 0.105). The right panel (**B**) shows the difference between the measurements obtained by LAB-B and LAB-C with respect to the mean in each HCI WS exposure condition (i.e. puff number) in the Bland Altman plot. No results had the Cell Viability (% of AIR Control) difference between LAB-B and LAB-C outside the 95% confidence interval. Graphs were generated with R version 3.4.3 (2017-11-30) and edited by using GIMP image manipulation program (version 2.10.14).

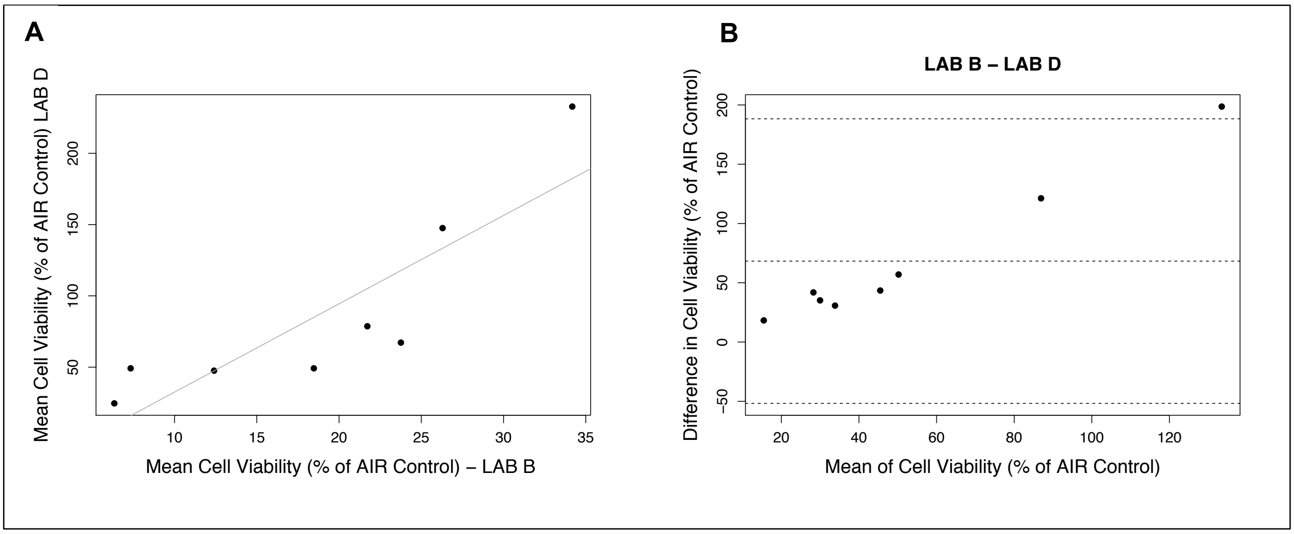

Figure S30. Assessment of reproducibility for NRU cell viability results after exposure to 1R6F HCI WS with different puff numbers. The left panel (**A**) shows the scatter plot of regression analysis of the Cell Viability Mean (% of AIR Control) between LAB-B and LAB-D (R= 0.745; p= 0.006). The right panel (**B**) shows the difference between the measurements obtained by LAB-B and LAB-D with respect to the mean in each HCI WS exposure condition (i.e. puff number) in the Bland Altman plot. One result had the Cell Viability (% of AIR Control) difference between LAB-B and LAB-D outside the 95% confidence interval. Graphs were generated with R version 3.4.3 (2017-11-30) and edited by using GIMP image manipulation program (version 2.10.14).

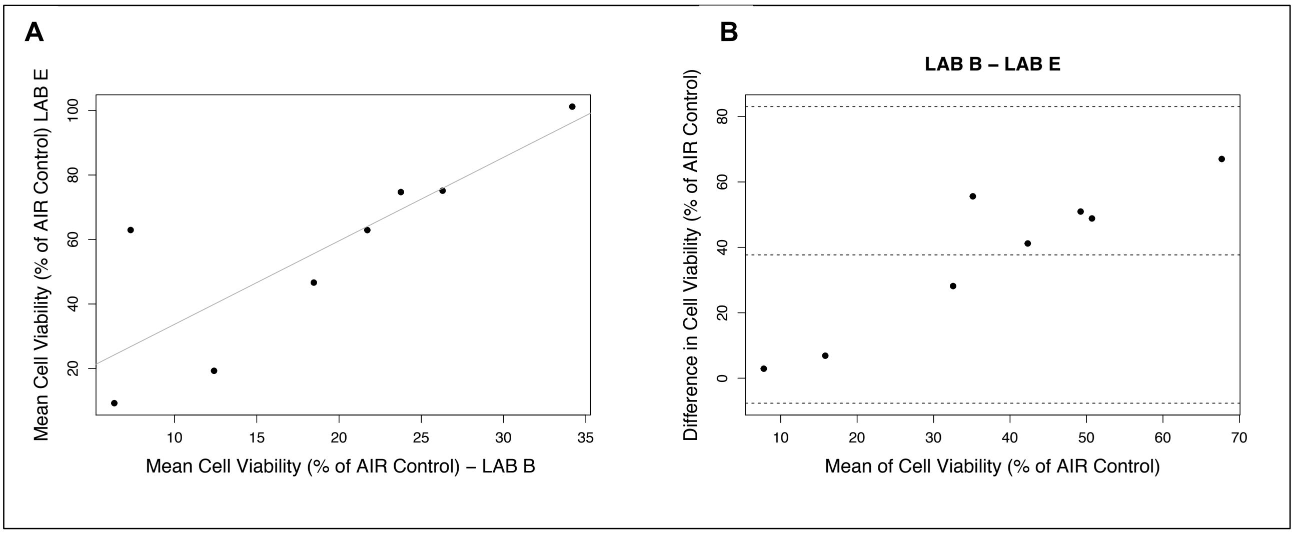

Figure S31. Assessment of reproducibility for NRU cell viability results after exposure to 1R6F HCI WS with different puff numbers. The left panel (**A**) shows the scatter plot of regression analysis of the Cell Viability Mean (% of AIR Control) between LAB-B and LAB-E (R= 0.677; p= 0.012). The right panel (**B**) shows the difference between the measurements obtained by LAB-B and LAB-E with respect to the mean in each HCI WS exposure condition (i.e. puff number) in the Bland Altman plot. No results had the Cell Viability (% of AIR Control) difference between LAB-E and LAB-D outside the 95% confidence interval. Graphs were generated with R version 3.4.3 (2017-11-30) and edited by using GIMP image manipulation program (version 2.10.14).

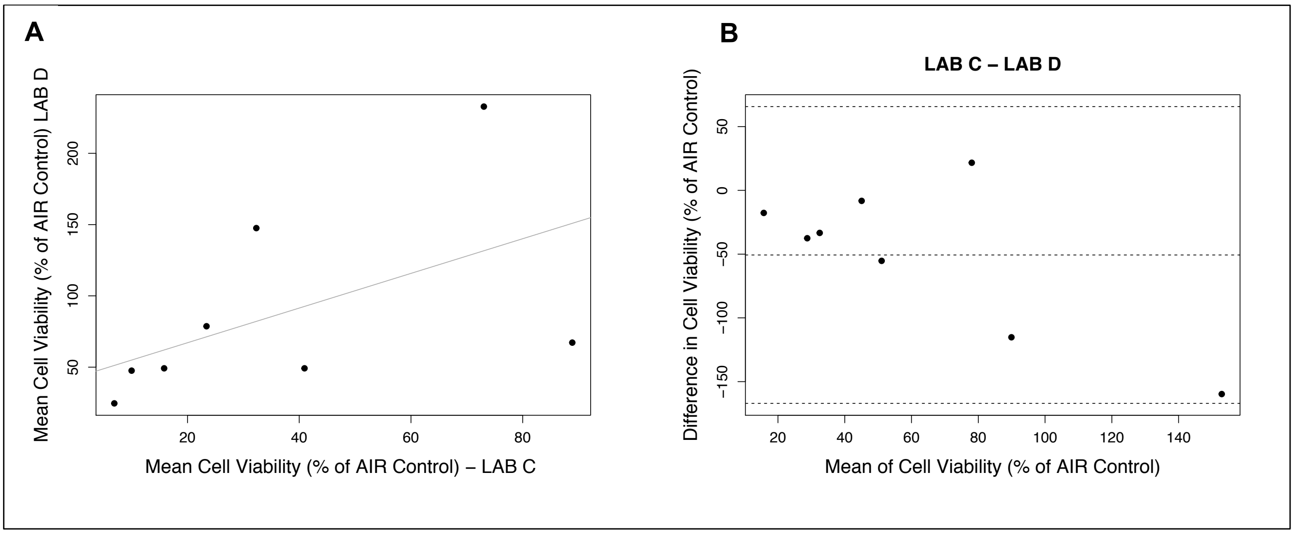

Figure S32. Assessment of reproducibility for NRU cell viability results after exposure to 1R6F HCI WS with different puff numbers. The left panel (**A**) shows the scatter plot of regression analysis of the Cell Viability Mean (% of AIR Control) between LAB-C and LAB-D (R= 0.276; p= 0.181). The right panel (**B**) shows the difference between the measurements obtained by LAB-C and LAB-D with respect to the mean in each HCI WS exposure condition (i.e. puff number) in the Bland Altman plot. No results had the Cell Viability (% of AIR Control) difference between LAB-C and LAB-D outside the 95% confidence interval. Graphs were generated with R version 3.4.3 (2017-11-30) and edited by using GIMP image manipulation program (version 2.10.14).

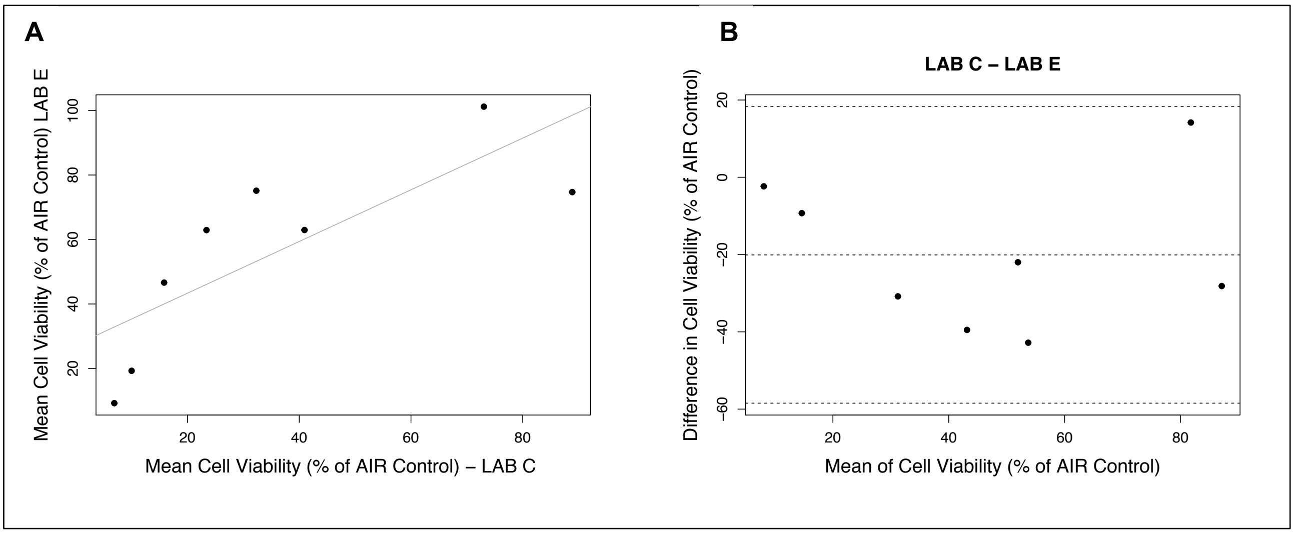

Figure S33. Assessment of reproducibility for NRU cell viability results after exposure to 1R6F HCI WS with different puff numbers. The left panel (**A**) shows the scatter plot of regression analysis of the Cell Viability Mean (% of AIR Control) between LAB-C and LAB-E (R= 0.624; p= 0.02). The right panel (**B**) shows the difference between the measurements obtained by LAB-C and LAB-E with respect to the mean in each HCI WS exposure condition (i.e., puff number) in the Bland Altman plot. No results had the Cell Viability (% of AIR Control) difference between LAB-C and LAB-E outside the 95% confidence interval. Graphs were generated with R version 3.4.3 (2017-11-30) and edited by using GIMP image manipulation program (version 2.10.14).

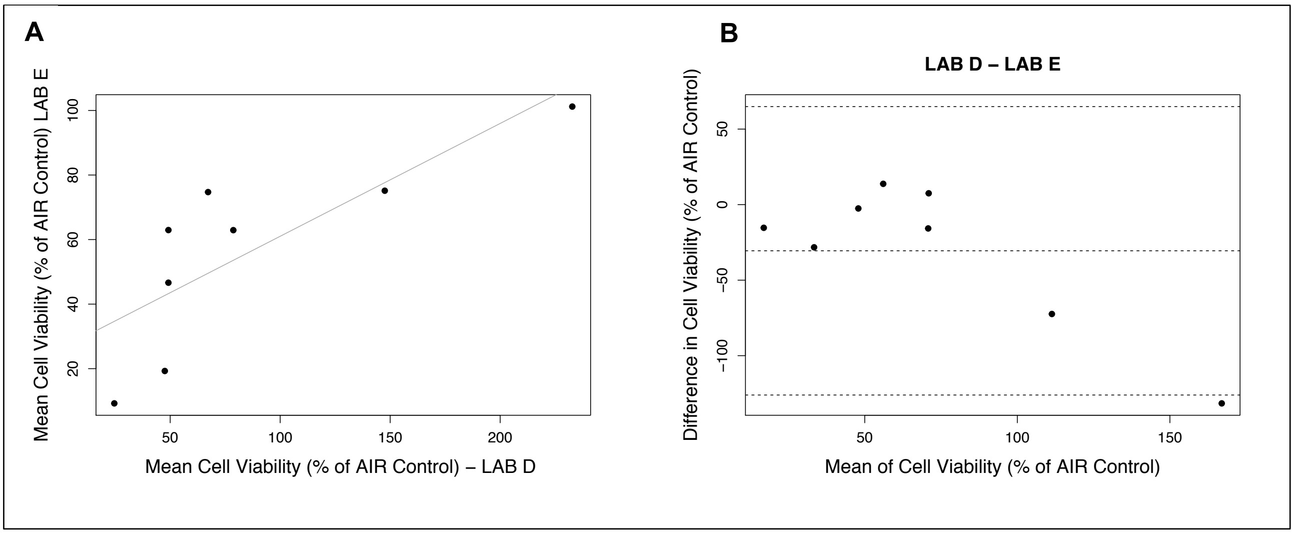

Figure S34. Assessment of reproducibility for NRU cell viability results after exposure to 1R6F HCI WS with different puff numbers. The left panel (**A**) shows the scatter plot of regression analysis of the Cell Viability Mean (% of AIR Control) between LAB-D and LAB-E (R= 0.636; p= 0.018). The right panel (**B**) shows the difference between the measurements obtained by LAB-D and LAB-E with respect to the mean in each HCI WS exposure condition (i.e. puff number) in the Bland Altman plot. One result had the Cell Viability (% of AIR Control) difference between LAB-D and LAB-E outside the 95% confidence interval. Graphs were generated with R version 3.4.3 (2017-11-30) and edited by using GIMP image manipulation program (version 2.10.14).

HCI Vapour Phase (VP) exposure

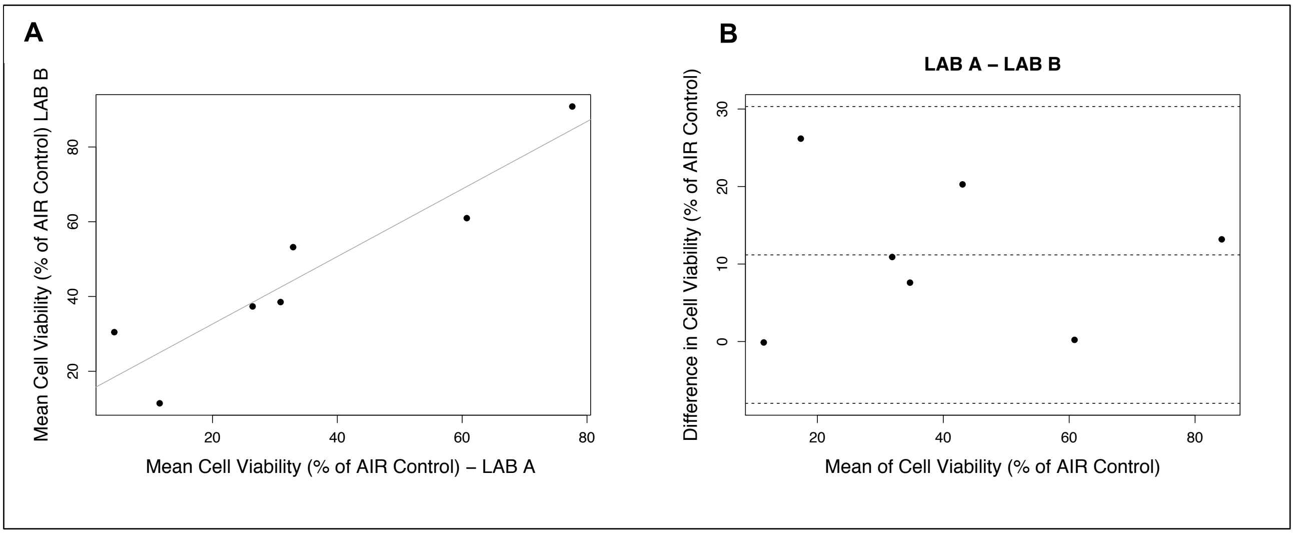

Figure S35. Assessment of reproducibility for NRU cell viability results after exposure to 1R6F HCI VP with different puff numbers. The left panel (**A**) shows the scatter plot of regression analysis of the Cell Viability Mean (% of AIR Control) between LAB-A and LAB-B (R= 0.861; p= 0.003). The right panel (**B**) shows the difference between the measurements obtained by LAB-A and LAB-B with respect to the mean in each HCI VP exposure condition (i.e. puff number) in the Bland Altman plot. No results had the Cell Viability (% of AIR Control) difference between LAB-A and LAB-B outside the 95% confidence interval. Graphs were generated with R version 3.4.3 (2017-11-30) and edited by using GIMP image manipulation program (version 2.10.14).

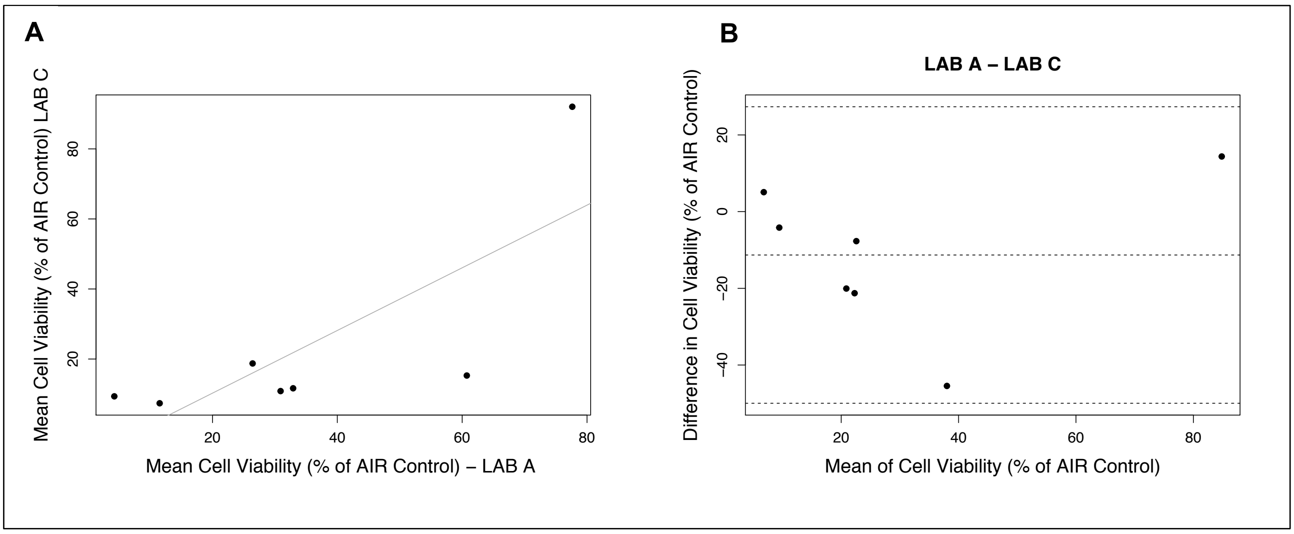

Figure S36. Assessment of reproducibility for NRU cell viability results after exposure to 1R6F HCI VP with different puff numbers. The left panel (**A**) shows the scatter plot of regression analysis of the Cell Viability Mean (% of AIR Control) between LAB-A and LAB-C (R= 0.587; p< 0.044). The right panel (**B**) shows the difference between the measurements obtained by LAB-A and LAB-C with respect to the mean in each HCI VP exposure condition (i.e. puff number) in the Bland Altman plot. No results had the Cell Viability (% of AIR Control) difference between LAB-A and LAB-C outside the 95% confidence interval. Graphs were generated with R version 3.4.3 (2017-11-30) and edited by using GIMP image manipulation program (version 2.10.14).

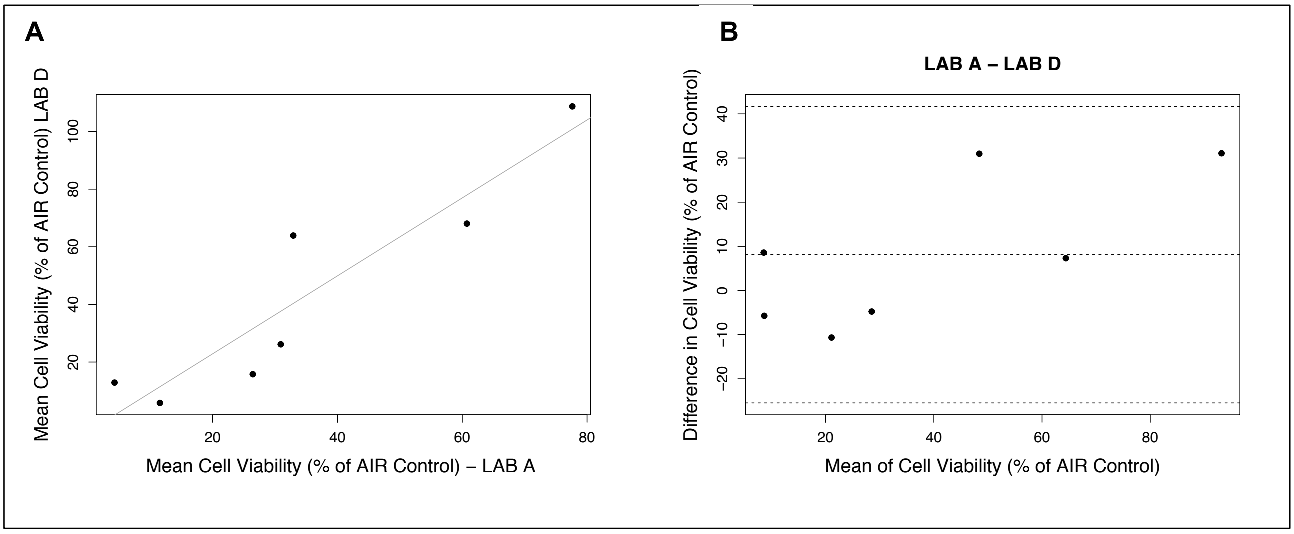

Figure S37. Assessment of reproducibility for NRU cell viability results after exposure to 1R6F HCI VP with different puff numbers. The left panel (**A**) shows the scatter plot of regression analysis of the Cell Viability Mean (% of AIR Control) between LAB-A and LAB-D (R= 0.856; p= 0.003). The right panel (**B**) shows the difference between the measurements obtained by LAB-A and LAB-D with respect to the mean in each HCI VP exposure condition (i.e. puff number) in the Bland Altman plot. No results had the Cell Viability (% of AIR Control) difference between LAB-A and LAB-D outside the 95% confidence interval. Graphs were generated with R version 3.4.3 (2017-11-30) and edited by using GIMP image manipulation program (version 2.10.14).

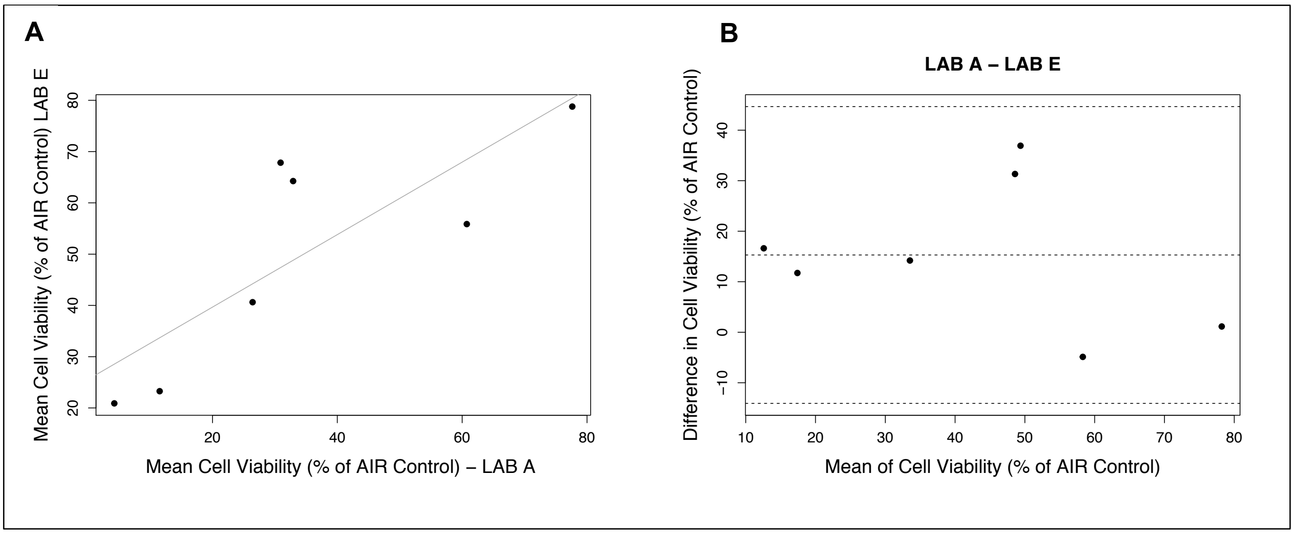

Figure S38. Assessment of reproducibility for NRU cell viability results after exposure to 1R6F HCI VP with different puff numbers. The left panel (**A**) shows the scatter plot of regression analysis of the Cell Viability Mean (% of AIR Control) between LAB-A and LAB-E (R= 0.671; p= 0.024). The right panel (**B**) shows the difference between the measurements obtained by LAB-A and LAB-E with respect to the mean in each HCI VP exposure condition (i.e. puff number) in the Bland Altman plot. No results had the Cell Viability (% of AIR Control) difference between LAB-A and LAB-E outside the 95% confidence interval. Graphs were generated with R version 3.4.3 (2017-11-30) and edited by using GIMP image manipulation program (version 2.10.14).

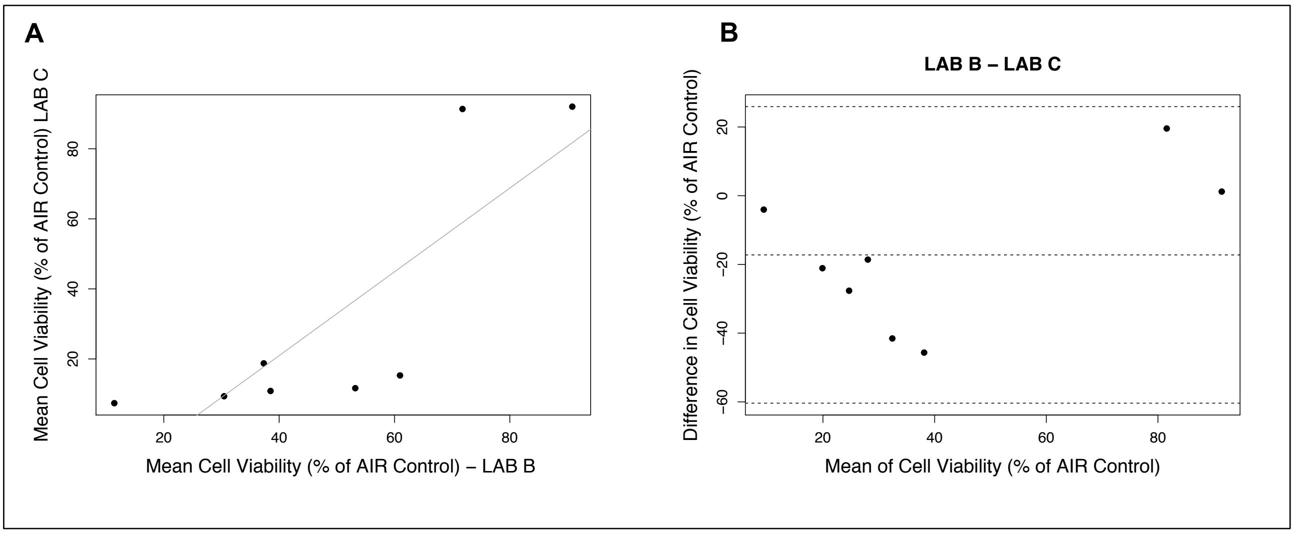

Figure S39. Assessment of reproducibility for NRU cell viability results after exposure to 1R6F HCI VP with different puff numbers. The left panel (**A**) shows the scatter plot of regression analysis of the Cell Viability Mean (% of AIR Control) between LAB-B and LAB-C (R= 0.663; p= 0.014). The right panel (**B**) shows the difference between the measurements obtained by LAB-B and LAB-C with respect to the mean in each HCI VP exposure condition (i.e. puff number) in the Bland Altman plot. No results had the Cell Viability (% of AIR Control) difference between LAB-B and LAB-C outside the 95% confidence interval. Graphs were generated with R version 3.4.3 (2017-11-30) and edited by using GIMP image manipulation program (version 2.10.14).

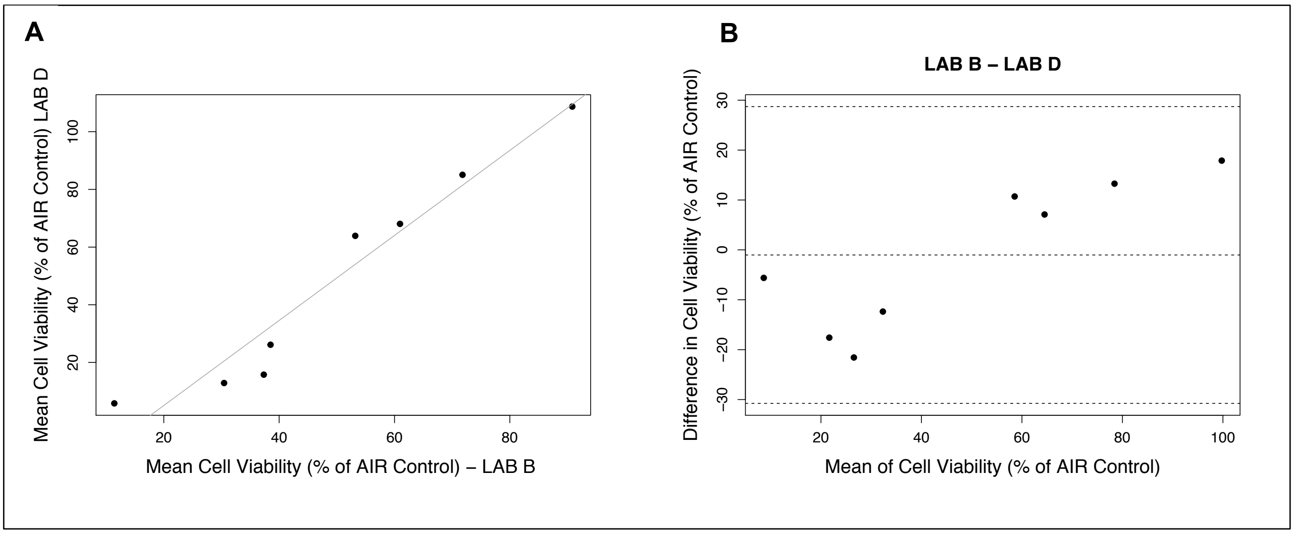

Figure S40. Assessment of reproducibility for NRU cell viability results after exposure to 1R6F HCI VP with different puff numbers. The left panel (**A**) shows the scatter plot of regression analysis of the Cell Viability Mean (% of AIR Control) between LAB-B and LAB-D (R= 0.94; p< 0.0001). The right panel (**B**) shows the difference between the measurements obtained by LAB-B and LAB-D with respect to the mean in each HCI VP exposure condition (i.e. puff number) in the Bland Altman plot. No results had the Cell Viability (% of AIR Control) difference between LAB-B and LAB-D outside the 95% confidence interval. Graphs were generated with R version 3.4.3 (2017-11-30) and edited by using GIMP image manipulation program (version 2.10.14).

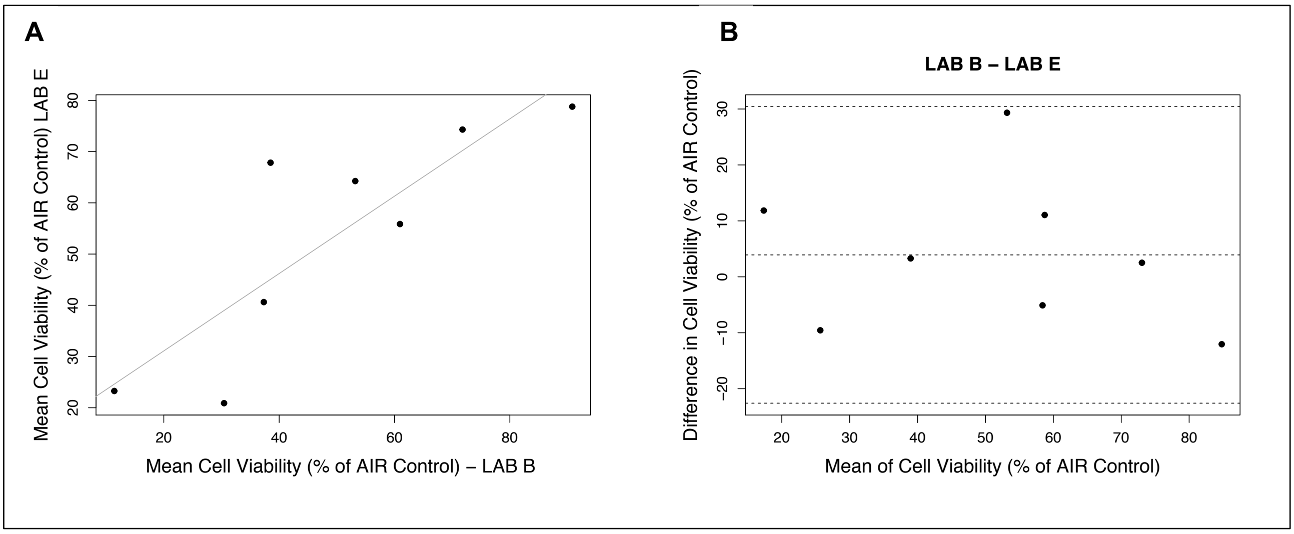

Figure S41. Assessment of reproducibility for NRU cell viability results after exposure to 1R6F HCI VP with different puff numbers. The left panel (**A**) shows the scatter plot of regression analysis of the Cell Viability Mean (% of AIR Control) between LAB-B and LAB-E (R= 0.714; p= 0.008). The right panel (**B**) shows the difference between the measurements obtained by LAB-B and LAB-E with respect to the mean in each HCI VP exposure condition (i.e. puff number) in the Bland Altman plot. No results had the Cell Viability (% of AIR Control) difference between LAB-E and LAB-D outside the 95% confidence interval. Graphs were generated with R version 3.4.3 (2017-11-30) and edited by using GIMP image manipulation program (version 2.10.14).

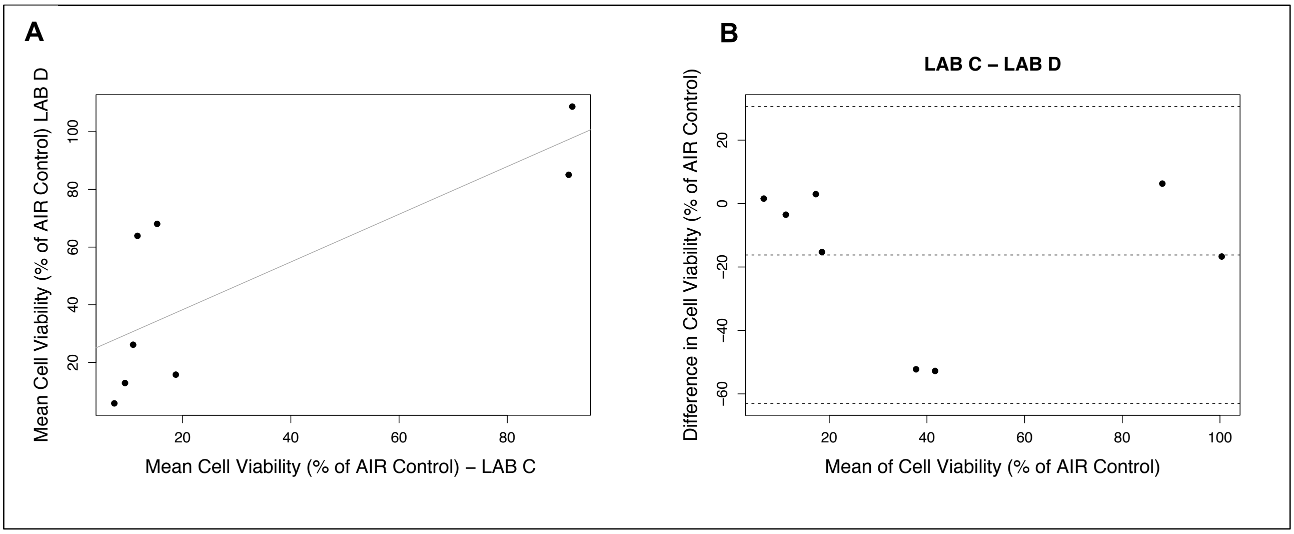

Figure S42. Assessment of reproducibility for NRU cell viability results after exposure to 1R6F HCI VP with different puff numbers. The left panel (**A**) shows the scatter plot of regression analysis of the Cell Viability Mean (% of AIR Control) between LAB-C and LAB-D (R= 0.639; p= 0.017). The right panel (**B**) shows the difference between the measurements obtained by LAB-C and LAB-D with respect to the mean in each HCI VP exposure condition (i.e. puff number) in the Bland Altman plot. No results had the Cell Viability (% of AIR Control) difference between LAB-C and LAB-D outside the 95% confidence interval. Graphs were generated with R version 3.4.3 (2017-11-30) and edited by using GIMP image manipulation program (version 2.10.14).

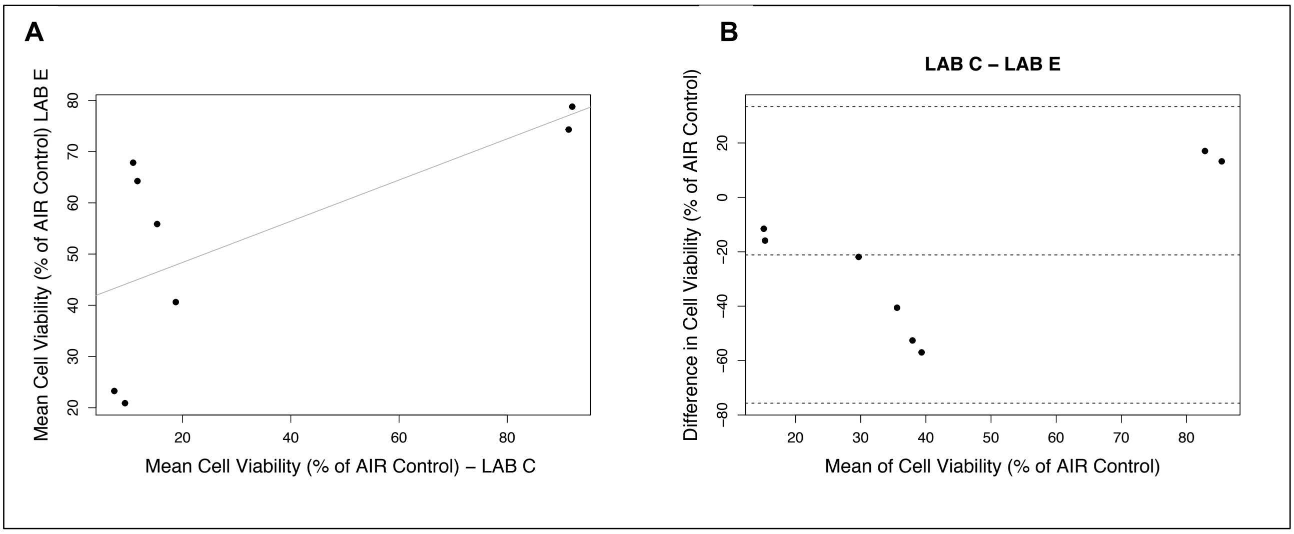

Figure S43. Assessment of reproducibility for NRU cell viability results after exposure to 1R6F HCI VP with different puff numbers. The left panel (**A**) shows the scatter plot of regression analysis of the Cell Viability Mean (% of AIR Control) between LAB-C and LAB-E (R= 0.437; p= 0.074). The right panel (**B**) shows the difference between the measurements obtained by LAB-C and LAB-E with respect to the mean in each HCI VP exposure condition (i.e., puff number) in the Bland Altman plot. No results had the Cell Viability (% of AIR Control) difference between LAB-C and LAB-E outside the 95% confidence interval. Graphs were generated with R version 3.4.3 (2017-11-30) and edited by using GIMP image manipulation program (version 2.10.14).

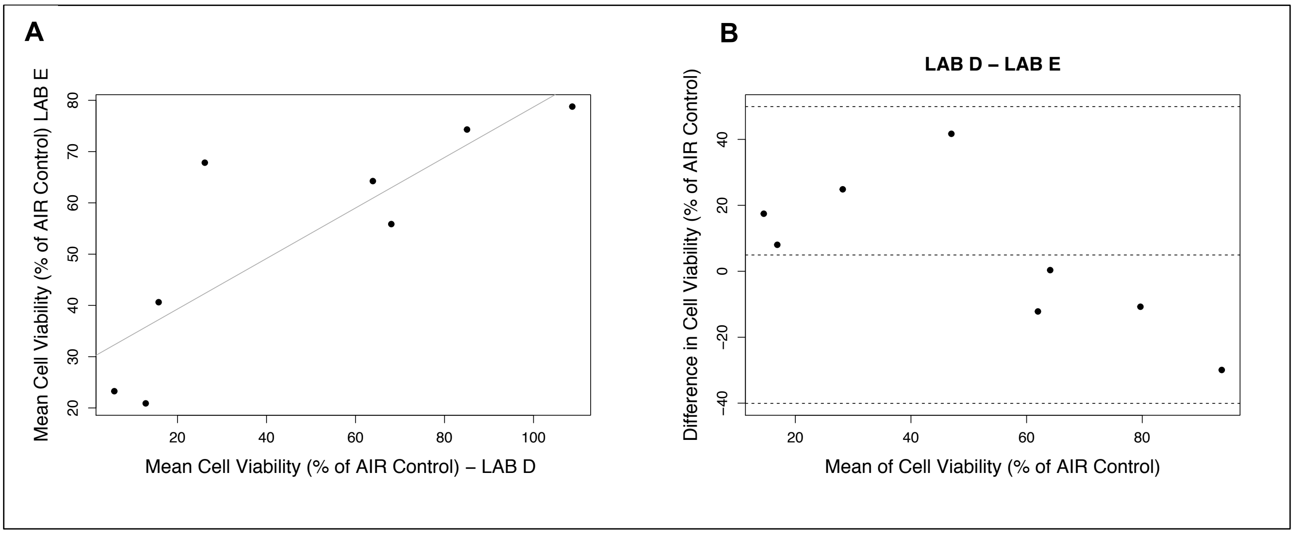

Figure S44. Assessment of reproducibility for NRU cell viability results after exposure to 1R6F HCI VP with different puff numbers. The left panel (**A**) shows the scatter plot of regression analysis of the Cell Viability Mean (% of AIR Control) between LAB-D and LAB-E (R= 0.702; p= 0.009). The right panel (**B**) shows the difference between the measurements obtained by LAB-D and LAB-E with respect to the mean in each HCI VP exposure condition (i.e. puff number) in the Bland Altman plot. No results had the Cell Viability (% of AIR Control) difference between LAB-D and LAB-E outside the 95% confidence interval. Graphs were generated with R version 3.4.3 (2017-11-30) and edited by using GIMP image manipulation program (version 2.10.14).

Evaluation of H292 cell morphology after exposure to 1R6F smoke and ENDS aerosol

Methods and results

At the end of each exposure to cigarette smoke, ENDS aerosol and air, H292 cells were trypsinized, counted and seeded in a 96-well (CellCarrier™-96; PerkinElmer #6005550) in triplicate at the density of $1*10^3$ cells/well and then placed in the incubator (5%CO_2; 37°C) for 24 h. Then cells were labeled for nuclei (NucBlue™ Live cell Stain, Thermo-Fisher Scientific #R37605), and membranes (CellMask™ Green Plasma Thermo-Fisher #C37608). Plate was read by using the 63x long WD objective (Operetta CLS™ high-content analysis system). Harmony high-content imaging and analysis software (PerkinElmer) was used to analyze all images by measuring the following morphological features: cell volume, cell surface area, cell sphericity, nucleus volume, nucleus surface area, nucleus sphericity, and nucleus/cytoplasm ratio. All values were expressed as mean fluorescence intensity (MFI).

Exposure of H292 cells to 1R6F smoke induced morphological changes of cells with reduced cell volume, nucleus volume, nucleus surface area, nucleus sphericity, and nucleus/cytoplasm ratio. No significant differences were observed in cell surface area and cell sphericity.

Table S2. Comparison of cell and nucleus morphological parameters on H292 cells.

	**AIR (25 puffs)**	**1R6F (5 puffs)**	**IQOS (7 puffs)**	**Glo (8 puffs)**	**ePen (10 puffs)**	**eStick (25 puffs)**	**p value**
Cell morphology Volume [μm^3]	1903.3±95.5	1411.0±167.3	1713.7±46.8	1735.7±30.9	1988.0±39.1	1988.0±39.1	<0.0001
Cell morphology Surface Area [μm^2]	1527.0±27.0	1463.0±216.3	1531.0±7.8	1433.3±20.1	1450.0±25.2	1536.3±25.4	0.36
Cell morphology Sphericity	0.48±0.01	0.48±0.02	0.49±0.01	0.48±0.002	0.48±0.003	0.49±0.003	0.34
Nucleus morphology Volume [μm^3]	772.8±15.7	307.28±16.1	776.6±5.9	720.3±7.26	724.7±7.9	781.1±23.9	<0.0001
Nucleus morphology Surface Area [μm^2]	647.4±7.1	415.94±10.97	652.6±9.1	636.2±10.03	638.06±3.5	653.9±18.9	<0.0001
Nucleus morphology Sphericity	0.61±0.009	0.53±0.02	0.61±0.008	0.60±0.006	0.60±0.004	0.61±0.006	<0.0001
Nucleus/Cytoplasm ratio	0.68±0.03	0.28±0.04	0.64±0.02	0.72±0.02	0.71±0.01	0.64±0.01	<0.0001

Data are reported as Mean ± SD of Mean Fluorescence Intensity (MFI). Overall p values were calculated by applying one-way ANOVA.

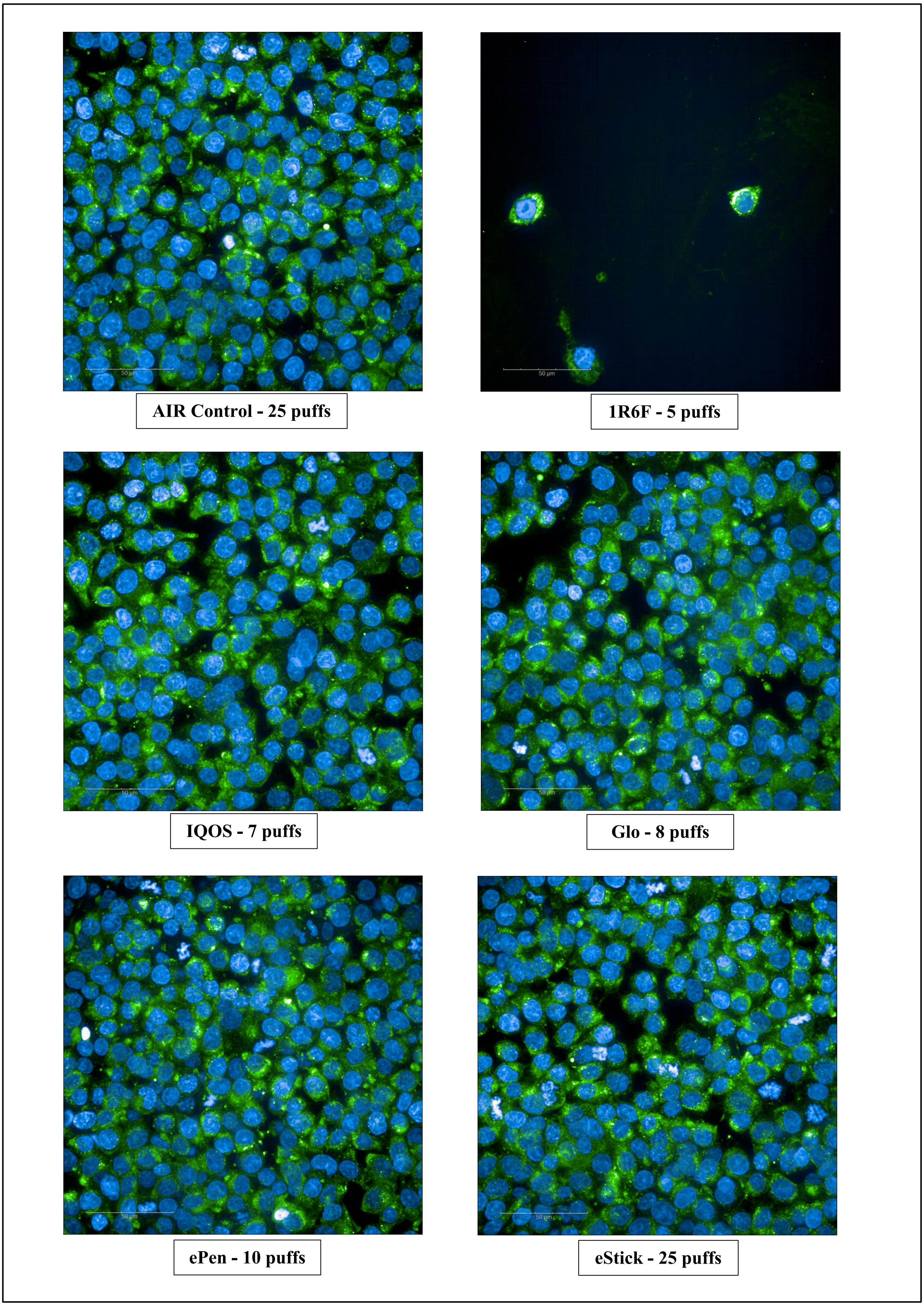

Figure S45. Representative images of cell and nucleus morphology evaluation on H292 cells. Images were obtained with the Operetta CLS™ high-content analysis system by using a 63x long WD objective, and edited by using GIMP image manipulation program (version 2.10.14)

Evaluation of inflammatory biomarkers on H292 cells after exposure to 1R6F smoke and ENDS aerosol

NCI-H292 cells were exposed to cigarette smoke, ENDS aerosol and air, and then incubated at 37°C in 5% CO_2 atmosphere with fresh media for 24 hours. Then, exposed H292 cells were washed twice with PBS and detached from the inserts with 0.25 % trypsin-EDTA. Samples in triplicate were incubated in flow cytometry staining buffer for 15 min at 4 °C with the following monoclonal antibodies: anti-mouse CD206 (Beckman coulter, IM2741, clone 3.29B1.10), and HLA-DR (Beckman coulter, A07793, clone Immu-357) antibodies. Flow cytometry analysis was performed with MACSQuant Analyzer (Milteny Biotech) using the Flowlogic software (Miltenyi Biotech).

All the results of CD206 and HLA-DR cell expression were reported in table S3. The expression of CD206 and HLA-DR in H292 cells after 1R6F exposure (5 puffs) were significantly increased ($p<0.0001$) compared to all the other exposure conditions (Figure S46). No significant differences of CD206 and HLA-DR were observed among IQOS, Glo, ePen, eStick, and controls.

Table S3. Expression of CD206 and HLA-DR marker on H292 cells

	AIR	1R6F	IQOS	Glo	ePen	eStick	p value
CD206	0.74±0.01	11.71±0.09	0.79±0.04	0.77±0.02	0.71±0.02	0.76±0.02	<0.0001
HLA-DR	0.36±0.02	2.75±0.04	0.39±0.03	0.41±0.02	0.38±0.01	0.39±0.02	<0.0001

Data are reported as Mean ± SD of Median Fluorescence Intensity (MFI). Overall p values were calculated by applying one-way ANOVA.

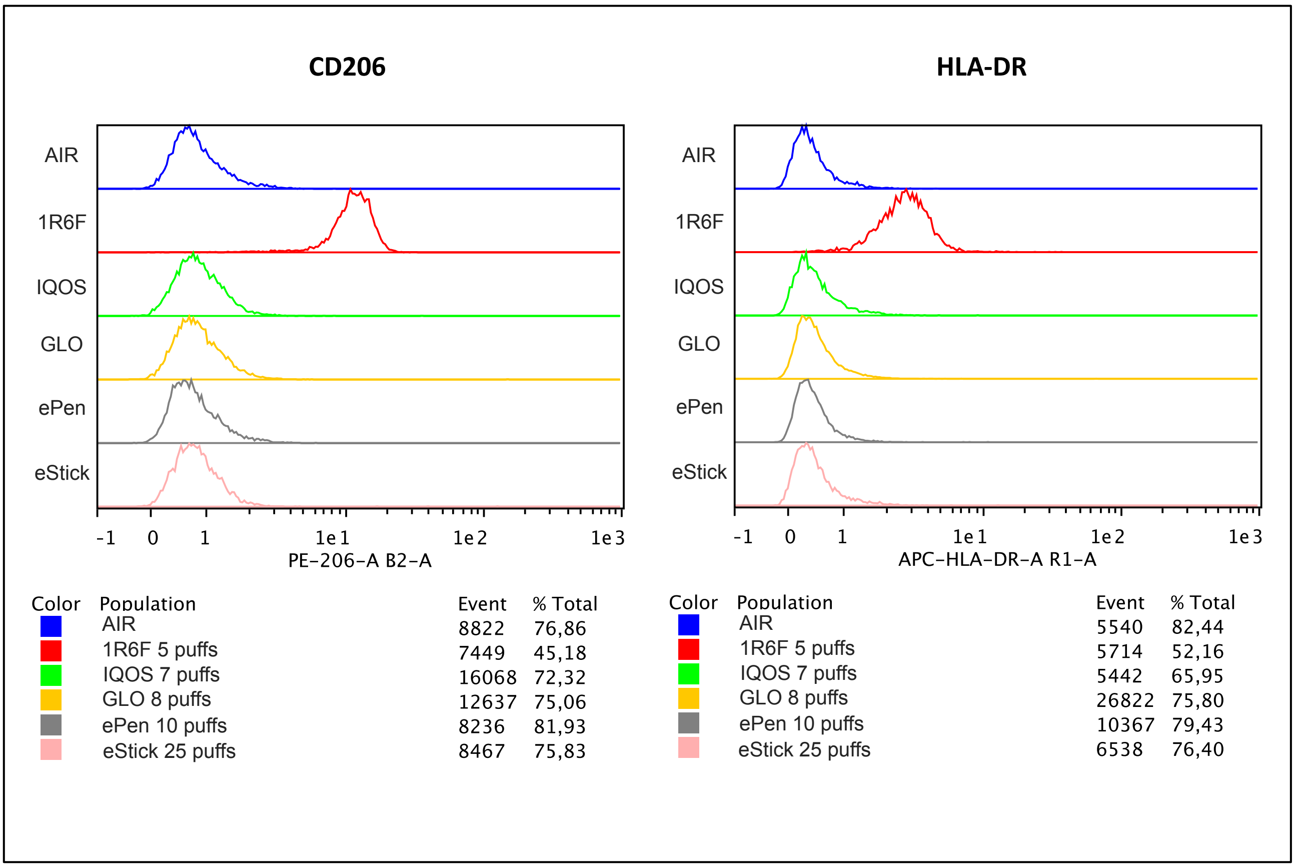

Figure S46. Representative images of CD206 and HLA-DR expression on H292 cells. Graphs were generated with Flowlogic software (Miltenyi Biotech), and edited by using GIMP image manipulation program (version 2.10.14)
